# Risk Factors Associated with Antibiotic Exposure Variability in Critically Ill Patients: A Systematic Review

**DOI:** 10.3390/antibiotics13090801

**Published:** 2024-08-24

**Authors:** Laura Gras-Martín, Adrián Plaza-Diaz, Borja Zarate-Tamames, Paula Vera-Artazcoz, Olga H. Torres, Carla Bastida, Dolors Soy, Jesús Ruiz-Ramos

**Affiliations:** 1 Pharmacy Department, Hospital de la Santa Creu i Sant Pau, Sant Antoni Maria Claret 167, 08025 Barcelona, Spain; lgras@santpau.cat (L.G.-M.); aplaza@santpau.cat (A.P.-D.); bzarate@santpau.cat (B.Z.-T.); 2 Institut de Recerca Sant Pau (IR SANT PAU), Sat Quintí 77-79, 08041 Barcelona, Spain; pvera@santpau.cat (P.V.-A.); or otorres@santpau.cat (O.H.T.); 3 Department of Medicine, Universitat Autònoma de Barcelona, 08193 Bellaterra, Spain; 4 Intensive Care Department, Hospital de la Santa Creu i Sant Pau, Sant Antoni Maria Claret 167, 08025 Barcelona, Spain; 5 Geriatric Unit, Internal Medicine Department, Hospital de la Santa Creu i Sant Pau, Sant Antoni Maria Claret 167, 08025 Barcelona, Spain; 6 Pharmacy Department, Division of Medicines, Hospital Clinic of Barcelona, Villarroel 170, 08036 Barcelona, Spain; cbastida@clinic.cat (C.B.);; 7 Department of Pharmacology, Toxicology and Therapeutical Chemistry, Faculty of Pharmacy, Universitat de Barcelona, Campus Diagonal, Av. de Joan XXIII, 27-31, 08028 Barcelona, Spain

**Keywords:** pharmacokinetics, pharmacodynamics, critically ill patients, antibiotic, exposure, target attainment, risk factors

## Abstract

(1) Background: Knowledge about the behavior of antibiotics in critically ill patients has been increasing in recent years. Some studies have concluded that a high percentage may be outside the therapeutic range. The most likely cause of this is the pharmacokinetic variability of critically ill patients, but it is not clear which factors have the greatest impact. The aim of this systematic review is to identify risk factors among critically ill patients that may exhibit significant pharmacokinetic alterations, compromising treatment efficacy and safety. (2) Methods: The search included the PubMed, Web of Science, and Embase databases. (3) Results: We identified 246 observational studies and ten clinical trials. The most studied risk factors in the literature were renal function, weight, age, sex, and renal replacement therapy. Risk factors with the greatest impact included renal function, weight, renal replacement therapy, age, protein or albumin levels, and APACHE or SAPS scores. (4) Conclusions: The review allows us to identify which critically ill patients are at a higher risk of not reaching therapeutic targets and helps us to recognize the extensive number of risk factors that have been studied, guiding their inclusion in future studies. It is essential to continue researching, especially in real clinical practice and with clinical outcomes.

## 1. Introduction

Sepsis is a life-threatening organ dysfunction, impacting millions of people around the world each year and killing one in three to one in six of those it affects. Early administration of appropriate antimicrobial treatment is one of the most effective interventions to reduce mortality in patients with sepsis. Therefore, it is essential to carry out effective and safe anti-infective treatment [1].

Knowing the optimal dosage of these drugs is very complex because, unlike other treatments, the pharmacological effect cannot be evaluated immediately. It is known that the behavior of drugs depends on the relationship between their pharmacokinetics and pharmacodynamics (PK/PD). In the case of antimicrobial agents, pharmacodynamics refers to the minimum inhibitory concentration (MIC) of the microorganism to be treated. This relationship has been extensively studied in recent years, especially in critically ill patients [2,3,4,5,6,7]. This knowledge, along with the increase in resistance, has led to the discovery that the initially assumed PK/PD targets are not sufficient to resolve the infection. Studies conducted on the PK/PD of antibiotics in critically ill patients have made it possible to define specific therapeutic targets for these patients [8,9,10,11,12,13,14].

The pharmacokinetics of the anti-infective agents are crucial for attaining their optimal effect, and we know that they are highly variable in critically ill patients [15,16,17,18,19,20,21]. When a patient is admitted to the intensive care unit (ICU), significant physiological changes occur that are generally not considered in the design of dosing regimens. The factors influencing changes in pharmacokinetics vary across different sources, and there is a lack of consistent data on the true impact and magnitude of each factor. Some of these factors include renal replacement therapy (RRT), extracorporeal membrane oxygenation (ECMO), obesity, aging, or comorbidities, which are also increasing in these units [22,23,24,25,26,27,28,29,30,31,32]. In fact, several studies have concluded that high percentages of critically ill patients on antibiotic treatment have plasma concentrations outside the therapeutic range, including several groups of antibiotics [33,34,35,36,37,38]. 

Despite this information, standard doses are still frequently used for these patients, with little consensus on the appropriate dosing [39,40]. The only way to ensure that patients achieve adequate antibiotic exposure and thus avoid therapeutic failures and side effects is through the determination of plasma concentration. However, this practice is not available for all patients in most centers due to costs and lack of evidence on clinical outcomes [10,41,42,43,44,45,46,47,48,49,50,51,52,53]. Most of these studies exhibit considerable variability in terms of the drugs studied, infections, causative microorganisms, and patient characteristics. In ICUs, we find very heterogeneous patients, and it is likely that pharmacokinetic variability will not be the same in all of them, nor will the impact on dosing, antibiotic exposure, and clinical outcomes.

The aim of this systematic review is to identify risk factors in critically ill patients who may present relevant pharmacokinetic alterations that compromise the efficacy and safety of the treatment.

## 2. Results

**Figure 1:**
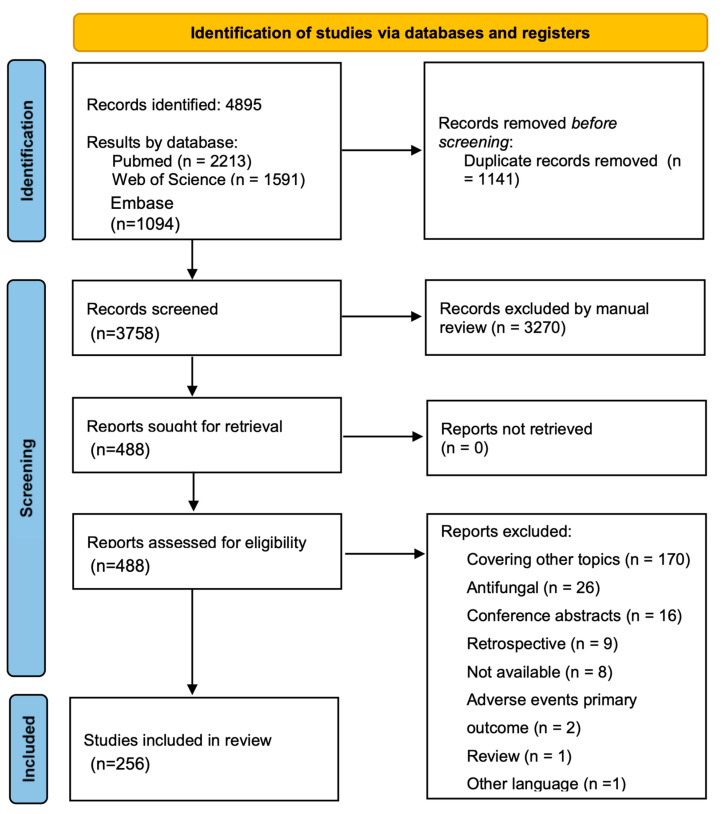
Search results. PRISMA Flow Diagram.

A total of 4895 articles were identified through computer searches in the selected databases, with 1141 duplicates removed through electronic or manual methods. A total of 3754 studies were screened by title and abstract and 489 were assessed for eligibility by full-text assessment. A total of 256 studies were finally included in the systematic review (Figure 1). Detailed reporting quality and risk-of-bias assessments are presented in Figures S1 and S2 (Supplementary Materials; File S3).

Most of the studies found were prospective, observational, and single-center studies. Different types of studies could be distinguished based on whether the outcome was to evaluate pharmacokinetic variability (evaluate alterations in the clearance and/or volume of distribution), develop a population pharmacokinetic model (PKPOP) (develop pharmacokinetic models tailored to critically ill patients using specialized software such as Nonmem^®^), or assess antibiotic exposure variability (evaluate variations in antimicrobial concentrations without assessing pharmacokinetic parameters). Only nine studies (3.50%) also evaluated clinical outcomes. The average number of patients per study was 85.3, ranging from three to 7220 patients. These studies were conducted practically worldwide, although the numbers varied widely by region. They included studies on many different antibiotics, with studies on beta-lactams being prominent (n = 136), followed by vancomycin (n = 42). The remaining characteristics of the analyzed studies are presented in Table 1.

**Figure 2:**
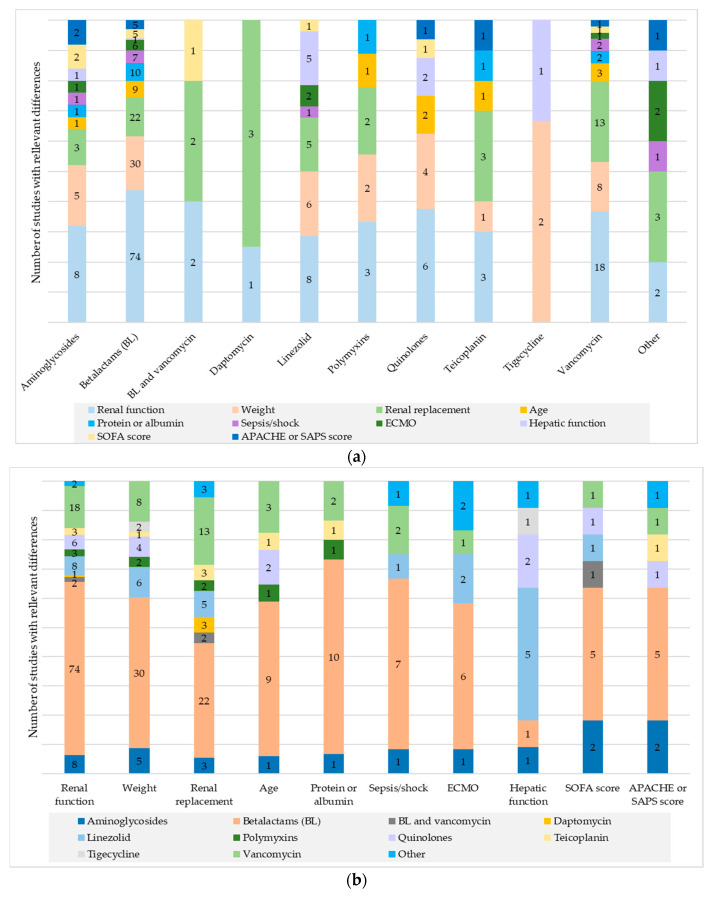
Number of studies with the ten most relevant risk factors. (**a**) For risk factors. (**b**) For antibiotics.

The most studied risk factors in the publications were renal function, weight, age, sex, and renal replacement. The risk factors that were found to have the most impact were renal function, weight, renal replacement, age, and protein or albumin levels, in that order. Looking at the risk factors by the percentage of studies that concluded differences versus the studies analyzed, the risk factors with the most impact were renal function, burns, acid–base parameters, trauma, and renal replacement. The risk factors studied in each study, the number of articles that analyzed them, and the number of articles that established them as determinants, classified by antibiotic group and for each outcome type (pharmacokinetic parameters or population model and exposure), are included in Table 2. The percentage of risk factors that had the greatest influence, represented by drug group, and the effect of each risk factor by drug group can be observed in Figure 2. Some studies do not conclude statistically significant differences but do determine that there is considerable variability. 

**Figure 3:**
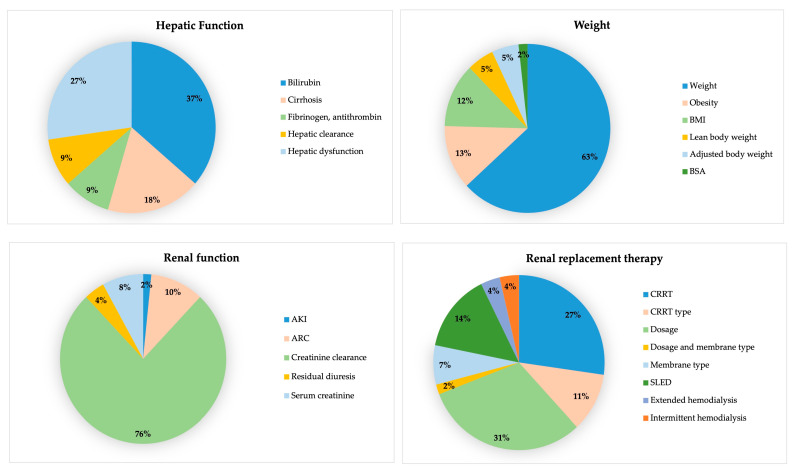
Details of variables included renal function, hepatic function, weight, and renal replacement therapy as risk factors. AKI: Acute kidney injury, ARC: Augmented renal clearance, BMI: body mass index, BSA: body surface area, CRRT: continuous renal replacement therapy, SLED: sustained low-efficiency dialysis.

Some of the risk factors include different characteristics. Sepsis or septic shock was assessed based on the presence of these conditions, the use of vasopressors, or mean arterial pressure, while acute phase reactants were evaluated using C-reactive protein, fever, procalcitonin, or leukocyte count. Leukocyte count was the only factor among them that was not found to be relevant in any study. From studies concluding that blood protein levels were relevant, 86.67% included only albumin. Diabetes mellitus, congestive heart failure, cancer, chronic obstructive pulmonary disease (COPD), and neutropenia were the relevant associated comorbidities, all for beta-lactams except COPD, which was associated with vancomycin. The only two diagnoses associated with different outcomes were acute respiratory distress syndrome in one study and neurocritical in another. Midazolam was the determining comedication for daptomycin, and drug interactions with quinolones were significant. Types of infections showing differences were abdominal focus sepsis for aminoglycosides and beta-lactams, the Pitt bacteremia score for beta-lactams, and respiratory infections for linezolid and beta-lactams (one study for each). The details of the relevant risk factors that include more different variables can be seen in Figure 2 and Figure 3.

Other risk factors associated with inadequate exposure (evaluated in only one study) were lower protein binding beta-lactams, CYP1A2 polymorphism for ciprofloxacin, heart failure, McCabe score, infusion duration, and high drainage fluid production for beta-lactams. There were also other risk factors related to relevant pharmacokinetic changes: serum sodium, brain glucose concentration, uric acid, cardiac index, and ICU-onset infection. The review identified several other variables that, despite being analyzed, did not significantly impact achieving adequate antimicrobial concentrations or the pharmacokinetic models developed. A comprehensive list of these variables is available in the Supplementary Materials (File S4).

**Figure 4:**
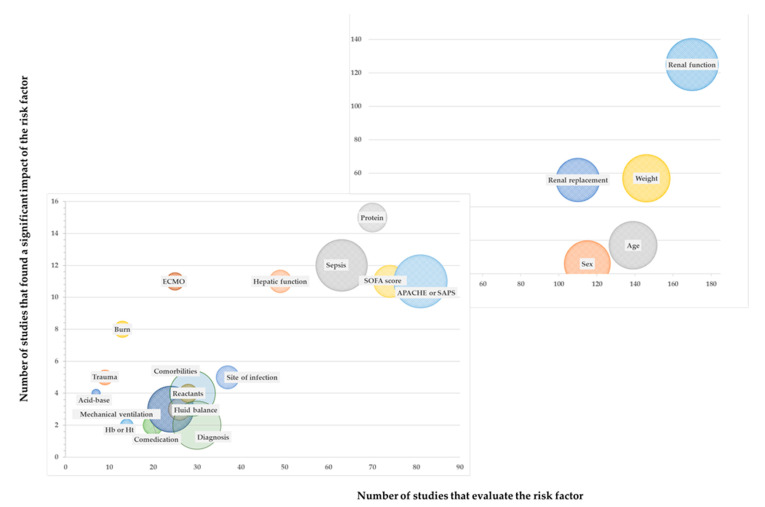
Risk factors with different relevance versus those analyzed, represented on two different scales. The size of the ball indicates the number of patients analyzed. ECMO: Extracorporeal Membrane Oxygenation; Hb: Hemoglobin; Ht Hematocrit.

Some risk factors have been studied much more extensively than others. The relationship between the number of publications that analyzed a risk factor (and the number of patients) versus the number of publications that concluded that risk factors were determinants can be observed in Figure 4.

## 3. Discussion

To the best of our knowledge, this is the first systematic review to identify risk factors in critically ill patients that may compromise the efficacy and safety of antibiotic treatments which included more than just population kinetics. Recently, a very comprehensive systematic review was published, but it included only studies developing population kinetics and focused solely on beta-lactams [54]. Population kinetics can be very helpful in determining the variability of exposure to a treatment, but what we are really interested in is whether these changes will have a clinical impact. Unfortunately, we found very few studies that directly link risk factors to antimicrobial underexposure and clinical outcomes. However, considering the strong correlation between appropriate antibiotic exposure and clinical outcomes [55], analyzing clinical and demographic variables that might influence antibiotic exposure is highly relevant. Various scientific societies and international consensus groups have emphasized the importance of PK/PD objectives in improving clinical outcomes for critically ill patients [10]. Currently, we have information about the desired therapeutic targets, and these have indeed been directly related to significant clinical variables [8,9,10,11,12,13,14]. Including other antibiotics has also helped identify risk factors that may be relevant to therapeutic groups other than beta-lactams, such as liver function in the case of linezolid.

Numerous narrative reviews have explored optimizing antimicrobial dosing in critically ill patients, emphasizing strategies such as extended infusions and higher dosages based on variations in drug clearance and volume of distribution [2,5]. Our review seeks to advance this discussion by identifying less commonly considered parameters that may also influence antimicrobial concentrations in this patient group. We analyzed all the risk factors examined in the studies and highlighted the most significant ones. Our approach not only considers the number of studies that reviewed each factor but also the percentage of studies that found each factor relevant. This method helps us discern which factors have been extensively studied and which have not, particularly when statistical significance is challenging to achieve due to the limited number of studies or small patient populations.

While previous reviews on pharmacokinetics in critically ill patients have predominantly addressed differences from a theoretical perspective—focusing on variations in the volume of distribution, renal function, antimicrobial penetration challenges, and the lipophilicity of these drugs [56], our review takes a more practical approach. We evaluate multiple pharmacokinetic models specifically developed for critically ill patients, allowing us to group and quantify the significance of several less commonly discussed variables in antimicrobial pharmacokinetics.

We concur with the recently published population pharmacokinetics review of beta-lactams [54] that renal function, weight, and renal replacement have the greatest impact on antibiotic exposure, in that order. While it seems clear that these three risk factors affect antibiotic exposure in critically ill patients, dosing regimens used in these settings for most antibiotics are not weight-adjusted. With the increasing prevalence of obesity in both the general population and among patients in critical care units, weight and body surface area have become increasingly important factors in justifying antimicrobial monitoring in critically ill patients. Critically ill obese patients may require higher-than-standard doses of β-lactams, linezolid, and quinolones [57]. Conversely, dosing should be based on total body weight for certain antibiotics such as amikacin, vancomycin, or daptomycin, while adjusted body weight should be used for others. Given the limited availability of pharmacokinetic studies in this patient population and the significant variability in pharmacokinetics among critically ill patients, therapeutic drug monitoring of all administered antibiotics, when possible, is highly recommended.

Regarding beta-lactam drugs, the therapeutic group most commonly used in critically ill patients, our review identified multiple variables associated with variations in plasma concentrations, with weight, renal function, renal replacement therapy (RRT), and age being the most significant. The DALI study [38] revealed that with the doses typically used, about 26% of patients did not reach the minimum target concentration of fT > 50%, and nearly 40% of patients did not achieve fT > 100%. This study also correlated these variables with the clinical outcomes of the patients, highlighting the importance of considering different patient covariates to achieve adequate plasma concentrations. In a narrative review, Stašek J et al. [12] explained the main factors associated with variations in beta-lactam antibiotics in critically ill patients, focusing on renal function, inflammation, hypoalbuminemia, and renal replacement therapy. Our study complements the information provided by other authors, adding data to the number of patients and published articles that consider each variable and introducing new variables such as sex, acid–base disorders, diagnosis at admission, and the site of infections.

The information obtained on vancomycin was the second most abundant in terms of the number of studies, following beta-lactams. Compared to previous reviews [58], which primarily focused on age, weight, and renal function, our study has identified additional variables, such as severity scales, trauma diagnosis on admission, and hypoalbuminemia, as potential factors to consider for optimizing the dose of this drug.

Although there are recommendations for different renal functions and RRT for most antibiotics, the literature often lacks consensus on the most appropriate doses. Additionally, crucial characteristics such as the type and dosage of RRT, which have been shown to be relevant in several studies, are often not considered. The other risk factors described as most relevant in the beta-lactam review and in our work are also similar. Some of these include age, serum albumin, and disease severity, and in no case is dose adjustment considered for these patients.

Both this work and the analyzed studies have limitations. The recommended methodology has been used, and the articles and the document with all the data have been reviewed by several people, but even so, the entire process remains very manual. As a result, we found significant heterogeneity in published population pharmacokinetic studies, which limited the feasibility of meaningfully pooling quantitative parameters. Critically ill patients is a term that includes a clinical heterogeneity of situations across eligible studies that enrolled different populations. We included studies that involved critically ill patients who underwent antimicrobial drug monitoring. However, it is unclear whether all patients had confirmed infections or were necessarily septic. The inclusion of patients who were not septic would increase the likelihood of finding no variables associated with subtherapeutic antibiotic concentrations. Additionally, although we have analyzed studies of all antibiotics without exclusion, a large number of studies are on beta-lactams. Moreover, beta-lactams are a very heterogeneous group of antibiotics, and it would be more appropriate to assess the risk factors affecting each of them separately. The studies that examine exposure do so at the described doses, which may differ from those used in each center. To be able to extrapolate the results from such a large number of articles, we had to combine studies that examine specific subgroups with studies that analyzed risk factors individually, which were not usually the main objective. Studies that reviewed risk factors as a secondary objective did not take this objective into account when calculating the sample size to determine statistical significance. We found that some risk factors are very under-studied, and some are studied in very few patients. There is a lack of studies in routine clinical practice that consider all associated factors and have a sufficient sample size to reach valuable conclusions. Based on the authors’ linguistic expertise, only studies published in English and Spanish were included, which may have led to the exclusion of some studies that could have identified additional variables influencing the pharmacokinetics of antimicrobials in critically ill patients. However, the large number of studies incorporated ensures that the primary variables have been thoroughly considered.

Therefore, we align with the conclusions of many of the analyzed studies: variability in critically ill patients is very high, and the best way to ensure therapeutic target attainment in these patients is to perform Therapeutic Drug Monitoring (TDM). ICU patients are also highly variable among themselves, due to patient characteristics, the reason for admission, the need for vasoactive drugs or fluid replacement, comorbidities, concomitant treatments, lab abnormalities, or the need for extracorporeal supports, among other factors. The effect of the analyzed risk factors can be crucial in determining which patients, among the critically ill, may be at even greater risk of incorrect dosing, and this can be a criterion for prioritizing TDM if it cannot be performed in all patients.

## 4. Materials and Methods

This systematic review was prospectively registered in the International Prospective Register of Systematic Reviews (PROSPERO), identification code CRD42024570977. The protocol adhered to the Preferred Reporting Items for Systematic Reviews and Meta-Analyses (PRISMA) guidelines [59]. 

In order to achieve the objective of this systematic review and identify risk factors among critically ill patients, we conducted a search aimed at finding studies evaluating the effect of risk factors that could significantly influence antibiotic treatments in the ICU. 

We searched for all published studies involving adult patients admitted to the ICU and receiving antibiotic treatment, investigating potential risk factors that could affect antibiotic exposure in these patients. Any variable examined in the included studies for its effect on plasma concentrations of antimicrobials was considered as a risk factor. The details of the search strategy can be found in the Supplementary Materials.

All studies meeting these criteria were included, regardless of whether they focused on these factors as primary objectives or not, analyzed risk factors or directly assessed a population subgroup (e.g., obese patients, those undergoing continuous RRT, ECMO, etc.), studied antibiotic exposure (typically through plasma concentrations), or examined the impact on pharmacokinetic parameters. We categorized covariates using a preplanned, custom classification based on patterns commonly identified in a preliminary literature review conducted by the authors. Given the nature of this review, no formal sensitivity or subgroup analyses were prespecified to assess heterogenicity. Studies not in English or Spanish, retrospective studies, reviews, and abstracts were excluded. Other exclusion criteria were studies that did not assess pharmacokinetic variability or exposure, those that exclusively focused on non-antibiotic agents such as antivirals or antifungals, and those for which full-text access was unavailable.

The search was conducted on 21 March 2024 on the following bibliographic databases: PubMed, Web of Science, and Embase, according to the eligibility criteria and with no time restrictions. The data from searches in each database were exported to an Excel document, and an initial phase for the detection of duplicates was performed. A subsequent duplication detection phase was conducted in the resulting database using a DOI identifier and manual assessment. The results of the refined database were screened (title/abstract) by two independent investigators, with disagreements resolved by a third researcher. This method was replicated for the following full-text assessment and final inclusion of articles. Once selected, data were collected and validated by two independent investigators for each report. Finally, all data were processed in the Excel document, including the most important characteristics of each study.

We assessed the risk of bias in all included randomized clinical trials using Risk of Bias 2 (RoB2). Each study report was assessed by two authors independently, with any disagreements resolved by a third author. For clinical pharmacokinetics studies, the 24-item ClinPK checklist from the Reporting Guidelines for Clinical Pharmacokinetic Studies [60] was used to evaluate the quality of the manuscripts.

## 5. Conclusions

This review allows us to identify which critically ill patients are at a higher risk of not reaching therapeutic targets. This review also helps us recognize the extensive number of variables that have been studied, guiding their inclusion in future studies. It is essential to continue researching, especially in real clinical practice and with clinical outcomes.

## Figures and Tables

**Table 1:** Characteristics of the studies.

		Number of Studies	Number of Patients
**Number of Centers**	Unicentric	222	11,342
	Multicentric	34	10,157
**Study Design**	Prospective observational	246	21,089
	RCT	10	411
**Outcome type**	Exposure	113	15,702
	PKPOP	101	4628
	PK	42	1170
**Type of analysis**	Subgroup	102	4827
	Subgroup and PKPOP	62	2093
	Risk factor, no PKPOP	53	12,045
	Risk factor and PKPOP	39	2535
**Distribution of studies by area**	Europe	125	7036
	North America	39	9303
	East Asia	39	2707
	Oceania	21	700
	South Asia	9	335
	South America	8	689
	Africa	5	187
	More than one area	5	493
	Middle East	1	43
**Publication year**	1988–1996 (9 years)	5	117
	1997–2005 (9 years)	16	445
	2006–2014 (9 years)	43	1997
	2015–2024 (9.2 years)	192	18,941
**Antibiotic group evaluated**	More than two antibiotic groups	6	256
	Aminoglycosides	20	1767
	Antituberculous	1	10
	Beta lactams	137	7395
	Daptomycin	4	101
	Linezolid	23	643
	Polimyxins	7	207
	Quinolones	11	258
	Teicoplanin	7	416
	Tigecycline	4	89
	Vancomycin	42	10,602

PK: Refers to studies that have investigated how risk factors affect pharmacokinetic variables; PKPOP: Refers to studies that have investigated the impact of risk factors as covariates in a population model.

**Table 2:** Number and Percentage of Studies that Concluded a Risk Factor as a Determinant, and the number and percentage that Analyzed Each Risk factor, Classified by Antibiotic Group and Outcome.

	Antibiotic Group											Total
Risk Factor/Primary Outcome	Amino-Glycosides	B-Lactams ^2^	B-Lactams Vancomycin	Daptomycin	Linezolid ^2^	Polymyxins	Quinolone	Teicoplanin	Tigecycline	Vancomycin	Other ^1^	Number of Studies
**Age**	**1/10 (10.0)**	9/80 (11.3)	**0/1 (0)**	**0/2 (0)**	**0/11 (0)**	**1/4 (25.0)**	**2/9 (22.2)**	**1/4 (25.0)**	**0/3(0)**	**3/16 (18.8)**	**0**	**17/139 (12.2)**
Exposure	1/5 (20.0)	3/20 (15.0)	0/1 (0)	0/1 (0)	0/1 (0)	1/3 (33.3)	1/2 (50.0)	1/2(50.0)	0	1/6 (16.7)	0	8/41 (19.5)
PK/PKPOP	0/5 (0)	6/60 (10.0)	0	0/1 (0)	0/10 (0)	0/1 (0)	1/7 (14.3)	0/2 (0)	0/3(0)	2/10 (20.0)	0	9/98 (9.2)
**Sex**	**0/8 (0)**	**4/68 (5.9)**	**0**	**0/2 (0)**	**0/8 (0)**	**0/3 (0)**	**2/6 (33.3)**	**0/3 (0)**	**0/3 (0)**	**0/15 (0)**	**0**	**6/115 (5.2)**
Exposure	0/4 (0)	1/15 (6.7)	0	0/1 (0)	0	0/2 (0)	2/2 (100)	0/1 (0)	0	0/6 (0)	0	3/31 (9.7)
PK/PKPOP	0/4 (0)	3/53 (5.7)	0	0/1 (0)	0/8 (0)	0/1 (0)	0/4 (0)	0/2 (0)	0/3 (0)	0/9 (0)	0	3/84 (3.6)
**Weight**	**5/7 (71.4)**	**30/88 (34.1)**	**0/1**	**0/2 (0)**	**6/12 (50.0)**	**2/6 (33.3)**	**4/8 (50.0)**	**1/3 (33.3)**	**2/3 (66.7)**	**8/17 (47.1)**	**0**	**57/146 (39.0)**
Exposure	1/2 (50.0)	6/22 (27.3)	0/1	0/1 (0)	1/2 (50.0)	2/4 (50.0)	1/2 (50.0)	0/1 (0)	0	3/5 (60.0)	0	14/40 (35.0)
PK/PKPOP	4/5 (80.0)	24/66 (36.4)	0	0/1 (0)	5/10 (50.0)	0/2 (0)	3/6 (50.0)	1/2 (50.0)	2/3 (66.7)	5/12 (41.7)	0	43/106 (40.6)
**Renal function**	**8/11 (72.7)**	**74/98 (75.5)**	**2/2 (100)**	**1/2 (50.0)**	**8/13 (61.5)**	**3/5 (60.0)**	**6/8 (75.0)**	**3/4 (75.0)**	**0/2 (0)**	**18/24 (75.0)**	**2/2 (100)**	**125/170 (73.5)**
Exposure	4/5 (80.0)	24/30 (80.0)	2/2 (100)	0/1 (0)	1/2 (50.0)	2/3 (66.7)	2/3 (66.7)	1/1 (100)	0	10/12 (83.3)	2/2 (100)	48/61 (78.7)
PK/PKPOP	4/6 (66.7)	50/68 (73.5)	0	1/1 (100)	7/11 (63.6)	1/2 (50.0)	4/5 (80.0)	2/3 (66.7)	0/2 (0)	8/12 (66.7)	0	77/109 (70.6)
**Renal replacement**	**3/7 (42.9)**	**22/50 (44.0)**	**2/3 (66.7)**	**3/3 (100)**	**5/14 (35.7)**	**2/2 (100)**	**0/5 (0)**	**3/5 (60.0)**	**1/3 (33.3)**	**13/17 (76.5)**	**3/3 (100)**	**57/110 (51.8)**
Exposure	1/4 (25.0)	9/21 (42.9)	1/2 (50.0)	1/1 (100)	2/4 (50.0)	1/1 (100)	0	2/2 (100)	1/1 (100)	7/9 (77.8)	1/1 (100)	26/45 (57.8)
PK/PKPOP	2/3 (66.7)	13/29 (44.8)	1/1 (100)	2/2 (100)	3/10 (30.0)	1/1 (100)	0/5 (0)	1/3 (33.3)	0/2 (0)	6/8 (75.0)	2/2 (100)	31/65 (47.7)
**Protein or albumin**	**1/4 (25.0)**	**10/49 (20.4)**	**0/1 (0)**	**0/1 (0)**	**0/2 (0)**	**1/4 (25.0)**	**0/1 (0)**	**1/2 (50.0)**	**0/2 (0)**	**2/4 (50.0)**	**0**	**15/70 (21.4)**
Exposure	0/2 (0)	1/10 (10.0)	0/1 (0)	0	0	1/2 (50.0)	0	1/1 (100)	0	1/1 (100)	0	4/17 (23.5)
PK/PKPOP	1/2 (50.0)	9/39 (23.1)	0	0/1 (0)	0/2 (0)	0/2 (0)	0/1 (0)	0/1 (0)	0/2 (0)	1/3 (33.3)	0	11/53 (20.8)
**APACHE or SAPS**	**2/6 (33.3)**	**5/46 (10.9)**	**0/1 (0)**	**0/1 (0)**	**0/6 (0)**	**0/2 (0)**	**1/3 (33.3)**	**1/2 (50.0)**	**0/1 (0)**	**1/11 (9.1)**	**1/2 (50.0)**	**11/81 (13.6)**
Exposure	2/4 (50.0)	3/14 (21.4)	0/1 (0)	0	0/1 (0)	0/1 (0)	0	1/1 (100)	0	1/6 (16.7)	1/2 (50.0)	8/30 (26.7)
PK/PKPOP	0/2 (0)	2/32 (6.3)	0	0/1 (0)	0/5 (0)	0/1 (0)	1/3 (33.3)	0/1 (0)	0/1 (0)	0/5 (0)	0	3/51 (5.9)
**SOFA score**	**2/5 (40.0)**	**5/46 (10.9)**	**1/1 (100)**	**0**	**1/5 (20.0)**	**0/2 (0)**	**1/3 (33.3)**	**0**	**0/1 (0)**	**1/10 (10.0)**	**0/1 (0)**	**11/74 (14.9)**
Exposure	2/3 (66.7)	4/13 (30.8)	1/1 (100)	0	0	0/2 (0)	1/1 (100)	0	0	0/4 (0)	0/1 (0)	8/25 (32.0)
PK/PKPOP	0/2 (0)	1/33 (3.0)	0	0	1/5 (20.0)	0	0/2 (0)	0	0/1 (0)	1/6 (16.7)	0	3/49 (6.1)
**Hepatic function**	**1/3 (33.3)**	**1/23 (4.3)**	**0**	**0/1 (0)**	**5/8 (62.5)**	**0/3 (0)**	**2/6 (33.3)**	**0**	**1/1 (100)**	**0/3 (0)**	**1/1 (100)**	**11/49 (22.4)**
Exposure	1/2 (50.0)	1/3 (33.3)	0	0	0	0/2 (0)	0/2 (0)	0	0	0/1 (0)	1/1 (100)	3/11 (27.3)
PK/PKPOP	0/1 (0)	0/20 (0)	0	0/1 (0)	5/8 (62.5)	0/1 (0)	2/4 (50.0)	0	1/1 (100)	0/2 (0)	0	8/38 (21.1)
**Sepsis/shock**	**1/6 (16.7)**	**7/39 (17.9)**	**0**	**0**	**1/2 (50.0)**	**0**	**0/3 (0)**	**0**	**0**	**2/11 (18.2)**	**1/2 (50.0)**	**12/63 (19.0)**
Exposure	0/3 (0)	3/13 (23.1)	0	0	1/1 (100)	0	0/1 (0)	0	0	2/7 (28.6)	1/2 (50.0)	7/27 (25.9)
PK/PKPOP	1/3 (33.3)	4/26 (15.4)	0	0	0/1 (0)	0	0/2 (0)	0	0	0/4 (0)	0	5/36 (13.9)
**Admission** **Diagnosis**	**0/1 (0)**	**1/17 (5.9)**	**0/1 (0)**	**0/1 (0)**	**1/2 (50.0)**	**0**	**0**	0/1 (0)	**0**	**0/7 (0)**	**0**	**2/30 (6.7)**
Exposure	0/1 (0)	1/6 (16.7)	0/1 (0)	0/1 (0)	0/1 (0)	0	0	0/1 (0)	0	0/4 (0)	0	1/15 (6.7)
PK/PKPOP	0	0/11 (0)	0	0	1/1 (100)	0	0	0	0	0/3 (0)	0	1/15 (6.7)
**Trauma**	**1/1 (100)**	**3/5 (60.0)**	**0**	**0**	**0**	**0**	**0**	**0**	**0**	**1/3 (33.3)**	**0**	**5/9 (55.6)**
Exposure	1/1 (100)	0/2 (0)	0	0	0	0	0	0	0	1/3 (33.3)	0	2/6 (33.3)
PK/PKPOP	0	3/3 (100)	0	0	0	0	0	0	0	0	0	3/3 (100)
**Burn**	**2/2 (100)**	**6/9 (66.7)**	**0**	**0**	**0/1 (0)**	**0**	**0**	**0**	**0**	**0/1 (0)**	**0**	**8/13 (61.5)**
Exposure	1/1 (100)	5/6 (83.3)	0	0	0/1 (0)	0	0	0	0	0	0	6/8 (75.0)
PK/PKPOP	1/1 (100)	1/3 (33.3)	0	0	0	0	0	0	0	0/1 (0)	0	2/5 (40.0)
**ECMO**	**1/2 (50.0)**	**6/14 (42.9)**	**0**	**0/1 (0)**	**2/2 (100)**	**0**	**0/2 (0)**	**0**	**0/1 (0)**	**1/2 (50.0)**	**2/2 (100)**	**11/25 (44.0)**
Exposure	0/1 (0)	1/5 (20.0)	0	0/1 (0)	2/2 (100)	0	0	0	0	1/1 (100)	2/2 (100)	5/11 (45.5)
PK/PKPOP	1/1 (100)	5/9 (55.6)	0	0	0	0	0/2 (0)	0	0/1 (0)	0/1 (0)	0	6/14 (42.9)
**Mechanical** **ventilation**	**0/4 (0)**	**1/14 (7.1)**	**0**	**0**	**0**	**0**	**1/1 (100)**	**0**	**0**	**1/5 (20.0)**	**0**	**3/24 (12.5)**
Exposure	0/3 (0)	0/4 (0)	0	0	0	0	0	0	0	1/4 (25.0)	0	1/11 (9.1)
PK/PKPOP	0/1 (0)	1/10 (10.0)	0	0	0	0	1/1 (100)	0	0	0/1 (0)	0	2/13 (15.4)
**pH parameters**	**0**	**1/3 (33.3)**	**0**	**0**	**1/1 (100)**	**0**	**1/1 (100)**	**0/1 (0)**	**0**	**0**	**1/1 (100)**	**4/7 (57.1)**
Exposure	0	0	0	0	0	0	0	0/1 (0)	0	0	1/1 (100)	1/2 (50.0)
PK/PKPOP	0	1/3 (33.3)	0	0	1/1 (100)	0	1/1 (100)	0	0	0	0	3/5 (60.0)
**Acute reactants**	**1/3 (33.3)**	**3/19 (15.8)**	**0**	**0/1 (0)**	**0/1 (0)**	**0**	**0**	**0/1 (0)**	**0/1 (0)**	**0/2 (0)**	**0**	**4/28 (14.3)**
Exposure	1/2 (50.0)	1/4 (25.0)	0	0	0	0	0	0/1 (0)	0	0/1 (0)	0	2/8 (25.0)
PK/PKPOP	0/1 (0)	2/15 (13.3)	0	0/1 (0)	0/1 (0)	0	0	0	0/1 (0)	0/1 (0)	0	2/20 (10.0)
**Hemoglobin/hematocrit**	**0/2 (0)**	**1/7 (14.3)**	**0**	**0/2 (0)**	**0/1 (0)**	**1/1 (100)**	**0**	**0**	**0**	**0/1 (0)**	**0**	**2/14 (14.3)**
Exposure	0/1 (0)	0/1 (0)	0	0/1 (0)	0	0	0	0	0	0	0	0/3 (0)
PK/PKPOP	0/1 (0)	1/6 (16.7)	0	0/1 (0)	0/1 (0)	1/1 (100)	0	0	0	0/1 (0)	0	2/11 (18.2)
**Fluid balance**	**1/4 (25.0)**	**2/15 (13.3)**	**0/1 (0)**	**0**	**0**	**0**	**0/2 (0)**	**0**	**0/2 (0)**	**0/2 (0)**	**0**	**3/26 (11.5)**
Exposure	0/3 (0)	1/3 (33.3)	0/1 (0)	0	0	0	0/1 (0)	0	0	0/1 (0)	0	1/9 (11.1)
PK/PKPOP	1/1 (100)	1/12 (8.3)	0	0	0	0	0/1 (0)	0	0/2 (0)	0/1 (0)	0	2/17 (11.8)
**Comorbidities**	**0/4 (0)**	**3/14 (21.4)**	**0**	**0/1 (0)**	**0/1 (0)**	**0/1 (0)**	**0/2 (0)**	**0**	**0**	**1/6 (16.7)**	**0/1 (0)**	**4/29 (13.8)**
Exposure	0/4 (0)	1/6 (16.7)	0	0/1 (0)	0	0/1 (0)	0	0	0	1/2 (50.0)	0/1 (0)	2/15 (13.3)
PK/PKPOP	0	2/8 (25.0)	0	0	0/1 (0)	0	0/2 (0)	0	0	0/4 (0)	0	2/14 (14.3)
**Comedication**	**0/1 (0)**	**0/10 (0)**	**0**	**1/1 (100)**	**0**	**0/2 (0)**	**1/2 (50.0)**	**0**	**0**	**0/3 (0)**	**0/1 (0)**	**2/20 (10.0)**
Exposure	0/1 (0)	0/7 (0)	0	1/1 (100)	0	0/1 (0)	0	0	0	0/1 (0)	0/1 (0)	1/12 (8.3)
PK/PKPOP	0	0/3 (0)	0	0	0	0/1 (0)	1/2 (50.0)	0	0	0/2 (0)	0	1/8 (12.5)
**Site of infection**	**1/3 (33.3)**	**3/21 (14.3)**	**0**	**0/1 (0)**	**1/4 (25.0)**	**0/2 (0)**	**0/2 (0)**	**0/1 (0)**	**0**	**0/3 (0)**	**0**	**5/37 (13.5)**
Exposure	0/1 (0)	3/9 (33.3)	0	0/1 (0)	1/2 (50.0)	0/1 (0)	0/1 (0)	0/1 (0)	0	0/1 (0)	0	4/17 (23.5)
PK/PKPOP	1/2 (50.0)	0/12 (0)	0	0	0/2 (0)	0/1 (0)	0/1 (0)	0	0	0/2 (0)	0	1/20 (5.0)

ECMO: Extracorporeal membrane oxygenation. PK: Studies that have investigated how risk factors affect pharmacokinetic variables; PKPOP: Studies that have investigated the impact of risk factors as covariates in a population model. ^1^ Other includes studies with more than two groups of antibiotics and one study on antitubercular agents. ^2^ A study that evaluated linezolid and beta-lactams together, this has been counted as two studies.

## Data Availability

Data are available under reasonable request.

## Supplementary Materials

The following supporting information can be downloaded at: https://www.mdpi.com/article/10.3390/antibiotics13090801/s1, File S1: Search strategy; File S2: Excluded and Included studies; File S3: Risk of bias analysis; File S4: Risk factors that were studied without finding any impact; File S5: Main characteristics of studies. References [28,32,33,34,36,49,61,62,63,64,65,66,67,68,69,70,71,72,73,74,75,76,77,78,79,80,81,82,83,84,85,86,87,88,89,90,91,92,93,94,95,96,97,98,99,100,101,102,103,104,105,106,107,108,109,110,111,112,113,114,115,116,117,118,119,120,121,122,123,124,125,126,127,128,129,130,131,132,133,134,135,136,137,138,139,140,141,142,143,144,145,146,147,148,149,150,151,152,153,154,155,156,157,158,159,160,161,162,163,164,165,166,167,168,169,170,171,172,173,174,175,176,177,178,179,180,181,182,183,184,185,186,187,188,189,190,191,192,193,194,195,196,197,198,199,200,201,202,203,204,205,206,207,208,209,210,211,212,213,214,215,216,217,218,219,220,221,222,223,224,225,226,227,228,229,230,231,232,233,234,235,236,237,238,239,240,241,242,243,244,245,246,247,248,249,250,251,252,253,254,255,256,257,258,259,260,261,262,263,264,265,266,267,268,269,270,271,272,273,274,275,276,277,278,279,280,281,282,283,284,285,286,287,288,289,290,291,292,293,294,295,296,297,298,299,300,301,302,303,304,305,306,307,308,309] are the studies included in the review, that are cited in the Supplementary Materials.
