# Supplementary material for: Risk Factors Associated with Antibiotic Exposure Variability in Critically Ill Patients: A Systematic Review
Source: Antibiotics (Basel). 2024 Aug 24;13(9):801. doi: 10.3390/antibiotics13090801 (PMC11428266; doi:10.3390/antibiotics13090801)
Supplement: Supplementary file 1 [file antibiotics-13-00801-s001.zip › antibiotics-3140388-supplementary.pdf]

Supplementary Materials

Risk Factors Associated with Antibiotic Exposure Variability in Critically Ill Patients: A Systematic Review

Laura Gras-Martín, Adrián Plaza-Diaz, Borja Zarate-Tamames, Paula Vera-Artazcoz, Olga H. Torres, Carla Bastida, Dolors Soy and Jesús Ruiz-Ramos

Correspondence: Jesús Ruiz-Ramos; jrzrms@gmail.com

File S1

Search strategy

To conduct the search, the following keywords were concluded for each section of the PICO question:

- P (PATIENT): Critically ill patients (Critically ill patients OR critical care OR Critical illness OR critically ill OR Intensive care units OR ICU) under antibiotic treatment (Piperacillin OR Piperacillin-tazobactam OR Meropenem OR Linezolid OR Daptomycin OR imipenem OR cefepime OR Beta-lactam OR Beta-lactams OR Fluoroquinolones OR Vancomycin OR Aminoglycosides OR Anti-Bacterial Agents OR antibacterial agents OR Antibacterial OR Anti-Infective Agents OR antibiotic OR antibiotics OR bacterial infections).
- I (INTERVENTION): Described alterations of pharmacokinetic parameters (Target attainment OR Breakpoint of target attainment OR drug concentration OR subtherapeutic OR supratherapeutic OR toxicity OR efficacy OR pharmacokinetics OR pharmacodynamics OR TDM OR Therapeutic drug monitoring Precision dosing OR population pharmacokinetic)
- C (COMPARATOR): Rest of the population admitted to the ICU.
- O (OUTCOME): Variations in pharmacokinetic parameters or subtherapeutic or supratherapeutic plasma concentrations of antibiotics.

Taking into account that we only wanted to include studies with humans, adult patients, and exclude retrospective articles and reviews, the final search was conducted with the following text:

(Critically ill patients OR critical care OR Critical illness OR critically ill OR Intensive care units OR ICU) AND (Piperacillin OR Piperacillin-tazobactam OR Meropenem OR Linezolid OR Daptomycin OR imipenem OR cefepime OR Beta-lactam OR Beta-lactams OR Fluoroquinolones OR Vancomycin OR Aminoglycosides OR Anti-Bacterial Agents OR antibacterial agents OR Antibacterial OR Anti-Infective Agents OR antibiotic OR antibiotics OR bacterial infections) AND (Target attainment OR Breakpoint of target attainment OR drug concentration OR subtherapeutic OR supratherapeutic OR toxicity OR efficacy OR pharmacokinetics OR pharmacodynamics OR TDM OR Therapeutic drug monitoring Precision dosing OR population pharmacokinetic) AND (prospective) NOT (Systematic review OR Meta-analysis OR Review OR Study Protocol) NOT (Pediatric OR Neonates OR Infants OR Children)

File S2

Excluded and Included studies

Excluded for Not accesss

- Georges B, Conil JM, Seguin T, Dieye E, Cougot P, Decun JF, Lavit M, Samii K, Houin G, Saivin S. Cefepime in intensive care unit patients: validation of a population pharmacokinetic approach and influence of covariables. Int J Clin Pharmacol Ther. 2008 Apr;46(4):157-64.
- Bang JY, Kang HI, Lee HJ, Chong YP, Hong SK, Lee EK, Choi BM, Noh GJ. Development of a new pharmacokinetic model for target-concentration controlled infusion of vancomycin in critically ill patients. Clin Exp Pharmacol Physiol. 2022 Feb;49(2):202-211.
- Barreto EF, Chang J, Bjergum MW, Gajic O, Jannetto PJ, Mara KC, Meade LA, Rule AD, Vollmer KJ, Scheetz MH; BLOOM Study Group. Adequacy of cefepime concentrations in the early phase of critical illness: A case for precision pharmacotherapy. Pharmacotherapy. 2023 Nov;43(11):1112-1120.
- Ngougni Pokem P, Liu X, Parker SL, Verroken A, Collienne C, Finet P, Wijnant GJ, Laterre PF, Roberts JA, Van Bambeke F, Wittebole X. Population pharmacokinetics and dosing simulations of total and unbound temocillin in the plasma and CSF of neurocritically ill patients with external ventricular drain-related cerebral ventriculitis. J Antimicrob Chemother. 2024 Feb 1;79(2):429-442.
- Polard E, Le Bouquin V, Le Corre P, Kérebel C, Trout H, Feuillu A, Le Verge R, Mallédant Y. Non steady state and steady state PKS Bayesian forecasting and vancomycin pharmacokinetics in ICU adult patients. Ther Drug Monit. 1999 Aug;21(4):395-403.
- Zhao HH, Tang WJ, Yang YX, Cen ZR, Wang LQ. PK/PD study of tigecycline in severely infected patients with continuous renal replacement therapy. Int J Clin Pharmacol Ther. 2020 Oct;58(10):531-538.
- Sridharan K, Pasha SAA, Qader AM, Hasan HMSN, El Seirafi MM. Drug Utilization in Critically Ill Adults with Augmented Renal Clearance Compared to Normal Renal Clearance: Implications for use of Antimicrobials with Predominant Renal Excretion. Curr Rev Clin Exp Pharmacol. 2021;16(2):174-181.

Excluded for retrospective studies

- Maranchick NF, Trillo-Alvarez C, Kariyawasam V, Venugopalan V, Kwara A, Rand K, Peloquin CA, Alshaer MH. A Randomized Clinical Trial of Bayesian-Guided Beta-Lactam Infusion Strategy and Associated Bacterial Resistance and Clinical Outcomes in Patients With Severe Pneumonia. Ther Drug Monit. 2024 Feb 1;46(1):95-101.
- Frazee EN, Kuper PJ, Schramm GE, Larson SL, Kashani KB, Osmon DR, Leung N. Effect of continuous venovenous hemofiltration dose on achievement of adequate vancomycin trough concentrations. Antimicrob Agents Chemother. 2012 Dec;56(12):6181-5.
- Damen C, Dhaese S, Verstraete AG, Stove V, De Waele JJ. Subtherapeutic piperacillin concentrations in neurocritical patients. J Crit Care. 2019 Dec;54:48-51.
- Gatti M, Rinaldi M, Laici C, Siniscalchi A, Viale P, Pea F. Role of a Real-Time TDM-Based Expert Clinical Pharmacological Advice Program in Optimizing the Early Pharmacokinetic/Pharmacodynamic Target Attainment of Continuous Infusion Beta-Lactams among Orthotopic Liver Transplant Recipients with Documented or Suspected Gram-Negative Infections. Antibiotics (Basel). 2023 Nov 7;12(11):1599.
- Fan G, Jin L, Bai H, Jiang K, Xie J, Dong Y. Safety and Efficacy of Tigecycline in Intensive Care Unit Patients Based on Therapeutic Drug Monitoring. Ther Drug Monit. 2020 Dec;42(6):835-840.
- Gatti M, Rinaldi M, Tonetti T, Siniscalchi A, Viale P, Pea F. Could an Optimized Joint Pharmacokinetic/Pharmacodynamic Target Attainment of Continuous Infusion Piperacillin-Tazobactam Be a Valuable Innovative Approach for Maximizing the Effectiveness of Monotherapy Even in the Treatment of Critically Ill Patients with Documented Extended-Spectrum Beta-

Lactamase-Producing Enterobacterales Bloodstream Infections and/or Ventilator-Associated Pneumonia? Antibiotics (Basel). 2023 Dec 14;12(12):1736.

- Liebchen U, Klose M, Paal M, Vogeser M, Zoller M, Schroeder I, Schmitt L, Huisinga W, Michelet R, Zander J, Scharf C, Weinelt FA, Kloft C. Evaluation of the MeroRisk Calculator, A User-Friendly Tool to Predict the Risk of Meropenem Target Non-Attainment in Critically Ill Patients. Antibiotics (Basel). 2021 Apr 20;10(4):468.
- Donadello K, Roberts JA, Cristallini S, Beumier M, Shekar K, Jacobs F, Belhaj A, Vincent JL, de Backer D, Taccone FS. Vancomycin population pharmacokinetics during extracorporeal membrane oxygenation therapy: a matched cohort study. Crit Care. 2014 Nov 22;18(6):632.
- Zheng L, Alshaer MH, Peloquin C, Venugopalan V, Alnuaimat HM, Converse M. Cefepime pharmacokinetics in adult extracorporeal membrane oxygenation patients. Pulm Pharmacol Ther. 2024 Mar;84:102271.

Excluded for Review

- Gatti M, Giannella M, Raschi E, Viale P, De Ponti F. Ceftolozane/tazobactam exposure in critically ill patients undergoing continuous renal replacement therapy: a PK/PD approach to tailor dosing. J Antimicrob Chemother. 2021 Jan 1;76(1):199-205.

Excluidos for evalaution of adverse events as unique endpoint.

- Bertino JS Jr, Booker LA, Franck PA, Jenkins PL, Franck KR, Nafziger AN. Incidence of and significant risk factors for aminoglycoside-associated nephrotoxicity in patients dosed by using individualized pharmacokinetic monitoring. J Infect Dis. 1993 Jan;167(1):173-9.
- Kane-Gill SL, Ostermann M, Shi J, Joyce EL, Kellum JA. Evaluating Renal Stress Using Pharmacokinetic **Urinary Biomarker** Data in Critically Ill Patients Receiving Vancomycin and/or Piperacillin-Tazobactam: A Secondary Analysis of the Multicenter Sapphire Study. Drug Saf. 2019 Oct;42(10):1149-1155.

Studies included in the review

1. Whitehouse T, Cepeda JA, Shulman R, Aarons L, Nalda-Molina R, Tobin C, MacGowan A, Shaw S, Kibbler C, Singer M, Wilson AP. Pharmacokinetic studies of linezolid and teicoplanin in the critically ill. J Antimicrob Chemother. 2005 Mar;55(3):333-40.
2. Burkhardt O, Kumar V, Katterwe D, Majcher-Peszynska J, Drewelow B, Derendorf H, Welte T. Ertapenem in critically ill patients with early-onset ventilator-associated pneumonia: pharmacokinetics with special consideration of free-drug concentration. J Antimicrob Chemother. 2007 Feb;59(2):277-84.
3. Roos JF, Lipman J, Kirkpatrick CMJ. Population pharmacokinetics and pharmacodynamics of cefpirome in critically ill patients against Gram-negative bacteria. Intensive Care Med. 2007 May;33(5):781-788.
4. Roberts JA, Field J, Visser A, Whitbread R, Tallot M, Lipman J, Kirkpatrick CM. Using population pharmacokinetics to determine gentamicin dosing during extended daily diafiltration in critically ill patients with acute kidney injury. Antimicrob Agents Chemother. 2010 Sep;54(9):3635-40.
5. Asín-Prieto E, Rodríguez-Gascón A, Trocóniz IF, Soraluce A, Maynar J, Sánchez-Izquierdo JÁ, Isla A. Population pharmacokinetics of piperacillin and tazobactam in critically ill patients undergoing continuous renal replacement therapy: application to pharmacokinetic/pharmacodynamic analysis. J Antimicrob Chemother. 2014 Jan;69(1):180-9.
6. Escobar L, Andresen M, Downey P, Gai MN, Regueira T, Bórquez T, Lipman J, Roberts JA. Population pharmacokinetics and dose simulation of vancomycin in critically ill patients during high-volume haemofiltration. Int J Antimicrob Agents. 2014 Aug;44(2):163-7.

7. Couffignal C, Pajot O, Laouénan C, Burdet C, Foucrier A, Wolff M, Armand-Lefevre L, Mentré F, Massias L. Population pharmacokinetics of imipenem in critically ill patients with suspected ventilator-associated pneumonia and evaluation of dosage regimens. Br J Clin Pharmacol. 2014 Nov;78(5):1022-34.
8. Carlier M, Noë M, Roberts JA, Stove V, Verstraete AG, Lipman J, De Waele JJ. Population pharmacokinetics and dosing simulations of cefuroxime in critically ill patients: non-standard dosing approaches are required to achieve therapeutic exposures. J Antimicrob Chemother. 2014 Oct;69(10):2797-803.
9. Zoller M, Maier B, Hornuss C, Neugebauer C, Döbbeler G, Nagel D, Holdt LM, Bruegel M, Weig T, Grabein B, Frey L, Teupser D, Vogeser M, Zander J. Variability of linezolid concentrations after standard dosing in critically ill patients: a prospective observational study. Crit Care. 2014 Jul 10;18(4):R148.
10. Luque S, Grau S, Alvarez-Lerma F, Ferrández O, Campillo N, Horcajada JP, Basas M, Lipman J, Roberts JA. Plasma and cerebrospinal fluid concentrations of linezolid in neurosurgical critically ill patients with proven or suspected central nervous system infections. Int J Antimicrob Agents. 2014 Nov;44(5):409-15.
11. Ulldemolins M, Soy D, Llaurado-Serra M, Vaquer S, Castro P, Rodríguez AH, Pontes C, Calvo G, Torres A, Martín-Loeches I. Meropenem population pharmacokinetics in critically ill patients with septic shock and continuous renal replacement therapy: influence of residual diuresis on dose requirements. Antimicrob Agents Chemother. 2015 Sep;59(9):5520-8.
12. Kees MG, Minichmayr IK, Moritz S, Beck S, Wicha SG, Kees F, Kloft C, Steinke T. Population pharmacokinetics of meropenem during continuous infusion in surgical ICU patients. J Clin Pharmacol. 2016 Mar;56(3):307-15.
13. Abdul-Aziz MH, Abd Rahman AN, Mat-Nor MB, Sulaiman H, Wallis SC, Lipman J, Roberts JA, Staatz CE. Population Pharmacokinetics of Doripenem in Critically Ill Patients with Sepsis in a Malaysian Intensive Care Unit. Antimicrob Agents Chemother. 2015 Oct 19;60(1):206-14.
14. Roger C, Muller L, Wallis SC, Louart B, Saissi G, Lipman J, Lefrant JY, Roberts JA. Population pharmacokinetics of linezolid in critically ill patients on renal replacement therapy: comparison of equal doses in continuous venovenous haemofiltration and continuous venovenous haemodiafiltration. J Antimicrob Chemother. 2016 Feb;71(2):464-70.
15. Roberts JA, Cotta MO, Cojutti P, Lugano M, Della Rocca G, Pea F. Does Critical Illness Change Levofloxacin Pharmacokinetics? Antimicrob Agents Chemother. 2015 Dec 14;60(3):1459-63.
16. Ulldemolins M, Martín-Loeches I, Llauradó-Serra M, Fernández J, Vaquer S, Rodríguez A, Pontes C, Calvo G, Torres A, Soy D. Piperacillin population pharmacokinetics in critically ill patients with multiple organ dysfunction syndrome receiving continuous venovenous haemodiafiltration: effect of type of dialysis membrane on dosing requirements. J Antimicrob Chemother. 2016 Jun;71(6):1651-9.
17. Roger C, Wallis SC, Louart B, Lefrant JY, Lipman J, Muller L, Roberts JA. Comparison of equal doses of continuous venovenous haemofiltration and haemodiafiltration on ciprofloxacin population pharmacokinetics in critically ill patients. J Antimicrob Chemother. 2016 Jun;71(6):1643-50.
18. Alobaid AS, Wallis SC, Jarrett P, Starr T, Stuart J, Lassig-Smith M, Ordóñez Mejia JL, Roberts MS, Lipman J, Roberts JA. Effect of Obesity on the Population Pharmacokinetics of Meropenem in Critically Ill Patients. Antimicrob Agents Chemother. 2016 Jul 22;60(8):4577-84.
19. Taubert M, Zoller M, Maier B, Frechen S, Scharf C, Holdt LM, Frey L, Vogeser M, Fuhr U, Zander J. Predictors of Inadequate Linezolid Concentrations after Standard Dosing in Critically Ill Patients. Antimicrob Agents Chemother. 2016 Aug 22;60(9):5254-61.
20. Tsai D, Stewart P, Goud R, Gourley S, Hewagama S, Krishnaswamy S, Wallis SC, Lipman J, Roberts JA. Optimising meropenem dosing in critically ill Australian Indigenous patients with severe sepsis. Int J Antimicrob Agents. 2016 Nov;48(5):542-546. doi: 10.1016/j.ijantimicag.2016.08.015.
21. Blassmann U, Roehr AC, Frey OR, Vetter-Kerkhoff C, Thon N, Hope W, Briegel J, Huge V. Cerebrospinal fluid penetration of meropenem in neurocritical care patients with proven or suspected ventriculitis: a prospective observational study. Crit Care. 2016 Oct 24;20(1):343.

22. Rahbar AJ, Lodise TP, Abraham P, Lockwood A, Pai MP, Patka J, Rabinovich M, Curzio K, Chester K, Williams B, Morse B, Chaar M, Huang V, Salomone J. Pharmacokinetic and Pharmacodynamic Evaluation of Doripenem in Critically Ill Trauma Patients with Sepsis. Surg Infect (Larchmt). 2016 Dec;17(6):675-682.
23. Naik BI, Roger C, Ikeda K, Todorovic MS, Wallis SC, Lipman J, Roberts JA. Comparative total and unbound pharmacokinetics of cefazolin administered by bolus versus continuous infusion in patients undergoing major surgery: a randomized controlled trial. Br J Anaesth. 2017 Jun 1;118(6):876-882.
24. Xie J, Roberts JA, Alobaid AS, Roger C, Wang Y, Yang Q, Sun J, Dong H, Wang X, Xing J, Lipman J, Dong Y. Population Pharmacokinetics of Tigecycline in Critically Ill Patients with Severe Infections. Antimicrob Agents Chemother. 2017 Jul 25;61(8):e00345-17.
25. Wicha SG, Frey OR, Roehr AC, Pratschke J, Stockmann M, Alraish R, Wuensch T, Kaffarnik M. Linezolid in liver failure: exploring the value of the maximal liver function capacity (LiMAx) test in a pharmacokinetic pilot study. Int J Antimicrob Agents. 2017 Oct;50(4):557-563.
26. Sime FB, Hahn U, Warner MS, Tiong IS, Roberts MS, Lipman J, Peake SL, Roberts JA. Using Population Pharmacokinetic Modeling and Monte Carlo Simulations To Determine whether Standard Doses of Piperacillin in Piperacillin-Tazobactam Regimens Are Adequate for the Management of Febrile Neutropenia. Antimicrob Agents Chemother. 2017 Oct 24;61(11):e00311-17.
27. Sjövall F, Alobaid AS, Wallis SC, Perner A, Lipman J, Roberts JA. Maximally effective dosing regimens of meropenem in patients with septic shock. J Antimicrob Chemother. 2018 Jan 1;73(1):191-198.
28. Borsuk-De Moor A, Rypulak E, Potręć B, Piwowarczyk P, Borys M, Sysiak J, Onichimowski D, Raszewski G, Czuczwar M, Wiczling P. Population Pharmacokinetics of High-Dose Tigecycline in Patients with Sepsis or Septic Shock. Antimicrob Agents Chemother. 2018 Mar 27;62(4):e02273-17.
29. Braune S, König C, Roberts JA, Nierhaus A, Steinmetz O, Baehr M, Kluge S, Langebrake C. Pharmacokinetics of meropenem in septic patients on sustained low-efficiency dialysis: a population pharmacokinetic study. Crit Care. 2018 Jan 30;22(1):25.
30. Bos JC, Prins JM, Mistício MC, Nunguiane G, Lang CN, Beirão JC, Mathôt RAA, van Hest RM. Pharmacokinetics and pharmacodynamic target attainment of ceftriaxone in adult severely ill sub-Saharan African patients: a population pharmacokinetic modelling study. J Antimicrob Chemother. 2018
31. Hanberg P, Öbrink-Hansen K, Thorsted A, Bue M, Tøttrup M, Friberg LE, Hardlei TF, Søballe K, Gjedsted J. Population Pharmacokinetics of Meropenem in Plasma and Subcutis from Patients on Extracorporeal Membrane Oxygenation Treatment. Antimicrob Agents Chemother. 2018 Apr 26;62(5):e02390-17.
32. Soraluce A, Asín-Prieto E, Rodríguez-Gascón A, Barrasa H, Maynar J, Carcelero E, Soy D, Isla A. Population pharmacokinetics of daptomycin in critically ill patients. Int J Antimicrob Agents. 2018 Aug;52(2):158-165.
33. Kanji S, Roberts JA, Xie J, Alobaid A, Zelenitsky S, Hiremath S, Zhang G, Watpool I, Porteous R, Patel R. Piperacillin Population Pharmacokinetics in Critically Ill Adults During Sustained Low-Efficiency Dialysis. Ann Pharmacother. 2018 Oct;52(10):965-973.
34. Fournier A, Goutelle S, Que YA, Eggimann P, Pantet O, Sadeghipour F, Voirol P, Csajka C. Population Pharmacokinetic Study of Amoxicillin-Treated Burn Patients Hospitalized at a Swiss Tertiary-Care Center. Antimicrob Agents Chemother. 2018 Aug 27;62(9):e00505-18.
35. Tsai D, Stewart PC, Hewagama S, Krishnaswamy S, Wallis SC, Lipman J, Roberts JA. Optimised dosing of vancomycin in critically ill Indigenous Australian patients with severe sepsis. Anaesth Intensive Care. 2018 Jul;46(4):374-380.
36. Turner RB, Kojiro K, Won R, Chang E, Chan D, Elbarbry F. Prospective evaluation of vancomycin pharmacokinetics in a heterogeneous critically ill population. Diagn Microbiol Infect Dis. 2018 Dec;92(4):346-351.
37. Wong G, Briscoe S, McWhinney B, Ally M, Ungerer J, Lipman J, Roberts JA. Therapeutic drug monitoring of β-lactam antibiotics in the critically ill: direct measurement of unbound drug

concentrations to achieve appropriate drug exposures. J Antimicrob Chemother. 2018 Nov 1;73(11):3087-3094.

38. Stein GE, Smith CL, Scharmen A, Kidd JM, Cooper C, Kuti J, Mitra S, Nicolau DP, Havlichek DH. Pharmacokinetic and Pharmacodynamic Analysis of Ceftazidime/Avibactam in Critically Ill Patients. Surg Infect (Larchmt). 2019 Jan;20(1):55-61.
39. Leuppi-Taegtmeyer AB, Decosterd L, Osthoff M, Mueller NJ, Buclin T, Corti N. Multicenter Population Pharmacokinetic Study of Colistimethate Sodium and Colistin Dosed as in Normal Renal Function in Patients on Continuous Renal Replacement Therapy. Antimicrob Agents Chemother. 2019 Jan 29;63(2):e01957-18.
40. Sukarnjanaset W, Jaruratanasirikul S, Wattanavijitkul T. Population pharmacokinetics and pharmacodynamics of piperacillin in critically ill patients during the early phase of sepsis. J Pharmacokinet Pharmacodyn. 2019 Jun;46(3):251-261.
41. Padullés Zamora A, Juvany Roig R, Leiva Badosa E, Sabater Riera J, Pérez Fernández XL, Cárdenas Campos P, Rigo Bonin R, Alía Ramos P, Tubau Quintano F, Sospedra Martinez E, Colom Codina H. Optimized meropenem dosage regimens using a pharmacokinetic/pharmacodynamic population approach in patients undergoing continuous venovenous haemodiafiltration with high-adsorbent membrane. J Antimicrob Chemother. 2019 Oct 1;74(10):2979-2983.
42. Sime FB, Lassig-Smith M, Starr T, Stuart J, Pandey S, Parker SL, Wallis SC, Lipman J, Roberts JA. Population Pharmacokinetics of Unbound Ceftolozane and Tazobactam in Critically Ill Patients without Renal Dysfunction. Antimicrob Agents Chemother. 2019 Sep 23;63(10):e01265-19.
43. Kanji S, Roberts JA, Xie J, Zelenitsky S, Hiremath S, Zhang G, Watpool I, Porteous R, Patel R. Vancomycin Population Pharmacokinetics in Critically Ill Adults During Sustained Low-Efficiency Dialysis. Clin Pharmacokinet. 2020 Mar;59(3):327-334.
44. Sime FB, Lassig-Smith M, Starr T, Stuart J, Pandey S, Parker SL, Wallis SC, Lipman J, Roberts JA. A Population Pharmacokinetic Model-Guided Evaluation of Ceftolozane-Tazobactam Dosing in Critically Ill Patients Undergoing Continuous Venovenous Hemodiafiltration. Antimicrob Agents Chemother. 2019 Dec 20;64(1):e01655-19.
45. Kovacevic T, Miljkovic B, Kovacevic P, Dragic S, Momcicevic D, Avram S, Jovanovic M, Vucicevic K. Population pharmacokinetic model of Vancomycin based on therapeutic drug monitoring data in critically ill septic patients. J Crit Care. 2020 Feb;55:116-121.
46. Kalaria SN, Gopalakrishnan M, Heil EL. A Population Pharmacokinetics and Pharmacodynamic Approach To Optimize Tazobactam Activity in Critically Ill Patients. Antimicrob Agents Chemother. 2020 Feb 21;64(3):e02093-19.
47. Masich AM, Kalaria SN, Gonzales JP, Heil EL, Tata AL, Claeys KC, Patel D, Gopalakrishnan M. Vancomycin Pharmacokinetics in Obese Patients with Sepsis or Septic Shock. Pharmacotherapy. 2020 Mar;40(3):211-220.
48. Bue M, Sou T, Okkels ASL, Hanberg P, Thorsted A, Friberg LE, Andersson TL, Öbrink-Hansen K, Christensen S. Population pharmacokinetics of piperacillin in plasma and subcutaneous tissue in patients on continuous renal replacement therapy. Int J Infect Dis. 2020 Mar;92:133-140.
49. Niibe Y, Suzuki T, Yamazaki S, Suzuki T, Takahashi N, Hattori N, Nakada TA, Oda S, Ishii I. Population Pharmacokinetic Analysis of Meropenem in Critically Ill Patients With Acute Kidney Injury Treated With Continuous Hemodiafiltration. Ther Drug Monit. 2020 Aug;42(4):588-594
50. Zheng J, Sun Z, Sun L, Zhang X, Hou G, Han Q, Li X, Liu G, Gao Y, Ye M, Wang H, Yu K. Pharmacokinetics and Pharmacodynamics of Linezolid in Patients With Sepsis Receiving Continuous Venovenous Hemofiltration and Extended Daily Hemofiltration. J Infect Dis. 2020 Mar 16;221(Suppl 2):S279-S287.
51. Onichimowski D, Będźkowska A, Ziółkowski H, Jaroszewski J, Borys M, Czuczwar M, Wiczling P. Population pharmacokinetics of standard-dose meropenem in critically ill patients on continuous renal replacement therapy: a prospective observational trial. Pharmacol Rep. 2020 Jun;72(3):719-729.
52. Li Z, Bai J, Wen A, Shen S, Duan M, Li X. Pharmacokinetic and Pharmacodynamic Analysis of Critically Ill Patients Undergoing Continuous Renal Replacement Therapy With Imipenem. Clin Ther. 2020 Aug;42(8):1564-1577.e8.

53. Smit C, van Schip AM, van Dongen EPA, Brüggemann RJM, Becker ML, Knibbe CAJ. Dose recommendations for gentamicin in the real-world obese population with varying body weight and renal (dys)function. J Antimicrob Chemother. 2020 Nov 1;75(11):3286-3292.
54. Blackman AL, Jarugula P, Nicolau DP, Chui SH, Joshi M, Heil EL, Gopalakrishnan M. Evaluation of Linezolid Pharmacokinetics in Critically Ill Obese Patients with Severe Skin and Soft Tissue Infections. Antimicrob Agents Chemother. 2021 Jan 20;65(2):e01619-20.
55. Ulldemolins M, Bastida C, Llauradó-Serra M, Csajka C, Rodríguez A, Badia JR, Martín-Loeches I, Soy D. Once-daily 1 g ceftriaxone optimizes exposure in patients with septic shock and hypoalbuminemia receiving continuous veno-venous hemodiafiltration. Eur J Clin Pharmacol. 2021 Aug;77(8):1169-1180.
56. Wang X, Wang Y, Yao F, Chen S, Hou Y, Zheng Z, Luo J, Qiu B, Li Z, Wang Y, Wu Z, Lan J, Chen C. Pharmacokinetics of Linezolid Dose Adjustment for Creatinine Clearance in Critically Ill Patients: A Multicenter, Prospective, Open-Label, Observational Study. Drug Des Devel Ther. 2021 May 19;15:2129-2141.
57. De Winter S, van Hest R, Dreesen E, Annaert P, Wauters J, Meersseman W, Van den Eede N, Desmet S, Verelst S, Vanbrabant P, Peetermans W, Spriet I. Quantification and Explanation of the Variability of First-Dose Amikacin Concentrations in Critically Ill Patients Admitted to the Emergency Department: A Population Pharmacokinetic Analysis. Eur J Drug Metab Pharmacokinet. 2021 Sep;46(5):653-663.
58. Cheng V, Abdul-Aziz MH, Burrows F, Buscher H, Corley A, Diehl A, Jakob SM, Levkovich BJ, Pellegrino V, Que YA, Reynolds C, Rudham S, Wallis SC, Welch SA, Zacharias D, Roberts JA, Shekar K, Fraser JF. Population pharmacokinetics of cefepime in critically ill patients receiving extracorporeal membrane oxygenation (an ASAP ECMO study). Int J Antimicrob Agents. 2021 Dec;58(6):106466.
59. Lan J, Wu Z, Wang X, Wang Y, Yao F, Zhao BX, Wang Y, Chen J, Chen C. Population Pharmacokinetics Analysis and Dosing Simulations Of Meropenem in Critically Ill Patients with Pulmonary Infection. J Pharm Sci. 2022 Jun;111(6):1833-1842.
60. Dreesen E, Gijsen M, Elkayal O, Annaert P, Debaveye Y, Wauters J, Karlsson MO, Spriet I. Ceftriaxone dosing based on the predicted probability of augmented renal clearance in critically ill patients with pneumonia. J Antimicrob Chemother. 2022 Aug 25;77(9):2479-2488.
61. Meenks SD, le Noble JLML, Foudraine NA, de Vries F, Neef K, Janssen PKC. Population pharmacokinetics of unbound ceftriaxone in a critically ill population. Int J Clin Pharmacol Ther. 2022 Sep;60(9):373-383. doi: 10.5414/CP204181. PMID: 35861497.
62. Alshaer MH, Barlow B, Maranchick N, Moser M, Gramss L, Burgmann H, Jalali VA, Wölfl-Duchek M, Jäger W, Poschner S, Plöchl W, Reinprecht A, Rössler K, Gruber A, Zeitlinger M, Peloquin CA, Hosmann A. Meropenem Population Pharmacokinetics and Simulations in Plasma, Cerebrospinal Fluid, and Brain Tissue. Antimicrob Agents Chemother. 2022 Aug 16;66(8):e0043822.
63. Kumta N, Heffernan AJ, Cotta MO, Wallis SC, Livermore A, Starr T, Wong WT, Joynt GM, Lipman J, Roberts JA. Plasma and Cerebrospinal Fluid Population Pharmacokinetics of Meropenem in Neurocritical Care Patients: a Prospective Two-Center Study. Antimicrob Agents Chemother. 2022 Aug 16;66(8):e0014222.
64. Fukumoto S, Ohbayashi M, Okada A, Kohyama N, Tamatsukuri T, Inoue H, Kato A, Kotani T, Sagara H, Dohi K, Kogo M. Population Pharmacokinetic Model and Dosing Simulation of Meropenem Using Measured Creatinine Clearance for Patients with Sepsis. Ther Drug Monit. 2023 Jun 1;45(3):392-399.
65. Meenks SD, Punt N, le Noble JLML, Foudraine NA, Neef K, Janssen PKC. Target attainment and population pharmacokinetics of flucloxacillin in critically ill patients: a multicenter study. Crit Care. 2023 Mar 3;27(1):82.
66. Tang T, Li Y, Xu P, Zhong Y, Yang M, Ma W, Xiang D, Zhang B, Zhou Y. Optimization of polymyxin B regimens for the treatment of carbapenem-resistant organism nosocomial pneumonia: a real-world prospective study. Crit Care. 2023 Apr 28;27(1):164.
67. Wang Y, Yao F, Chen S, Ouyang X, Lan J, Wu Z, Wang Y, Chen J, Wang X, Chen C. Optimal Teicoplanin Dosage Regimens in Critically Ill Patients: Population Pharmacokinetics and Dosing

Simulations Based on Renal Function and Infection Type. Drug Des Devel Ther. 2023 Aug 1;17:2259-2271.
68. Barreto EF, Chang J, Rule AD, Mara KC, Meade LA, Paul J, Jannetto PJ, Athreya AP, Scheetz MH; BLOOM Study Group. Population pharmacokinetic model of cefepime for critically ill adults: a comparative assessment of eGFR equations. Antimicrob Agents Chemother. 2023 Nov 15;67(11):e0081023.
69. Facca B, Frame B, Triesenberg S. Population pharmacokinetics of ceftizoxime administered by continuous infusion in clinically ill adult patients. Antimicrob Agents Chemother. 1998 Jul;42(7):1783-7.
70. Frame BC, Facca BF, Nicolau DP, Triesenberg SN. Population pharmacokinetics of continuous infusion ceftazidime. Clin Pharmacokinet. 1999 Oct;37(4):343-50. doi: 10.2165/00003088-199937040-00005.
71. Dailly E, Arnould JF, Fraissinet F, Naux E, Letard de la Bouralière MA, Bouquié R, Deslandes G, Jolliet P, Le Floch R. Pharmacokinetics of ertapenem in burns patients. Int J Antimicrob Agents. 2013 Jul;42(1):48-52.
72. Beumier M, Roberts JA, Kabtouri H, Hites M, Cotton F, Wolff F, Lipman J, Jacobs F, Vincent JL, Taccone FS. A new regimen for continuous infusion of vancomycin during continuous renal replacement therapy. J Antimicrob Chemother. 2013 Dec;68(12):2859-65.
73. Öbrink-Hansen K, Juul RV, Storgaard M, Thomsen MK, Hardlei TF, Brock B, Kreilgaard M, Gjedsted J. Population pharmacokinetics of piperacillin in the early phase of septic shock: does standard dosing result in therapeutic plasma concentrations? Antimicrob Agents Chemother. 2015 Nov;59(11):7018-26.
74. Lin WW, Wu W, Jiao Z, Lin RF, Jiang CZ, Huang PF, Liu YW, Wang CL. Population pharmacokinetics of vancomycin in adult Chinese patients with post-craniotomy meningitis and its application in individualised dosage regimens. Eur J Clin Pharmacol. 2016 Jan;72(1):29-37.
75. Ide T, Takesue Y, Ikawa K, Morikawa N, Ueda T, Takahashi Y, Nakajima K, Takeda K, Nishi S. Population pharmacokinetics/pharmacodynamics of linezolid in sepsis patients with and without continuous renal replacement therapy. Int J Antimicrob Agents. 2018 May;51(5):745-751.
76. Kang S, Jang JY, Hahn J, Kim D, Lee JY, Min KL, Yang S, Wi J, Chang MJ. Dose Optimization of Cefpirome Based on Population Pharmacokinetics and Target Attainment during Extracorporeal Membrane Oxygenation. Antimicrob Agents Chemother. 2020 Apr 21;64(5):e00249-20.
77. Grensemann J, Busse D, König C, Roedl K, Jäger W, Jarczak D, Iwersen-Bergmann S, Manthey C, Kluge S, Kloft C, Fuhrmann V. Acute-on-chronic liver failure alters meropenem pharmacokinetics in critically ill patients with continuous hemodialysis: an observational study. Ann Intensive Care. 2020 Apr 22;10(1):48.
78. Liu D, Chen W, Wang Q, Li M, Zhang Z, Cui G, Li P, Zhang X, Ma Y, Zhan Q, Wang C. Influence of venovenous extracorporeal membrane oxygenation on pharmacokinetics of vancomycin in lung transplant recipients. J Clin Pharm Ther. 2020 Oct;45(5):1066-1075.
79. Ruiz J, Ramirez P, Villarreal E, Gordon M, Sánchez MÁ, Martín M, Castellanos-Ortega Á. Effect of pharmacokinetic/pharmacodynamic ratio on tigecycline clinical response and toxicity in critically ill patients with multidrug-resistant Gram-negative infections. SAGE Open Med. 2020 Sep 18;8:2050312120958897
80. Lin Z, Chen DY, Zhu YW, Jiang ZL, Cui K, Zhang S, Chen LH. Population pharmacokinetic modeling and clinical application of vancomycin in Chinese patients hospitalized in intensive care units. Sci Rep. 2021 Jan 29;11(1):2670.
81. Šíma M, Michaličková D, Ryšánek P, Cihlářová P, Kuchař M, Lžičařová D, Beroušek J, Hartinger JM, Vymazal T, Slanař O. No Time Dependence of Ciprofloxacin Pharmacokinetics in Critically Ill Adults: Comparison of Individual and Population Analyses. Pharmaceutics. 2021 Jul 27;13(8):1156.
82. Zhao YC, Zou Y, Xiao YW, Wang F, Zhang BK, Xiang DX, Yu F, Luo H, Sandaradura I, Yan M. Does Prolonged Infusion Time Really Improve the Efficacy of Meropenem Therapy? A Prospective Study in Critically Ill Patients. Infect Dis Ther. 2022 Feb;11(1):201-216.

83. Alsultan A, Dasuqi SA, Aljamaan F, Omran RA, Syed SA, AlJaloud T, AlAhmadi A, Alqahtani S, Hamad MA. Pharmacokinetics of meropenem in critically ill patients in Saudi Arabia. Saudi Pharm J. 2021 Nov;29(11):1272-1277.
84. Wang C, Zhang C, Li X, Zhao S, He N, Zhai S, Ge Q. Dose Optimization of Vancomycin for Critically Ill Patients Undergoing CVVH: A Prospective Population PK/PD Analysis. Antibiotics (Basel). 2021 Nov 13;10(11):1392.
85. Busse D, Simon P, Schmitt L, Petroff D, Dorn C, Dietrich A, Zeitlinger M, Huisinga W, Michelet R, Wrigge H, Kloft C. Comparative Plasma and Interstitial Tissue Fluid Pharmacokinetics of Meropenem Demonstrate the Need for Increasing Dose and Infusion Duration in Obese and Non-obese Patients. Clin Pharmacokinet. 2022 May;61(5):655-672.
86. Farkas A, Oikonomou K, Ghanbar M, Villasurda P, Varghese J, Lipman J, Sassine J, Ranganathan D, Roberts JA. Population Pharmacokinetics of Intraperitoneal Gentamicin and the Impact of Varying Dwell Times on Pharmacodynamic Target Attainment in Patients with Acute Peritonitis Undergoing Peritoneal Dialysis. Antimicrob Agents Chemother. 2022 Feb 15;66(2):e0167921.
87. Hahn J, Min KL, Kang S, Yang S, Park MS, Wi J, Chang MJ. Population Pharmacokinetics and Dosing Optimization of Piperacillin-Tazobactam in Critically Ill Patients on Extracorporeal Membrane Oxygenation and the Influence of Concomitant Renal Replacement Therapy. Microbiol Spectr. 2021 Dec 22;9(3):e0063321.
88. Gijsen M, Elkayal O, Annaert P, Van Daele R, Meersseman P, Debaveye Y, Wauters J, Dreesen E, Spriet I. Meropenem Target Attainment and Population Pharmacokinetics in Critically Ill Septic Patients with Preserved or Increased Renal Function. Infect Drug Resist. 2022 Jan 8;15:53-62.
89. Pressiat C, Kudela A, De Roux Q, Khoudour N, Alessandri C, Haouache H, Vodovar D, Woerther PL, Hutin A, Ghaleh B, Hulin A, Mongardon N. Population Pharmacokinetics of Amikacin in Patients on Veno-Arterial Extracorporeal Membrane Oxygenation. Pharmaceutics. 2022 Jan 26;14(2):289.
90. Alihodzic D, Wicha SG, Frey OR, König C, Baehr M, Jarczak D, Kluge S, Langebrake C. Ciprofloxacin in Patients Undergoing Extracorporeal Membrane Oxygenation (ECMO): A Population Pharmacokinetic Study. Pharmaceutics. 2022 Apr 29;14(5):965.
91. Šíma M, Bobek D, Cihlářová P, Ryšánek P, Roušarová J, Beroušek J, Kuchař M, Vymazal T, Slanař O. Factors Affecting the Metabolic Conversion of Ciprofloxacin and Exposure to Its Main Active Metabolites in Critically Ill Patients: Population Pharmacokinetic Analysis of Desethylene Ciprofloxacin. Pharmaceutics. 2022 Aug 4;14(8):1627.
92. Kang S, Yang S, Hahn J, Jang JY, Min KL, Wi J, Chang MJ. Dose Optimization of Meropenem in Patients on Veno-Arterial Extracorporeal Membrane Oxygenation in Critically Ill Cardiac Patients: Pharmacokinetic/Pharmacodynamic Modeling. J Clin Med. 2022 Nov 8;11(22):6621.
93. An G, Creech CB, Wu N, Nation RL, Gu K, Nalbant D, Jimenez-Truque N, Fissell W, Rolsma S, Patel PC, Watanabe A, Fishbane N, Kirkpatrick CMJ, Landersdorfer CB, Winokur P. Evaluation of Empirical Dosing Regimens for Meropenem in Intensive Care Unit Patients Using Population Pharmacokinetic Modeling and Target Attainment Analysis. Antimicrob Agents Chemother. 2023 Jan 24;67(1):e0131222.
94. Bai J, Wen A, Li Z, Li X, Duan M. Population pharmacokinetics and dosing optimisation of imipenem in critically ill patients. Eur J Hosp Pharm. 2023 Mar 22:ejhpharm-2022-003403.
95. Martínez-Casanova J, Esteve-Pitarch E, Colom-Codina H, Gumucio-Sanguino VD, Cobo-Sacristán S, Shaw E, Maisterra-Santos K, Sabater-Riera J, Pérez-Fernandez XL, Rigo-Bonnin R, Tubau-Quintano F, Carratalà J, Padullés-Zamora A. Predictive Factors of Piperacillin Exposure and the Impact on Target Attainment after Continuous Infusion Administration to Critically Ill Patients. Antibiotics (Basel). 2023 Mar 7;12(3):531.
96. An G, Creech CB, Wu N, Nation RL, Gu K, Nalbant D, Jimenez-Truque N, Fissell W, Patel PC, Fishbane N, Watanabe A, Rolsma S, Kirkpatrick CMJ, Landersdorfer CB, Winokur P. Population pharmacokinetics and target attainment analyses to identify a rational empirical dosing strategy for cefepime in critically ill patients. J Antimicrob Chemother. 2023 Jun 1;78(6):1460-1470.
97. Bilal M, Zoller M, Fuhr U, Jaehde U, Ullah S, Liebchen U, Büsker S, Zander J, Babouee Flury B, Taubert M. Cefepime Population Pharmacokinetics, Antibacterial Target Attainment, and

Estimated Probability of Neurotoxicity in Critically Ill Patients. Antimicrob Agents Chemother. 2023 Jul 18;67(7):e0030923.

98. Ehmann L, Zoller M, Minichmayr IK, Scharf C, Huisinga W, Zander J, Kloft C. Development of a dosing algorithm for meropenem in critically ill patients based on a population pharmacokinetic/pharmacodynamic analysis. Int J Antimicrob Agents. 2019 Sep;54(3):309-317.
99. Soraluce A, Barrasa H, Asín-Prieto E, Sánchez-Izquierdo JÁ, Maynar J, Isla A, Rodríguez-Gascón A. Novel Population Pharmacokinetic Model for Linezolid in Critically Ill Patients and Evaluation of the Adequacy of the Current Dosing Recommendation. Pharmaceutics. 2020 Jan 9;12(1):54.
100. Lee JH, Lee DH, Kim JS, Jung WB, Heo W, Kim YK, Kim SH, No TH, Jo KM, Ko J, Lee HY, Jun KR, Choi HS, Jang JH, Jang HJ. Pharmacokinetics and Monte Carlo Simulation of Meropenem in Critically Ill Adult Patients Receiving Extracorporeal Membrane Oxygenation. Front Pharmacol. 2021 Nov 1;12:768912.
101. Dedkaew T., Cressey T.R., Punyawudho B., Lucksiri A. Pharmacokinetics of vancomycin in critically ill patients in Thailand. Int J Pharm Pharm Sci, Vol 7, Issue 9, 232-237
102. Seo H, Kim YK, Park S, Kim HI, Lee DH. Population Pharmacokinetics and Monte Carlo Simulation of Cefepime in Critically Ill Patients with Hospital-Acquired/Ventilator-Associated Pneumonia. Infect Chemother. 2023 Mar;55(1):29-41
103. Mattioli F, Fucile C, Del Bono V, Marini V, Parisini A, Molin A, Zuccoli ML, Milano G, Danesi R, Marchese A, Polillo M, Viscoli C, Pelosi P, Martelli A, Di Paolo A. Population pharmacokinetics and probability of target attainment of meropenem in critically ill patients. Eur J Clin Pharmacol. 2016 Jul;72(7):839-48.
104. Abdulla A, Rogouti O, Hunfeld NGM, Endeman H, Dijkstra A, van Gelder T, Muller AE, de Winter BCM, Koch BCP. Population pharmacokinetics and target attainment of ciprofloxacin in critically ill patients. Eur J Clin Pharmacol. 2020 Jul;76(7):957-967.
105. Cheng V, Abdul-Aziz MH, Burrows F, Buscher H, Corley A, Diehl A, Levkovich BJ, Pellegrino V, Reynolds C, Rudham S, Wallis SC, Welch SA, Roberts JA, Shekar K, Fraser JF; ASAP ECMO Investigators. Population pharmacokinetics of ciprofloxacin in critically ill patients receiving extracorporeal membrane oxygenation (an ASAP ECMO study). Anaesth Crit Care Pain Med. 2022 Jun;41(3):101080.
106. Isla A, Rodríguez-Gascón A, Trocóniz IF, Bueno L, Solinís MA, Maynar J, Sánchez-Izquierdo JA, Pedraz JL. Population pharmacokinetics of meropenem in critically ill patients undergoing continuous renal replacement therapy. Clin Pharmacokinet. 2008;47(3):173-80.
107. Alobaid AS, Wallis SC, Jarrett P, Starr T, Stuart J, Lassig-Smith M, Mejia JL, Roberts MS, Roger C, Udy AA, Lipman J, Roberts JA. Population Pharmacokinetics of Piperacillin in Nonobese, Obese, and Morbidly Obese Critically Ill Patients. Antimicrob Agents Chemother. 2017 Feb 23;61(3):e01276-16.
108. Kim YK, Kim HS, Park S, Kim HI, Lee SH, Lee DH. Population pharmacokinetics of piperacillin/tazobactam in critically ill Korean patients and the effects of extracorporeal membrane oxygenation. J Antimicrob Chemother. 2022 Apr 27;77(5):1353-1364.
109. Ernest D, Cutler DJ. Gentamicin clearance during continuous arteriovenous hemodiafiltration. Crit Care Med. 1992 May;20(5):586-9.
110. Shikuma LR, Ackerman BH, Weaver RH, Solem LD, Strate RG, Cerra FB, Zaske DE. Effects of treatment and the metabolic response to injury on drug clearance: a prospective study with piperacillin. Crit Care Med. 1990 Jan;18(1):37-41.
111. Cornwell EE 3rd, Belzberg H, Berne TV, Gill MA, Theodorou D, Kern JW, Yu W, Asensio J, Demetriades D. Pharmacokinetics of aztreonam in critically ill surgical patients. Am J Health Syst Pharm. 1997 Mar 1;54(5):537-40.
112. Giles LJ, Jennings AC, Thomson AH, Creed G, Beale RJ, McLuckie A. Pharmacokinetics of meropenem in intensive care unit patients receiving continuous veno-venous hemofiltration or hemodiafiltration. Crit Care Med. 2000 Mar;28(3):632-7.
113. Barletta JF, Johnson SB, Nix DE, Nix LC, Erstad BL. Population pharmacokinetics of aminoglycosides in critically ill trauma patients on once-daily regimens. J Trauma. 2000 Nov;49(5):869-72.

114. Malone RS, Fish DN, Abraham E, Teitelbaum I. Pharmacokinetics of cefepime during continuous renal replacement therapy in critically ill patients. Antimicrob Agents Chemother. 2001 Nov;45(11):3148-55.
115. Traunmüller F, Schenk P, Mittermeyer C, Thalhammer-Scherrer R, Ratheiser K, Thalhammer F. Clearance of ceftazidime during continuous venovenous haemofiltration in critically ill patients. J Antimicrob Chemother. 2002 Jan;49(1):129-34.
116. Uchino S, Cole L, Morimatsu H, Goldsmith D, Bellomo R. Clearance of vancomycin during high-volume haemofiltration: impact of pre-dilution. Intensive Care Med. 2002 Nov;28(11):1664-7.
117. Fiaccadori E, Maggiore U, Rotelli C, Giacosa R, Parenti E, Picetti E, Sagripanti S, Manini P, Andreoli R, Cabassi A. Removal of linezolid by conventional intermittent hemodialysis, sustained low-efficiency dialysis, or continuous venovenous hemofiltration in patients with acute renal failure. Crit Care Med. 2004 Dec;32(12):2437-42.
118. Meyer B, Kornek GV, Nikfardjam M, Karth GD, Heinz G, Locker GJ, Jaeger W, Thalhammer F. Multiple-dose pharmacokinetics of linezolid during continuous venovenous haemofiltration. J Antimicrob Chemother. 2005 Jul;56(1):172-9.
119. Fish DN, Teitelbaum I, Abraham E. Pharmacokinetics and pharmacodynamics of imipenem during continuous renal replacement therapy in critically ill patients. Antimicrob Agents Chemother. 2005 Jun;49(6):2421-8.
120. Kielstein JT, Czock D, Schöpke T, Hafer C, Bode-Böger SM, Kuse E, Keller F, Fliser D. Pharmacokinetics and total elimination of meropenem and vancomycin in intensive care unit patients undergoing extended daily dialysis. Crit Care Med. 2006 Jan;34(1):51-6.
121. Klansuwan N, Ratanajamit C, Kasiwong S, Wangsiripaisan A. Clearance of vancomycin during high-efficiency hemodialysis. J Med Assoc Thai. 2006 Jul;89(7):986-91.
122. Bracco D, Landry C, Dubois MJ, Eggimann P. Pharmacokinetic variability of extended interval tobramycin in burn patients. Burns. 2008 Sep;34(6):791-6.
123. Burkhardt O, Hafer C, Langhoff A, Kaever V, Kumar V, Welte T, Haller H, Fliser D, Kielstein JT. Pharmacokinetics of ertapenem in critically ill patients with acute renal failure undergoing extended daily dialysis. Nephrol Dial Transplant. 2009 Jan;24(1):267-71.
124. Golestaneh L, Gofran A, Mokrzycki MH, Chen JL. Removal of vancomycin in sustained low-efficiency dialysis (SLED): a need for better surveillance and dosing. Clin Nephrol. 2009 Oct;72(4):286-91.
125. Deshpande P, Chen J, Gofran A, Murea M, Golestaneh L. Meropenem removal in critically ill patients undergoing sustained low-efficiency dialysis (SLED). Nephrol Dial Transplant. 2010 Aug;25(8):2632-6.
126. Bilgrami I, Roberts JA, Wallis SC, Thomas J, Davis J, Fowler S, Goldrick PB, Lipman J. Meropenem dosing in critically ill patients with sepsis receiving high-volume continuous venovenous hemofiltration. Antimicrob Agents Chemother. 2010 Jul;54(7):2974-8.
127. Taccone FS, Laterre PF, Dugernier T, Spapen H, Delattre I, Wittebole X, De Backer D, Layeux B, Wallemacq P, Vincent JL, Jacobs F. Insufficient β-lactam concentrations in the early phase of severe sepsis and septic shock. Crit Care. 2010;14(4):R126.
128. Seyler L, Cotton F, Taccone FS, De Backer D, Macours P, Vincent JL, Jacobs F. Recommended β-lactam regimens are inadequate in septic patients treated with continuous renal replacement therapy. Crit Care. 2011;15(3):R137.
129. Baptista JP, Sousa E, Martins PJ, Pimentel JM. Augmented renal clearance in septic patients and implications for vancomycin optimisation. Int J Antimicrob Agents. 2012 May;39(5):420-3.
130. Roberts DM, Roberts JA, Roberts MS, Liu X, Nair P, Cole L, Lipman J, Bellomo R; RENAL Replacement Therapy Study Investigators. Variability of antibiotic concentrations in critically ill patients receiving continuous renal replacement therapy: a multicentre pharmacokinetic study. Crit Care Med. 2012 May;40(5):1523-8.
131. Petejova N, Martinek A, Zahalkova J, Duricova J, Brozmannova H, Urbanek K, Grundmann M, Plasek J, Kacirova I. Vancomycin pharmacokinetics during high-volume continuous venovenous hemofiltration in critically ill septic patients. Biomed Pap Med Fac Univ Palacky Olomouc Czech Repub. 2014;158(1):65-72.

132. D'Arcy DM, Casey E, Gowing CM, Donnelly MB, Corrigan OI. An open prospective study of amikacin pharmacokinetics in critically ill patients during treatment with continuous venovenous haemodiafiltration. BMC Pharmacol Toxicol. 2012 Nov 8;13:14.
133. Binder L, Schwörer H, Hoppe S, Streit F, Neumann S, Beckmann A, Wachter R, Oellerich M, Walson PD. Pharmacokinetics of meropenem in critically ill patients with severe infections. Ther Drug Monit. 2013 Feb;35(1):63-70.
134. Adnan S, Li JX, Wallis SC, Rudd M, Jarrett P, Paterson DL, Lipman J, Udy AA, Roberts JA. Pharmacokinetics of meropenem and piperacillin in critically ill patients with indwelling surgical drains. Int J Antimicrob Agents. 2013 Jul;42(1):90-3.
135. Carlier M, Carrette S, Roberts JA, Stove V, Verstraete A, Hoste E, Depuydt P, Decruyenaere J, Lipman J, Wallis SC, De Waele JJ. Meropenem and piperacillin/tazobactam prescribing in critically ill patients: does augmented renal clearance affect pharmacokinetic/pharmacodynamic target attainment when extended infusions are used? Crit Care. 2013 May 3;17(3):R84.
136. Sturm AW, Allen N, Rafferty KD, Fish DN, Toschlog E, Newell M, Waibel B. Pharmacokinetic analysis of piperacillin administered with tazobactam in critically ill, morbidly obese surgical patients. Pharmacotherapy. 2014 Jan;34(1):28-35.
137. Carlier M, Carrette S, Stove V, Verstraete AG, De Waele JJ. Does consistent piperacillin dosing result in consistent therapeutic concentrations in critically ill patients? A longitudinal study over an entire antibiotic course. Int J Antimicrob Agents. 2014 May;43(5):470-3.
138. Huttner A, Von Dach E, Renzoni A, Huttner BD, Affaticati M, Pagani L, Daali Y, Pugin J, Karmime A, Fathi M, Lew D, Harbarth S. Augmented renal clearance, low β-lactam concentrations and clinical outcomes in the critically ill: an observational prospective cohort study. Int J Antimicrob Agents. 2015 Apr;45(4):385-92.
139. Sime FB, Roberts MS, Tiong IS, Gardner JH, Lehman S, Peake SL, Hahn U, Warner MS, Roberts JA. Can therapeutic drug monitoring optimize exposure to piperacillin in febrile neutropenic patients with haematological malignancies? A randomized controlled trial. J Antimicrob Chemother. 2015 Aug;70(8):2369-75.
140. Awissi DK, Beauchamp A, Hébert E, Lavigne V, Munoz DL, Lebrun G, Savoie M, Fagnan M, Amyot J, Tétreault N, Robitaille R, Varin F, Lavallée C, Pichette V, Leblanc M. Pharmacokinetics of an extended 4-hour infusion of piperacillin-tazobactam in critically ill patients undergoing continuous renal replacement therapy. Pharmacotherapy. 2015 Jun;35(6):600-7.
141. Villa G, Cassetta MI, Tofani L, Valente S, Chelazzi C, Falsini S, De Gaudio AR, Novelli A, Ronco C, Adembri C. Linezolid extracorporeal removal during haemodialysis with high cut-off membrane in critically ill patients. Int J Antimicrob Agents. 2015 Oct;46(4):465-8.
142. Wen A, Li Z, Yu J, Li R, Cheng S, Duan M, Bai J. Clinical Validation of Therapeutic Drug Monitoring of Imipenem in Spent Effluent in Critically Ill Patients Receiving Continuous Renal Replacement Therapy: A Pilot Study. PLoS One. 2016 Apr 19;11(4):e0153927.
143. Boucher BA, Hudson JQ, Hill DM, Swanson JM, Wood GC, Laizure SC, Arnold-Ross A, Hu ZY, Hickerson WL. Pharmacokinetics of Imipenem/Cilastatin Burn Intensive Care Unit Patients Undergoing High-Dose Continuous Venovenous Hemofiltration. Pharmacotherapy. 2016 Dec;36(12):1229-1237.
144. Jung B, Mahul M, Breilh D, Legeron R, Signe J, Jean-Pierre H, Uhlemann AC, Molinari N, Jaber S. Repeated Piperacillin-Tazobactam Plasma Concentration Measurements in Severely Obese Versus Nonobese Critically Ill Septic Patients and the Risk of Under- and Overdosing. Crit Care Med. 2017 May;45(5):e470-e478.
145. Roger C, Cotta MO, Muller L, Wallis SC, Lipman J, Lefrant JY, Roberts JA. Impact of renal replacement modalities on the clearance of piperacillin-tazobactam administered via continuous infusion in critically ill patients. Int J Antimicrob Agents. 2017 Aug;50(2):227-231.
146. Carrié C, Petit L, d'Houdain N, Sauvage N, Cottenceau V, Lafitte M, Foumenteze C, Hisz Q, Menu D, Legeron R, Breilh D, Sztark F. Association between augmented renal clearance, antibiotic exposure and clinical outcome in critically ill septic patients receiving high doses of β-lactams administered by continuous infusion: a prospective observational study. Int J Antimicrob Agents. 2018 Mar;51(3):443-449.

147. Ruiz-Ramos J, Villarreal E, Gordon M, Martin-Cerezula M, Broch MJ, Remedios Marqués M, Poveda JL, Castellanos-Ortega Á, Ramírez P. Implication of Haemodiafiltration Flow Rate on Amikacin Pharmacokinetic Parameters in Critically Ill Patients. Blood Purif. 2018;45(1-3):88-94.
148. Fournier A, Eggimann P, Pantet O, Pagani JL, Dupuis-Lozeron E, Pannatier A, Sadeghipour F, Voirol P, Que YA. Impact of Real-Time Therapeutic Drug Monitoring on the Prescription of Antibiotics in Burn Patients Requiring Admission to the Intensive Care Unit. Antimicrob Agents Chemother. 2018 Feb 23;62(3):e01818-17.
149. Sinnollareddy MG, Roberts MS, Lipman J, Peake SL, Roberts JA. Pharmacokinetics of piperacillin in critically ill patients with acute kidney injury receiving sustained low-efficiency diafiltration. J Antimicrob Chemother. 2018 Jun 1;73(6):1647-1650.
150. Wang S, Lin F, Ruan J, Ye H, Wang L. Pharmacokinetics of multiple doses of teicoplanin in Chinese elderly critical patients. Expert Rev Clin Pharmacol. 2018 May;11(5):537-541.
151. Kassel LE, Van Matre ET, Foster CJ, Fish DN, Mueller SW, Sherman DS, Wempe MF, MacLaren R, Neumann RT, Kiser TH. A Randomized Pharmacokinetic and Pharmacodynamic Evaluation of Every 8-Hour and 12-Hour Dosing Strategies of Vancomycin and Cefepime in Neurocritically ill Patients. Pharmacotherapy. 2018 Sep;38(9):921-934.
152. Olbrisch K, Kisch T, Thern J, Kramme E, Rupp J, Graf T, Wicha SG, Mailänder P, Raasch W. After standard dosage of piperacillin plasma concentrations of drug are subtherapeutic in burn patients. Naunyn Schmiedebergs Arch Pharmacol. 2019 Feb;392(2):229-241.
153. Barrasa H, Soraluce A, Isla A, Martín A, Maynar J, Canut A, Sánchez-Izquierdo JA, Rodríguez-Gascón A. Pharmacokinetics of linezolid in critically ill patients on continuous renal replacement therapy: Influence of residual renal function on PK/PD target attainment. J Crit Care. 2019 Apr;50:69-76.
154. Schmidt JJ, Strunk AK, David S, Bode-Böger SM, Martens-Lobenhoffer J, Knitsch W, Scherneck S, Welte T, Kielstein JT. Single- and multiple-dose pharmacokinetics and total removal of colistin in critically ill patients with acute kidney injury undergoing prolonged intermittent renal replacement therapy. J Antimicrob Chemother. 2019 Apr 1;74(4):997-1002.
155. Singhan W, Vadcharavivad S, Areepium N, Wittayalertpanya S, Chaijamorn W, Srisawat N. The effect of direct hemoperfusion with polymyxin B immobilized cartridge on meropenem in critically ill patients requiring renal support. J Crit Care. 2019 Jun;51:71-76.
156. Bouglé A, Dujardin O, Lepère V, Ait Hamou N, Vidal C, Lebreton G, Salem JE, El-Helali N, Petijean G, Amour J. PHARMECMO: Therapeutic drug monitoring and adequacy of current dosing regimens of antibiotics in patients on Extracorporeal Life Support. Anaesth Crit Care Pain Med. 2019 Oct;38(5):493-497.
157. Dhaese SAM, Thooft ADJ, Farkas A, Lipman J, Verstraete AG, Stove V, Roberts JA, De Waele JJ. Early target attainment of continuous infusion piperacillin/tazobactam and meropenem in critically ill patients: A prospective observational study. J Crit Care. 2019 Aug;52:75-79.
158. Leon L, Guerci P, Pape E, Thilly N, Luc A, Germain A, Butin-Druoton AL, Losser MR, Birckener J, Scala-Bertola J, Novy E. Serum and peritoneal exudate concentrations after high doses of β-lactams in critically ill patients with severe intra-abdominal infections: an observational prospective study. J Antimicrob Chemother. 2020 Jan 1;75(1):156-161.
159. Roberts JA, Joynt GM, Lee A, Choi G, Bellomo R, Kanji S, Mudaliar MY, Peake SL, Stephens D, Taccone FS, Ulldemolins M, Valkonen MM, Agbeve J, Baptista JP, Bekos V, Boidin C, Brinkmann A, Buizen L, Castro P, Cole CL, Creteur J, De Waele JJ, Deans R, Eastwood GM, Escobar L, Gomersall C, Gresham R, Jamal JA, Kluge S, König C, Koulouras VP, Lassig-Smith M, Laterre PF, Lei K, Leung P, Lefrant JY, Llauradó-Serra M, Martin-Loeches I, Mat Nor MB, Ostermann M, Parker SL, Rello J, Roberts DM, Roberts MS, Richards B, Rodríguez A, Roehr AC, Roger C, Seoane L, Sinnollareddy M, Sousa E, Soy D, Spring A, Starr T, Thomas J, Turnidge J, Wallis SC, Williams T, Wittebole X, Zikou XT, Paul SK, Lipman J; SMARRT Study Collaborators and the ANZICS Clinical Trials Group. The Effect of Renal Replacement Therapy and Antibiotic Dose on Antibiotic Concentrations in Critically Ill Patients: Data From the Multinational Sampling Antibiotics in Renal Replacement Therapy Study. Clin Infect Dis. 2021 Apr 26;72(8):1369-1378.

160. Gieling EM, Wallenburg E, Frenzel T, de Lange DW, Schouten JA, Ten Oever J, Kolwijck E, Burger DM, Pickkers P, Ter Heine R, Brüggemann RJM. Higher Dosage of Ciprofloxacin Necessary in Critically Ill Patients: A New Dosing Algorithm Based on Renal Function and Pathogen Susceptibility. Clin Pharmacol Ther. 2020 Oct;108(4):770-774.
161. Moni M, Sudhir AS, Dipu TS, Mohamed Z, Prabhu BP, Edathadathil F, Balachandran S, Singh SK, Prasanna P, Menon VP, Patel T, Patel P, Kaye KS, Menon VP. Clinical efficacy and pharmacokinetics of colistimethate sodium and colistin in critically ill patients in an Indian hospital with high endemic rates of multidrug-resistant Gram-negative bacterial infections: A prospective observational study. Int J Infect Dis. 2020 Nov;100:497-506.
162. Lin SY, Shen LJ, Wu VC, Ko WJ, Wu CC, Wu FL. Pharmacokinetics and dosing of vancomycin in patients undergoing sustained low efficiency daily diafiltration (SLEDD-f): A prospective study. J Formos Med Assoc. 2021 Jan;120(1 Pt 3):737-743.
163. Abdulla A, Dijkstra A, Hunfeld NGM, Endeman H, Bahmany S, Ewoldt TMJ, Muller AE, van Gelder T, Gommers D, Koch BCP. Failure of target attainment of beta-lactam antibiotics in critically ill patients and associated risk factors: a two-center prospective study (EXPAT). Crit Care. 2020 Sep 15;24(1):558.
164. Freitas FM, Zamoner W, Reis PFD, Balbi AL, Ponce D. Vancomycin for Dialytic Therapy in Critically Ill Patients: Analysis of Its Reduction and the Factors Associated with Subtherapeutic Concentrations. Int J Environ Res Public Health. 2020 Sep 19;17(18):6861.
165. Kühn D, Metz C, Seiler F, Wehrfritz H, Roth S, Alqudrah M, Becker A, Bracht H, Wagenpfeil S, Hoffmann M, Bals R, Hübner U, Geisel J, Lepper PM, Becker SL. Antibiotic therapeutic drug monitoring in intensive care patients treated with different modalities of extracorporeal membrane oxygenation (ECMO) and renal replacement therapy: a prospective, observational single-center study. Crit Care. 2020 Nov 25;24(1):664.
166. Corcione S, De Nicolò A, Lupia T, Segala FV, Pensa A, Corgiat Loia R, Romeo MR, Di Perri G, Stella M, D'Avolio A, De Rosa FG. Observed concentrations of amikacin and gentamycin in the setting of burn patients with gram-negative bacterial infections: Preliminary data from a prospective study. Therapie. 2021 Sep-Oct;76(5):409-414.
167. Petersson J, Giske CG, Eliasson E. Poor Correlation between Meropenem and Piperacillin Plasma Concentrations and Delivered Dose of Continuous Renal Replacement Therapy. Antimicrob Agents Chemother. 2021 Mar 18;65(4):e02029-20.
168. Nicolau DP, De Waele J, Kuti JL, Caro L, Larson KB, Yu B, Gadzicki E, Zeng Z, Rhee EG, Rizk ML. Pharmacokinetics and Pharmacodynamics of Ceftolozane/Tazobactam in Critically Ill Patients With Augmented Renal Clearance. Int J Antimicrob Agents. 2021 Apr;57(4):106299.
169. Fillâtre P, Lemaitre F, Nesseler N, Schmidt M, Besset S, Launey Y, Maamar A, Daufresne P, Flecher E, Le Tulzo Y, Tadie JM, Tattevin P. Impact of extracorporeal membrane oxygenation (ECMO) support on piperacillin exposure in septic patients: a case-control study. J Antimicrob Chemother. 2021 Apr 13;76(5):1242-1249.
170. Moser S, Rehm S, Guertler N, Hinic V, Dräger S, Bassetti S, Rentsch KM, Sendi P, Osthoff M. Probability of pharmacological target attainment with flucloxacillin in Staphylococcus aureus bloodstream infection: a prospective cohort study of unbound plasma and individual MICs. J Antimicrob Chemother. 2021 Jun 18;76(7):1845-1854.
171. Esteve-Pitarch E, Gumucio-Sanguino VD, Cobo-Sacristán S, Shaw E, Maisterra-Santos K, Sabater-Riera J, Pérez-Fernandez XL, Rigo-Bonnin R, Tubau-Quintano F, Carratalà J, Colom-Codina H, Padullés-Zamora A. Continuous Infusion of Piperacillin/Tazobactam and Meropenem in ICU Patients Without Renal Dysfunction: Are Patients at Risk of Underexposure? Eur J Drug Metab Pharmacokinet. 2021 Jul;46(4):527-538.
172. Pařízková RČ, Martínková J, Havel E, Šafránek P, Kaška M, Astapenko D, Bezouška J, Chládek J, Černý V. Impact of cumulative fluid balance on the pharmacokinetics of extended infusion meropenem in critically ill patients with sepsis. Crit Care. 2021 Jul 17;25(1):251.
173. Liebchen U, Paal M, Bucher V, Vogeser M, Irlbeck M, Schroeder I, Zoller M, Scharf C. Trough concentrations of meropenem and piperacillin during slow extended dialysis in critically ill

patients with intermittent and continuous infusion: A prospective observational study. J Crit Care. 2022 Feb;67:26-32.
174. Messiano CG, Morales Junior R, Pereira GO, Silva Junior EMD, Gomez DS, Santos SRCJ. Therapeutic Target Attainment of 3-Hour Extended Infusion of Meropenem in Patients With Septic Burns. Clin Ther. 2022 Apr;44(4):624-629.
175. Zoller M, Paal M, Greimel A, Kallee S, Vogeser M, Irlbeck M, Schroeder I, Liebchen U, Scharf C. Serum linezolid concentrations are reduced in critically ill patients with pulmonary infections: A prospective observational study. J Crit Care. 2022 Oct;71:154100.
176. Shekar K, Abdul-Aziz MH, Cheng V, Burrows F, Buscher H, Cho YJ, Corley A, Diehl A, Gilder E, Jakob SM, Kim HS, Levkovich BJ, Lim SY, McGuinness S, Parke R, Pellegrino V, Que YA, Reynolds C, Rudham S, Wallis SC, Welch SA, Zacharias D, Fraser JF, Roberts JA. Antimicrobial Exposures in Critically Ill Patients Receiving Extracorporeal Membrane Oxygenation. Am J Respir Crit Care Med. 2023 Mar 15;207(6):704-720.
177. Smeets TJL, de Geus HRH, Rietveld A, Rietdijk WJR, Koch BCP, Endeman H, Hunfeld NGM. Pursuing the Real Vancomycin Clearance during Continuous Renal Replacement Therapy in Intensive Care Unit Patients: Is There Adequate Target Attainment? Blood Purif. 2023;52(7-8):652-659.
178. Correia P, Launay M, Balluet R, Gergele L, Gauthier V, Morel J, Beuret P, Mariat C, Thiery G, Perinel Ragey S. Towards optimization of ceftazidime dosing in obese ICU patients: the end of the 'one-size-fits-all' approach? J Antimicrob Chemother. 2023 Dec 1;78(12):2968-2975.
179. Martin C, Lambert D, Bruguerolle B, Saux P, Freney J, Fleurette J, Meugnier H, Gouin F. Ofloxacin pharmacokinetics in mechanically ventilated patients. Antimicrob Agents Chemother. 1991 Aug;35(8):1582-5.
180. Akers KS, Niece KL, Chung KK, Cannon JW, Cota JM, Murray CK. Modified Augmented Renal Clearance score predicts rapid piperacillin and tazobactam clearance in critically ill surgery and trauma patients. J Trauma Acute Care Surg. 2014 Sep;77(3 Suppl 2):S163-70.
181. Gomez DS, Sanches-Giraud C, Silva CV Jr, Oliveira AM, da Silva JM Jr, Gemperli R, Santos SR. Imipenem in burn patients: pharmacokinetic profile and PK/PD target attainment. J Antibiot (Tokyo). 2015 Mar;68(3):143-7.
182. Ko A, Harada MY, Barmparas G, Jay J, Sun BJ, Chen E, Mehrzadi D, Patel B, Mason R, Ley EJ. Reducing acute kidney injury due to vancomycin in trauma patients. J Trauma Acute Care Surg. 2016 Aug;81(2):352-7.
183. Mokline A, Gharsallah L, Rahmani I, Gaies E, Tabelsi S, Messadi AA. Pharmacokinetics and pharmacodynamics of Linezolid in burn patients. Ann Burns Fire Disasters. 2018 Jun 30;31(2):118-121.
184. Lim SK, Lee SA, Kim CW, Kang E, Choi YH, Park I. High variability of teicoplanin concentration in patients with continuous venovenous hemodiafiltration. Hemodial Int. 2019 Jan;23(1):69-76.
185. Kovacevic T, Miljkovic B, Mikov M, Stojisavljevic Satara S, Dragic S, Momcicevic D, Kovacevic P. The Effect of Hypoalbuminemia on the Therapeutic Concentration and Dosage of Vancomycin in Critically Ill Septic Patients in Low-Resource Countries. Dose Response. 2019 May 20;17(2):1559325819850419.
186. Wu CC, Tai CH, Liao WY, Wang CC, Kuo CH, Lin SW, Ku SC. Augmented renal clearance is associated with inadequate antibiotic pharmacokinetic/pharmacodynamic target in Asian ICU population: a prospective observational study. Infect Drug Resist. 2019 Aug 16;12:2531-2541.
187. Morbitzer KA, Rhoney DH, Dehne KA, Jordan JD. Enhanced renal clearance and impact on vancomycin pharmacokinetic parameters in patients with hemorrhagic stroke. J Intensive Care. 2019 Nov 21;7:51.
188. Breilh D, Honore PM, De Bels D, Roberts JA, Gordien JB, Fleureau C, Dewitte A, Coquin J, Rozé H, Perez P, Attou R, Redant S, Kugener L, Saux MC, Spapen HD, Ouattara A, Joannes-Boyau O; IVOIRE study group. Pharmacokinetics and Pharmacodynamics of Anti-infective Agents during Continuous Veno-venous Hemofiltration in Critically Ill Patients: Lessons Learned from an Ancillary Study of the IVOIRE Trial. J Transl Int Med. 2019 Dec 31;7(4):155-169.

189. Oliveira MS, Machado AS, Mendes ET, Chaves L, Perdigão Neto LV, Vieira da Silva C Jr, Cavani Jorge Santos SR, Sanches C, Macedo E, Levin AS. Pharmacokinetic and Pharmacodynamic Characteristics of Vancomycin and Meropenem in Critically Ill Patients Receiving Sustained Low-efficiency Dialysis. Clin Ther. 2020 Apr;42(4):625-633.
190. Mahmoud AA, Avedissian SN, Al-Qamari A, Bohling T, Pham M, Scheetz MH. Pharmacokinetic Assessment of Pre- and Post-Oxygenator Vancomycin Concentrations in Extracorporeal Membrane Oxygenation: A Prospective Observational Study. Clin Pharmacokinet. 2020 Dec;59(12):1575-1587.
191. Veillette JJ, Winans SA, Maskiewicz VK, Truong J, Jones RN, Forland SC. Pharmacokinetics and Pharmacodynamics of High-Dose Piperacillin-Tazobactam in Obese Patients. Eur J Drug Metab Pharmacokinet. 2021 May;46(3):385-394.
192. Gijsen M, Dreesen E, Van Daele R, Annaert P, Debaveye Y, Wauters J, Spriet I. Pharmacokinetic/Pharmacodynamic Target Attainment Based on Measured versus Predicted Unbound Ceftriaxone Concentrations in Critically Ill Patients with Pneumonia: An Observational Cohort Study. Antibiotics (Basel). 2021 May 11;10(5):557.
193. Shi L, Zhuang Z, Duan L, Zhu C, Xue H, Wang X, Xu X, Yuan Y, Shi L, Li J, Sun J, Liu X, Zhou Q, Lu J, Tang L. Dose Optimization of Teicoplanin for Critically Ill Patients With Renal Dysfunction and Continuous Renal Replacement Therapy: Experience From a Prospective Interventional Study. Front Pharmacol. 2022 Mar 8;13:817401.
194. Zhao J, Fan Y, Yang M, Liang X, Wu J, Chen Y, Guo B, Zhang H, Wang R, Zhang F, Hang J, Li H, Zhang J. Association between Augmented Renal Clearance and Inadequate Vancomycin Pharmacokinetic/Pharmacodynamic Targets in Chinese Adult Patients: A Prospective Observational Study. Antibiotics (Basel). 2022 Jun 22;11(7):837.
195. Calov S, Munzel F, Roehr AC, Frey O, Higuita LMS, Wied P, Rosenberger P, Haeberle HA, Ngamsri KC. Daptomycin Pharmacokinetics in Blood and Wound Fluid in Critical Ill Patients with Left Ventricle Assist Devices. Antibiotics (Basel). 2023 May 13;12(5):904. doi: 10.3390/antibiotics12050904.
196. Tikiso T, Fuhrmann V, König C, Jarczak D, Iwersen-Bergmann S, Kluge S, Wicha SG, Grensemann J. Acute-on-chronic liver failure alters linezolid pharmacokinetics in critically ill patients with continuous hemodialysis: an observational study. Ann Intensive Care. 2023 Sep 12;13(1):83.
197. Roberts DM, Liu X, Roberts JA, Nair P, Cole L, Roberts MS, Lipman J, Bellomo R; RENAL Replacement Therapy Study Investigators. A multicenter study on the effect of continuous hemodiafiltration intensity on antibiotic pharmacokinetics. Crit Care. 2015 Mar 13;19(1):84.
198. Scharf C, Weinelt F, Schroeder I, Paal M, Weigand M, Zoller M, Irlbeck M, Kloft C, Briegel J, Liebchen U. Does the cytokine adsorber CytoSorb® reduce vancomycin exposure in critically ill patients with sepsis or septic shock? a prospective observational study. Ann Intensive Care. 2022 May 23;12(1):44
199. Wulkersdorfer B, Bergmann F, Amann L, Fochtmann-Frana A, Al Jalali V, Kurdina E, Lackner E, Wicha SG, Dorn C, Schäfer B, Ihra G, Rath T, Radtke C, Zeitlinger M. Effect of albumin substitution on pharmacokinetics of piperacillin/tazobactam in patients with severe burn injury admitted to the ICU. J Antimicrob Chemother. 2024 Feb 1;79(2):262-270.
200. Shahrami B, Najmeddin F, Rouini MR, Najafi A, Sadeghi K, Amini S, Khezrnia SS, Sharifnia HR, Mojtahedzadeh M. Evaluation of Amikacin Pharmacokinetics in Critically Ill Patients with Intra-abdominal Sepsis. Adv Pharm Bull. 2020 Jan;10(1):114-118.
201. Simon P, Busse D, Petroff D, Dorn C, Ehmann L, Hochstädt S, Girrbach F, Dietrich A, Zeitlinger M, Kees F, Kloft C, Wrigge H. Linezolid Concentrations in Plasma and Subcutaneous Tissue are Reduced in Obese Patients, Resulting in a Higher Risk of Underdosing in Critically Ill Patients: A Controlled Clinical Pharmacokinetic Study. J Clin Med. 2020 Apr 9;9(4):1067.
202. Del Bono V, Giacobbe DR, Marchese A, Parisini A, Fucile C, Coppo E, Marini V, Arena A, Molin A, Martelli A, Gratarola A, Viscoli C, Pelosi P, Mattioli F. Meropenem for treating KPC-producing Klebsiella pneumoniae bloodstream infections: Should we get to the PK/PD root of the paradox? Virulence. 2017 Jan 2;8(1):66-73.

203. Wu C, Zhang X, Xie J, Li Q, He J, Hu L, Wang H, Liu A, Xu J, Yang C, Yang Y, Qiu H, Huang Y. Pharmacokinetic/Pharmacodynamic Parameters of Linezolid in the Epithelial Lining Fluid of Patients With Sepsis. J Clin Pharmacol. 2022 Jul;62(7):891-897.
204. Corti N, Rudiger A, Chiesa A, Marti I, Jetter A, Rentsch K, Müller D, Béchir M, Maggiorini M. Pharmacokinetics of daily daptomycin in critically ill patients undergoing continuous renal replacement therapy. Chemotherapy. 2013;59(2):143-51.
205. Corcione S, D'Avolio A, Loia RC, Pensa A, Segala FV, De Nicolò A, Fatiguso G, Romeo M, Di Perri G, Stella M, De Rosa FG. Pharmacokinetics of meropenem in burn patients with infections caused by Gram-negative bacteria: Are we getting close to the right treatment? J Glob Antimicrob Resist. 2020 Mar;20:22-27.
206. Lyu Y, Yang Y, Li X, Peng M, He X, Zhang P, Dong S, Wang W, Wang D. Selection of piperacillin/tazobactam infusion mode guided by SOFA score in cancer patients with hospital-acquired pneumonia: a randomized controlled study. Ther Clin Risk Manag. 2017 Dec 21;14:31-37.
207. van der Starre PJ, Kolz M, Lemmens HJ, Faix JD, Mitchell S, Miller C. Vancomycin plasma concentrations in cardiac surgery with the use of profound hypothermic circulatory arrest. Eur J Cardiothorac Surg. 2010 Dec;38(6):741-4.
208. Triginer C, Izquierdo I, Fernández R, Rello J, Torrent J, Benito S, Net A. Gentamicin volume of distribution in critically ill septic patients. Intensive Care Med. 1990;16(5):303-6.
209. Tang GJ, Tang JJ, Lin BS, Kong CW, Lee TY. Factors affecting gentamicin pharmacokinetics in septic patients. Acta Anaesthesiol Scand. 1999 Aug;43(7):726-30.
210. Rebuck JA, Fish DN, Abraham E. Pharmacokinetics of intravenous and oral levofloxacin in critically ill adults in a medical intensive care unit. Pharmacotherapy. 2002 Oct;22(10):1216-25.
211. Belzberg H, Zhu J, Cornwell EE 3rd, Murray JA, Sava J, Salim A, Velmahos GC, Gill MA. Imipenem levels are not predictable in the critically ill patient. J Trauma. 2004 Jan;56(1):111-7.
212. Sánchez Navarro A, Colino Gandarillas CI, Alvarez Lerma F, Menacho YA, Domínguez-Gil A. Pharmacokinetics and pharmacodynamics of levofloxacin in intensive care patients. Clin Pharmacokinet. 2005;44(6):627-35. doi: 10.2165/00003088-200544060-00004. PMID: 15910010.
213. van Zanten AR, Polderman KH, van Geijlswijk IM, van der Meer GY, Schouten MA, Girbes AR. Ciprofloxacin pharmacokinetics in critically ill patients: a prospective cohort study. J Crit Care. 2008 Sep;23(3):422-30.
214. Brink AJ, Richards GA, Schillack V, Kiem S, Schentag J. Pharmacokinetics of once-daily dosing of ertapenem in critically ill patients with severe sepsis. Int J Antimicrob Agents. 2009 May;33(5):432-6.
215. Chapuis TM, Giannoni E, Majcherczyk PA, Chioléro R, Schaller MD, Berger MM, Bolay S, Décosterd LA, Bugnon D, Moreillon P. Prospective monitoring of cefepime in intensive care unit adult patients. Crit Care. 2010;14(2):R51.
216. Aubron C, Corallo CE, Nunn MO, Dooley MJ, Cheng AC. Evaluation of the accuracy of a pharmacokinetic dosing program in predicting serum vancomycin concentrations in critically ill patients. Ann Pharmacother. 2011 Oct;45(10):1193-8.
217. Chung J, Oh JM, Cho EM, Jang HJ, Hong SB, Lim CM, Koh YS. Optimal dose of vancomycin for treating methicillin-resistant Staphylococcus aureus pneumonia in critically ill patients. Anaesth Intensive Care. 2011 Nov;39(6):1030-7.
218. Karnik ND, Sridharan K, Jadhav SP, Kadam PP, Naidu RK, Namjoshi RD, Gupta V, Gore MS, Surase PV, Mehta PR, Gogtay JA, Thatte UM, Gogtay NJ. Pharmacokinetics of colistin in critically ill patients with multidrug-resistant Gram-negative bacilli infection. Eur J Clin Pharmacol. 2013 Jul;69(7):1429-36.
219. Koegelenberg CF, Nortje A, Lalla U, Enslin A, Irusen EM, Rosenkranz B, Seifart HI, Bolliger CT. The pharmacokinetics of enteral antituberculosis drugs in patients requiring intensive care. S Afr Med J. 2013 Apr 5;103(6):394-8.
220. de Montmollin E, Bouadma L, Gault N, Mourvillier B, Mariotte E, Chemam S, Massias L, Papy E, Tubach F, Wolff M, Sonneville R. Predictors of insufficient amikacin peak concentration in critically ill patients receiving a 25 mg/kg total body weight regimen. Intensive Care Med. 2014 Jul;40(7):998-1005.

221. Nakamura A, Takasu O, Sakai Y, Sakamoto T, Yamashita N, Mori S, Morita T, Nabeta M, Hirayu N, Yoshiyama N, Moroki M, Tashiro K, Kannae M. Development of a teicoplanin loading regimen that rapidly achieves target serum concentrations in critically ill patients with severe infections. J Infect Chemother. 2015 Jun;21(6):449-55.
222. Abdul-Aziz MH, Lipman J, Akova M, Bassetti M, De Waele JJ, Dimopoulos G, Dulhunty J, Kaukonen KM, Koulenti D, Martin C, Montravers P, Rello J, Rhodes A, Starr T, Wallis SC, Roberts JA; DALI Study Group. Is prolonged infusion of piperacillin/tazobactam and meropenem in critically ill patients associated with improved pharmacokinetic/pharmacodynamic and patient outcomes? An observation from the Defining Antibiotic Levels in Intensive care unit patients (DALI) cohort. J Antimicrob Chemother. 2016 Jan;71(1):196-207.
223. Zander J, Döbbeler G, Nagel D, Maier B, Scharf C, Huseyn-Zada M, Jung J, Frey L, Vogeser M, Zoller M. Piperacillin concentration in relation to therapeutic range in critically ill patients--a prospective observational study. Crit Care. 2016 Apr 4;20:79.
224. Allou N, Charifou Y, Augustin P, Galas T, Valance D, Corradi L, Martinet O, Vandroux D, Allyn J. A study to evaluate the first dose of gentamicin needed to achieve a peak plasma concentration of 30 mg/l in patients hospitalized for severe sepsis. Eur J Clin Microbiol Infect Dis. 2016 Jul;35(7):1187-93.
225. Obara VY, Zacas CP, Carrilho CM, Delfino VD. Currently used dosage regimens of vancomycin fail to achieve therapeutic levels in approximately 40% of intensive care unit patients. Rev Bras Ter Intensiva. 2016 Oct-Dec;28(4):380-386.
226. Bakke V, Sporsem H, Von der Lippe E, Nordøy I, Lao Y, Nyrerød HC, Sandvik L, Hårvig KR, Bugge JF, Helset E. Vancomycin levels are frequently subtherapeutic in critically ill patients: a prospective observational study. Acta Anaesthesiol Scand. 2017 Jul;61(6):627-635.
227. Ruiz J, Ramirez P, Company MJ, Gordon M, Villarreal E, Concha P, Aroca M, Frasquet J, Remedios-Marqués M, Castellanos-Ortega Á. Impact of amikacin pharmacokinetic/pharmacodynamic index on treatment response in critically ill patients. J Glob Antimicrob Resist. 2018 Mar;12:90-95.
228. Burger R, Guidi M, Calpini V, Lamoth F, Decosterd L, Robatel C, Buclin T, Csajka C, Marchetti O. Effect of renal clearance and continuous renal replacement therapy on appropriateness of recommended meropenem dosing regimens in critically ill patients with susceptible life-threatening infections. J Antimicrob Chemother. 2018 Dec 1;73(12):3413-3422.
229. Huang Y, Yang J, Xie J, Liu L, Liu S, Guo F, Qiu H, Yang Y. Association Between Pathophysiology and Volume of Distribution Among Patients With Sepsis or Septic Shock Treated With Imipenem: A Prospective Cohort Study. J Infect Dis. 2020 Mar 16;221(Suppl 2):S272-S278.
230. Helset E, Nordøy I, Sporsem H, Bakke VD, Bugge JF, Gammelsrud KW, Zucknick M, von der Lippe E. Factors increasing the risk of inappropriate vancomycin therapy in ICU patients: A prospective observational study. Acta Anaesthesiol Scand. 2020 Oct;64(9):1295-1304.
231. Ram K, Sheikh S, Bhati RK, Tripathi CD, Suri JC, Meshram GG. Steady-state pharmacokinetic and pharmacodynamic profiling of colistin in critically ill patients with multi-drug-resistant gram-negative bacterial infections, along with differences in clinical, microbiological and safety outcome. Basic Clin Pharmacol Toxicol. 2021 Jan;128(1):128-140.
232. Ren J, Hou Y, Li J, Gao Y, Li R, Jin X, Zhang J, Wang X, Wang G. An evaluation on the association of vancomycin trough concentration with mortality in critically ill patients: A multicenter retrospective study. Clin Transl Sci. 2021 Sep;14(5):1780-1790.
233. Niibe Y, Suzuki T, Yamazaki S, Uchida M, Suzuki T, Takahashi N, Hattori N, Nakada TA, Ishii I. Identification of factors affecting meropenem pharmacokinetics in critically ill patients: Impact of inflammation on clearance. J Infect Chemother. 2022 Apr;28(4):532-538.
234. Mitton B, Paruk F, Gous A, Chausse J, Milne M, Becker P, Said M. Evaluating the usefulness of the estimated glomerular filtration rate for determination of imipenem dosage in critically ill patients. S Afr Med J. 2022 Aug 30;112(9).
235. Helset E, Cheng V, Sporsem H, Thorstensen C, Nordøy I, Gammelsrud KW, Hanssen G, Ponzi E, Lipman J, von der Lippe E. Meropenem pharmacokinetic/pharmacodynamic target attainment and clinical response in ICU patients: A prospective observational study. Acta Anaesthesiol Scand. 2024 Apr;68(4):502-511.

236. Areskog Lejbman I, Torisson G, Resman F, Sjövall F. Beta-lactam antibiotic concentrations in critically ill patients with standard and adjusted dosages: A prospective observational study. Acta Anaesthesiol Scand. 2024 Apr;68(4):530-537.
237. Beckhouse MJ, Whyte IM, Byth PL, Napier JC, Smith AJ. Altered aminoglycoside pharmacokinetics in the critically ill. Anaesth Intensive Care. 1988 Nov;16(4):418-22.
238. Zeitlinger BS, Zeitlinger M, Leitner I, Müller M, Joukhadar C. Clinical scoring system for the prediction of target site penetration of antimicrobials in patients with sepsis. Clin Pharmacokinet. 2007;46(1):75-83.
239. Cristallini S, Hites M, Kabtouri H, Roberts JA, Beumier M, Cotton F, Lipman J, Jacobs F, Vincent JL, Creteur J, Taccone FS. New Regimen for Continuous Infusion of Vancomycin in Critically Ill Patients. Antimicrob Agents Chemother. 2016 Jul 22;60(8):4750-6.
240. Ehmann L, Zoller M, Minichmayr IK, Scharf C, Maier B, Schmitt MV, Hartung N, Huisinga W, Vogeser M, Frey L, Zander J, Kloft C. Role of renal function in risk assessment of target non-attainment after standard dosing of meropenem in critically ill patients: a prospective observational study. Crit Care. 2017 Oct 21;21(1):263.
241. Coste A, Deslandes G, Jalin L, Corvec S, Caillon J, Boutoille D, Grégoire M, Bretonnière C. PK/PD targets of amikacin and gentamicin in ICU patients. Med Mal Infect. 2020 Nov;50(8):709-714.
242. Logre E, Enser M, Tanaka S, Dubert M, Claudinon A, Grall N, Mentec H, Montravers P, Pajot O. Amikacin pharmacokinetic/pharmacodynamic in intensive care unit: a prospective database. Ann Intensive Care. 2020 Jun 8;10(1):75.
243. De Corte T, Verhaeghe J, Dhaese S, Van Vooren S, Boelens J, G Verstraete A, Stove V, Ongenae F, De Bus L, Depuydt P, Van Hoecke S, J De Waele J. Pathogen-based target attainment of optimized continuous infusion dosing regimens of piperacillin-tazobactam and meropenem in surgical ICU patients: a prospective single center observational study. Ann Intensive Care. 2023 Apr 29;13(1):35.
244. Guilhaumou R, Chevrier C, Setti JL, Jouve E, Marsot A, Julian N, Blin O, Simeone P, Lagier D, Mokart D, Bruder N, Garnier M, Velly L. β-Lactam Pharmacokinetic/Pharmacodynamic Target Attainment in Intensive Care Unit Patients: A Prospective, Observational, Cohort Study. Antibiotics (Basel). 2023 Aug 5;12(8):1289.
245. El-Haffaf I, Marsot A, Hachemi D, Pesout T, Williams V, Smith MA, Albert M, Williamson D. Exposure levels and target attainment of piperacillin/tazobactam in adult patients admitted to the intensive care unit: a prospective observational study. Can J Anaesth. 2024 Apr;71(4):511-522.
246. Campassi ML, Gonzalez MC, Masevicius FD, Vazquez AR, Moseinco M, Navarro NC, Previgliano L, Rubatto NP, Benites MH, Estenssoro E, Dubin A. [Augmented renal clearance in critically ill patients: incidence, associated factors and effects on vancomycin treatment]. Rev Bras Ter Intensiva. 2014 Jan-Mar;26(1):13-20.
247. Zeng J, Leng B, Guan X, Jiang S, Xie M, Zhu W, Tang Y, Zhang L, Sha J, Wang T, Ding M, Guo N, Jiang J. Comparative pharmacokinetics of polymyxin B in critically ill elderly patients with extensively drug-resistant gram-negative bacteria infections. Front Pharmacol. 2024 Feb 1;15:1347130.
248. Galar A, Muñoz P, Valerio M, Cercenado E, García-González X, Burillo A, Sánchez-Somolinos M, Juárez M, Verde E, Bouza E. Current use of daptomycin and systematic therapeutic drug monitoring: Clinical experience in a tertiary care institution. Int J Antimicrob Agents. 2019 Jan;53(1):40-48.
249. Obara VY, Zacas CP, Carrilho CM, Delfino VD. Currently used dosage regimens of vancomycin fail to achieve therapeutic levels in approximately 40% of intensive care unit patients. Rev Bras Ter Intensiva. 2016 Oct-Dec;28(4):380-386.
250. Frazee E, Rule AD, Lieske JC, Kashani KB, Barreto JN, Virk A, Kuper PJ, Dierkhising RA, Leung N. Cystatin C-Guided Vancomycin Dosing in Critically Ill Patients: A Quality Improvement Project. Am J Kidney Dis. 2017 May;69(5):658-666.
251. You X, Dai Q, Hu J, Yu M, Wang X, Weng B, Cheng L, Sun F. Therapeutic drug monitoring of imipenem/cilastatin and meropenem in critically ill adult patients. J Glob Antimicrob Resist. 2024 Mar;36:252-259.

252. Huang F, Cao WX, Yan YY, Mao TT, Wang XW, Huang D, Qiu YS, Lu WJ, Li DJ, Zhuang YG. Influence of continuous renal replacement therapy on the plasma concentration of tigecycline in patients with septic shock: A prospective observational study. Front Pharmacol. 2023 Mar 9;14:1118788. doi: 10.3389/fphar.2023.1118788. PMID: 36969878; PMCID: PMC10034132.
253. Poli E.C., Simoni C., Andr√© P., Buclin T., Longchamp D., Perez M.-H., Ferry T., Schneider A.G. Clindamycin Clearance during Cytosorb Hemoadsorption: A Case Report and Pharmacokinetic Study. Int. J. Artif. Organs. 2019;42:258-262.
254. Dimski T., Brandenburger T., MacKenzie C., Kindgen-Milles D. Elimination of Glycopeptide Antibiotics by Cytokine Hemoadsorption in Patients with Septic Shock: A Study of Three Cases. Int. J. Artif. Organs. 2020;43:753-757.
255. Köhler T, Schwier E, Kirchner C, Winde G, Henzler D, Eickmeyer C. Hemoadsorption with Cytosorb and the Early Course of Linezolid Plasma Concentration during Septic Shock. J. Artif. Organs. 2022. Mar;25(1):86-90
256. Liebchen U., Scharf C., Zoller M., Weinelt F., Kloft C. CytoMero collaboration team No Clinically Relevant Removal of Meropenem by Cytokine Adsorber Cytosorb in Critically Ill Patients with Sepsis or Septic Shock. Intensive Care Med. 2021;47:1332-1333.

File S3

Risk of bias analysis

Figure S1. Review authors' judgements about each risk of bias item for each included randomised controlled trial according to Risk of Bias 2 Assessments.

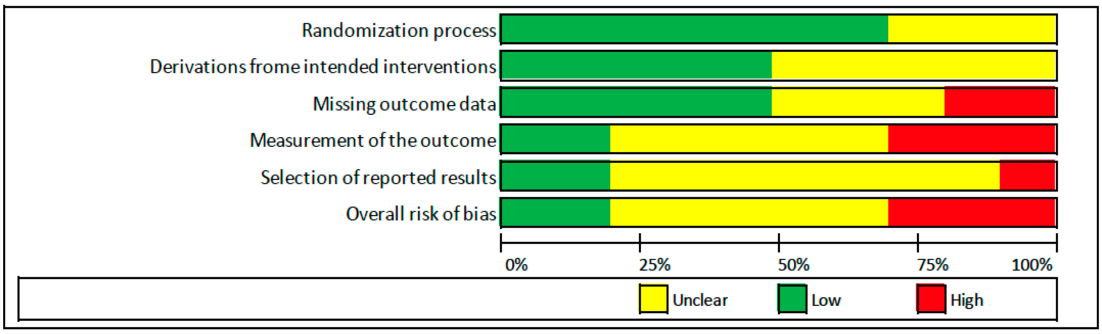

Figure S2. ClinPK reporting quality assessments by study.

	Checklist Item																							
**Author / Year**	**1**	**2**	**3**	**4**	**5**	**6**	**7**	**8**	**9**	**10**	**11**	**12**	**13**	**14**	**15**	**16**	**17**	**18**	**19**	**20**	**21**	**22**	**23**	**24**	**Overall**
Abdul-Aziz 2015	1	1	1	1	1	1		1	1	1	1	1	1	1	1	0		1	1			1	1	1	95,0%
Abdul-Aziz 2016	1	1	1	1	1	1		1	1	1	0	1	1	0	0	0		1	1			1	1		78,9%
Abdulla 2020	1	1	1	1	1	1		1	1	1	1	1	1	0	1	0		1	1	0		1	1	1	85,7%
Abdulla 2020	0	1	1	1	1	1		1	1	1	1	1	1	0	1	0		1	1	0		1	1	1	81,0%
Adnan 2013	1	1	1	1	1	1		1	1	1	1	1	1	1	1	0		1	1			1	1	1	95,0%
Akers 2014	1	1	1	1	1	1		1	1	1	1	1	1	1	1	0		1	1			1	1	1	95,0%
Alihodzic 2022	1	1	1	1	1	1		1	1	1	1	1	1	1	1	0		1	1	1		1	1	1	95,2%
Allou 2016	1	1	1	1	1	1		1	1	1	0	1	1	1	1	0		1	1	0		1	1	1	85,7%
Alobaid 2016	1	1	1	1	1	1		0	1	1	1	1	1	1	1	0		1	1			1	1	1	90,0%
Alobaid 2017	1	1	1	1	1	1		1	1	1	1	1	1	1	1	0		1	1			1	1	1	95,0%
Alshaer 2022	1	1	1	1	1	1		1	1	1	1	1	1	0	1	0		1	1			1	1	1	90,0%
Alsultan 2022	1	1	1	1	1	1		1	1	1	1	1	1	1	1	0		1	1			1	1	1	95,0%
An 2023	1	1	1	1	1	1		0	0	1	1	1	1	1	1	0		1	1	0		1	1	1	81,0%
An 2023	1	1	1	1	1	1		0	1	1	1	1	1	1	0	0		1	1	0		1	1	1	81,0%
Areskog Lejbman 2024	0	1	1	1	1	1		1	1	1	0	1	1	0	1	0		1	1	0		1	1	1	76,2%
Asín-Prieto 2014	1	1	1	1	1	1		1	1	1	1	1	1	1	1	0		1	1	1		1	1	1	95,2%
Aubron 2011	1	1	1	1	1	1		1	1	1	0	1	1	0	1	0		1	1			1	1	1	85,0%
Awissi 2015	1	1	1	1	1	1		1	1	1	1	1	1	1	1	0		1	1	0		1	1	1	90,5%
Bai 2023	1	1	1	1	1	1		1	1	1	1	1	1	1	1	0		1	1			1	1	1	95,0%
Bakke 2017	1	1	1	1	1	1		1	1	1	0	1	1	0	1	0		1	1	0		1	1	1	81,0%
Baptista 2012	1	1	1	1	1	1		1	1	1	1	1	1	1	1	0		1	1			1	1	1	95,0%
Barletta 2000	1	1	1	1	1	1		1	1	1	1	1	1	1	1	0		1	1			1	1	1	95,0%
Barrasa 2019	1	1	1	1	1	1		1	1	1	1	1	1	0	1	0		1	1	1		1	1	1	90,5%
Barreto 2023	1	1	1	1	1	1		1	1	1	1	1	0	0	1	0		1	1			1	1	1	85,0%
Beckhouse 1988	0	1	1	1	1	1		1	1	1	1	1	1	1	1	0		1	1			1	1	1	90,0%
Belzberg 2004	1	1	1	1	1	1		1	1	1	1	1	1	1	1	0		1	1			1	1	1	95,0%
Beumier 2013	1	1	1	1	1	1		1	1	1	1	1	1	1	1	0		1	1	1		1	1	1	95,2%
Bilal 2023	1	1	1	1	1	1		1	1	1	1	1	1	1	1	0		1	1			1	1	1	95,0%
Bilgrami 2010	1	1	1	1	1	1		1	1	1	1	1	1	0	1	0		1	1	1		1	1	1	90,5%
Binder 2013	1	1	1	1	1	1		1	1	1	1	1	1	1	1	0		1	1			1	1	1	95,0%
Blackman 2021	1	1	1	1	1	1		1	1	1	1	1	1	1	1	0		1	1			1	1	1	95,0%
Blassmann 2016	1	1	1	1	1	1		1	1	1	1	1	1	0	1	0		1	1			1	1	1	90,0%
Borsuk-De Moor 2018	1	1	1	1	1	1		1	1	1	1	1	1	0	1	0		1	1	0		1	1	1	85,7%
Bos 2018	1	1	1	1	1	1		1	1	1	1	1	1	1	1	0		1	1			1	1	1	95,0%
Boucher 2016	1	1	1	1	1	1		1	1	1	1	1	1	0	1	0		1	1	1		1	1	1	90,5%
Bougle 2019	1	1	1	1	1	1		1	1	1	1	1	1	1	1	0		1	1			1	1	1	95,0%
Bracco 2008	1	1	1	1	1	1		1	1	1	1	1	1	1	1	0		1	1			1	1	1	95,0%
Braune 2018	1	1	1	1	1	0		1	1	1	1	1	1	0	1	0		1	1	1		1	1	1	85,7%
Breilh 2019	0	0	1	1	1	1		1	1	1	1	1	1	1	1	0	0	1	1	1		1	1	1	81,8%
Brink 2009	1	1	1	1	1	1		1	1	1	1	1	1	1	1	0		1	1			1	1	1	95,0%
Bue 2020	1	1	1	1	1	1		1	1	1	1	1	0	0	1	0		1	1	1		1	1	1	85,7%
Burger 2018	1	1	1	1	1	0		0	1	1	1	1	1	0	1	0		1	1	1		1	1	1	81,0%
Burkhardt 2006	1	1	1	1	1	1		1	1	1	1	1	1	0	0	0		0	1			1	1	1	80,0%
Burkhardt 2009	1	1	1	1	1	1		1	1	1	1	1	1	0	1	0		1	1	1		1	1	1	90,5%

Busse 2022	1	1	1	1	1	1	1	1	1	1	1	1	1	1	0	1	1	1		1	1	1	95,2%
Calov 2023	1	1	1	1	1	1	1	1	1	1	1	1	0	1	0		1	1		1	1	1	90,0%
Campassi 2014	1	1	1	1	1	1	1	1	1	1	1	1	1	1	0		1	1		1	1	1	95,0%
Carlier 2013	1	1	1	1	1	1	1	1	1	1	1	1	0	0	0		1	1		1	1	1	85,0%
Carlier 2014	1	1	1	1	1	1	1	1	1	1	1	1	0	0	0		1	1		1	1	1	85,0%
Carlier 2014	1	1	1	1	1	1	1	1	1	1	1	1	0	0	0		1	1		1	1	1	85,0%
Carrie 2017	0	0	1	1	1	1	1	1	1	0	1	1	1	1	0		1	1	1	1	1	1	81,0%
Černá Pařízková 2021	1	1	1	1	1	1	1	1	1	1	1	1	1	1	0		1	1		1	1	1	95,0%
Chapuis 2010	1	1	1	1	1	1	1	1	1	1	1	1	1	1	0		1	1		1	1	1	95,0%
Cheng 2021	1	1	1	1	1	1	0	1	1	1	1	1	1	1	0		1	1		1	1	1	90,0%
Cheng 2022	1	1	1	1	1	1	0	1	1	1	1	1	0	1	0		1	1	1	1	1	1	85,7%
Chung 2011	1	1	1	1	1	1	0	1	1	1	1	1	1	1	0		1	1	0	1	1	1	85,7%
Corcione 2020	1	1	1	1	1	1	1	1	1	1	1	1	1	1	0		1	1		1	1	1	95,0%
Corcione 2021	1	1	1	1	1	1	1	1	1	0	1	1	0	1	0		1	1		1	1	1	85,0%
Cornwell 1997	1	1	1	1	1	1	1	1	1	1	1	1	1	1	0		1	1		1	1	1	95,0%
Correia 2023	1	1	1	1	1	1	1	1	1	1	1	1	1	1	0		1	1		1	1	1	95,0%
Corti 2013	1	1	1	1	1	1	1	1	1	1	1	1	0	1	0		1	1	1	1	1	1	90,5%
Costea 2020	1	1	1	1	1	1	1	1	1	1	1	1	1	1	0		1	1		1	1	1	95,0%
Couffignal 2014	1	1	1	1	1	1	1	1	0	1	1	1	1	0	1		1	1		0	1	1	85,0%
Cristallini 2016	1	1	1	1	1	1	1	1	1	1	1	1	1	1	0		1	1		1	1	1	95,0%
D'Arcy 2012	1	1	1	1	1	1	1	1	1	1	1	1	0	1	0		1	1	1	1	1	1	90,5%
Dailly 2013	1	1	1	1	1	1	1	1	1	1	1	1	1	1	0		1	1		1	1	1	95,0%
De Corte 2023	1	1	1	1	1	1	1	1	1	0	1	1	0	1	0		1	1	0	1	1	1	81,0%
de Montmollin 2014	1	1	1	1	1	1	1	1	1	0	1	1	1	1	0		1	1	0	1	1	1	85,7%
De Winter 2021	1	1	1	1	1	1	1	1	1	1	1	1	1	1	0		1	1		1	1	1	95,0%
Dedkaew 2015	1	1	1	1	1	1	1	1	1	1	1	1	1	1	0		1	1		1	1	1	95,0%
Del Bono 2017	1	1	1	1	1	1	1	1	1	1	1	1	1	1	0		1	1		1	1	1	95,0%
Deshpande 2010	1	1	1	1	1	1	1	1	1	0	1	1	0	1	0		1	1	1	1	1	1	85,7%
Dhaese 2019	1	1	1	1	1	1	1	1	1	1	1	1	0	1	0		1	1		1	1	1	90,0%
Dimski 2020	1	1	1	1	1	1	1	1	1	1	1	1	1	1	0	1	1	1	0	1	1	1	90,9%
Dreesen 2022	1	1	1	1	1	1	1	1	1	1	1	1	0	1	1		1	1		1	1	1	95,0%
Ehmann 2017	1	1	1	1	1	1	1	1	1	1	1	1	1	1	0		1	1		1	1	1	95,0%
Ehmann 2019	1	1	1	1	1	1	1	1	1	1	1	1	1	1	1	1	1	1		1	1	1	100,0%
El-Haffaf 2024	1	1	1	1	1	1	1	1	1	0	1	1	0	1	0		1	1	0	1	1	1	81,0%
Ernest 1992	1	1	1	1	1	1	1	1	1	1	1	0	1	0	0	1	1	1	0	1	1	1	81,8%
Escobar 2014	1	1	1	1	1	1	1	1	1	1	1	1	0	1	0		1	1	1	1	1	1	90,5%
Esteve-Pitarch 2021	1	1	1	1	1	1	1	1	1	1	1	1	1	1	0		1	1		1	1	1	95,0%
Facca 1998	1	1	1	1	1	1	1	1	1	1	1	1	0	0	0		1	1		1	1	1	85,0%
Farkas 2022	1	1	1	1	1	1	1	1	1	1	1	1	1	1	0		1	1		1	1	1	95,0%
Fiaccadori 2004	1	1	1	1	1	1	1	1	1	1	1	1	0	1	0		1	1	1	1	1	1	90,5%
Fillâtre 2021	1	1	1	1	1	1	1	1	1	1	1	1	0	0	0		1	1	0	1	1	1	81,0%
Fish 2005	1	1	1	1	1	1	1	1	1	1	1	1	0	1	0		1	1	1	1	1	1	90,5%
Fournier 2018	1	1	1	1	1	0	1	1	1	1	0	0	0	0	1	0	1	1	0	1	1	1	68,2%
Fournier 2018	1	1	1	1	1	0	1	1	1	1	1	0	0	0	1		1	1		1	1	1	80,0%
Frame 1999	1	1	1	1	1	1	1	1	1	1	1	1	1	1	0		1	1		1	1	1	95,0%
Frazee 2017	1	1	1	1	1	1	1	1	1	1	1	1	1	1	0		1	1		1	1	1	95,0%
Fukumoto 2023	1	1	1	1	1	1	1	1	1	1	1	1	1	1	0		1	1		1	1	1	95,0%
Galar 2019	1	1	1	1	1	1	1	1	1	0	1	1	0	1	0		1	1	1	1	1	1	85,7%
Gieling 2020	1	1	1	1	1	1	1	1	1	1	1	1	1	1	0		1	1		1	1	1	95,0%
Gijsen 2021	1	1	1	1	1	0	1	1	1	1	1	1	1	1	0		1	1		1	1	1	90,0%

Gijsen 2022	1	1	1	1	1	0	1	1	1	1	1	1	1	1	0	1	1		1	1	1	90,0%
Giles 2000	1	1	1	1	1	1	1	1	1	1	1	1	1	1	0	1	1	1	1	1	1	95,2%
Golestaneh 2009	1	1	1	1	1	1	1	1	1	0	1	1	0	1	0	1	1	1	1	1	1	85,7%
Gomez 2015	1	1	1	1	1	1	1	1	1	1	1	1	1	1	0	1	1		1	1	1	95,0%
Grensemann 2020	1	1	1	1	1	1	1	1	1	1	1	0	0	1	0	0	1	1	1	1	1	81,0%
Guilhaumou 2023	0	1	1	1	1	1	1	1	1	0	1	1	0	0	0	1	1	0	1	1	1	71,4%
Hahn 2021	1	1	1	1	1	1	1	1	1	1	1	1	0	0	0	1	1	1	1	1	1	85,7%
Hanberg 2018	1	1	1	1	1	1	1	1	1	1	1	0	0	1	0	1	1	1	1	1	1	85,7%
Helset 2020	1	1	1	1	1	1	1	1	1	0	1	1	0	1	0	1	1	1	1	1	1	85,7%
Helset 2024	1	1	1	1	1	1	1	1	1	1	1	1	0	1	0	1	1	1	1	1	1	90,5%
Huang 2020	1	1	1	1	1	1	1	1	1	1	1	1	1	1	0	1	1		1	1	1	95,0%
Huang 2023	1	1	1	1	1	1	1	1	1	0	1	1	0	1	0	1	1	1	1	1	1	85,7%
Huttner 2015	0	1	1	1	1	1	1	1	1	1	1	1	1	1	0	1	1		1	1	1	90,0%
Ide 2018	1	1	1	1	1	1	1	1	1	1	1	1	1	1	0	1	1	1	1	1	1	95,2%
Isla 2008	1	1	1	1	1	0	1	1	1	1	1	1	0	0	0	1	1	1	0	1	1	76,2%
Jung 2017	1	1	1	1	1	1	1	1	1	1	1	1	1	1	0	1	1		1	1	1	95,0%
Kalaria 2020	1	1	1	1	1	1	1	1	1	1	1	1	1	1	0	1	1		1	1	1	95,0%
Kang 2020	1	1	1	1	1	1	1	1	1	1	1	1	1	1	0	1	1	1	1	1	1	95,2%
Kang 2022	1	1	1	1	1	1	1	1	1	1	1	1	0	0	0	1	1	1	1	1	1	85,7%
Kanji 2018	1	1	1	1	1	1	1	1	1	1	1	1	0	0	0	1	1	1	1	1	1	85,7%
Kanji 2019	1	1	1	1	1	1	1	1	1	1	1	1	0	1	0	1	1	1	1	1	1	90,5%
Karnik 2013	1	1	1	1	1	1	1	1	1	1	1	1	1	1	0	1	1		1	1	1	95,0%
Kassel 2018	1	1	1	1	1	1	1	1	1	1	1	1	1	1	0	1	1		1	1	1	95,0%
Kees 2015	1	1	1	1	1	1	1	1	1	0	1	1	0	1	0	1	1		1	1	1	85,0%
Kielstein 2006	1	1	1	1	1	1	1	1	1	1	1	1	0	1	0	1	1	1	1	1	1	90,5%
Kim 2022	1	1	1	1	1	1	1	1	1	1	1	1	1	1	0	1	1	0	1	1	1	90,5%
Ko 2016	1	1	1	1	1	1	1	1	1	0	1	1	1	1	0	1	1		1	1	1	90,0%
Koegelenberg 2013	0	1	1	1	1	1	1	1	1	1	1	1	1	1	0	1	1		1	1	1	90,0%
Köhler 2022	1	1	1	1	1	1	1	1	1	1	1	1	0	1	0	1	1	1	1	1	1	90,5%
Kovacevic 2019	1	1	1	1	1	1	1	1	1	1	1	1	1	1	0	1	1		1	1	1	95,0%
Kovacevic 2019	1	1	1	1	1	1	1	1	1	1	1	1	1	1	0	1	1		1	1	1	95,0%
Kühn 2020	0	1	1	1	1	1	1	1	1	0	1	1	0	1	0	1	1	1	1	1	1	81,0%
Kumta 2022	1	1	1	1	1	1	0	0	1	1	1	1	1	1	0	1	1		1	1	0	80,0%
Lan 2022	1	1	1	1	1	1	1	1	1	1	1	1	0	1	0	1	1	0	1	1	1	85,7%
Lee 2021	1	1	1	1	1	0	1	1	1	1	1	1	1	0	0	1	1	1	1	1	1	85,7%
Leon 2020	0	1	1	1	1	1	1	1	1	1	1	1	1	1	0	1	1		1	1	1	90,0%
Leuppi-Taegtmeyer 2019	1	1	1	1	1	1	1	1	1	1	1	1	0	1	0	1	1	1	1	1	1	90,5%
Li 2020	1	1	1	1	1	0	1	1	1	1	1	1	0	1	0	1	1	1	1	1	1	85,7%
Liebchen 2021	1	1	1	1	1	1	0	0	1	0	1	0	1	1	0	1	1		1	1	1	75,0%
Liebchen 2022	1	1	1	1	1	1	1	1	1	1	1	1	0	1	0	1	1	1	1	1	1	90,5%
Lim 2019	1	1	1	1	1	1	1	1	1	1	1	1	0	1	0	1	1	1	1	1	1	90,5%
Lin 2016	1	1	1	1	1	1	1	1	1	1	1	1	1	1	0	1	1		1	1	1	95,0%
Lin 2021	1	1	1	1	1	1	1	1	1	1	1	1	1	1	0	1	1	0	1	1	1	90,5%
Lin 2021	1	1	1	1	1	1	1	1	1	1	1	1	1	1	0	1	1	1	1	1	1	95,2%
Liu 2020	1	1	1	1	1	1	1	1	1	1	1	1	1	1	0	1	1		1	1	1	95,0%
Logre 2020	1	1	1	1	1	1	1	1	1	0	1	1	1	1	0	1	1	0	1	1	1	85,7%
Luque 2014	1	1	1	1	1	1	1	1	1	1	1	1	1	1	0	1	1		1	1	1	95,0%
Lyu 2018	1	1	1	1	1	1	1	1	1	1	1	1	1	1	0	1	1		1	1	1	95,0%
Mahmoud 2020	1	1	1	1	1	1	1	1	1	1	1	1	1	1	0	1	1		1	1	1	95,0%

Malone 2001	1	1	1	1	1	1	1	1	1	1	1	1	0	1	0		1	1	1	1	1	1	90,5%
Martin 1991	1	1	1	1	1	1	1	1	1	1	1	1	1	1	0		1	1		1	1	1	95,0%
Martínez-Casanova 2023	1	1	1	1	1	1	1	1	1	1	1	1	1	1	0		1	1		1	1	1	95,0%
Masich 2020	1	1	1	1	1	1	1	1	1	1	1	1	1	1	0		1	1		1	1	1	95,0%
Mattioli 2016	1	1	1	1	1	1	1	1	1	1	1	1	1	1	0		1	1		1	1	1	95,0%
Meenks 2022	1	1	1	1	1	1	1	1	1	1	1	1	1	1	0		1	1	0	1	1	1	90,5%
Meenks 2023	1	1	1	1	1	1	1	1	1	1	1	1	1	1	0		1	1		1	1	1	95,0%
Messiano 2022	1	1	1	1	1	1	1	1	1	1	1	1	1	1	0		1	1		1	1	1	95,0%
Meyer 2005	1	1	1	1	1	1	1	1	1	1	1	1	1	1	0		1	1	1	1	1	1	95,2%
Mimoz 2006	1	1	1	1	1	1	1	1	1	1	1	1	1	1	0		1	1	1	1	1	1	95,2%
Mitton 2022	1	1	1	1	1	1	1	1	1	0	1	1	1	1	0		1	1		1	1	1	90,0%
Mokline 2018	1	1	1	1	1	1	1	1	1	1	1	1	1	1	0		1	1		1	1	1	95,0%
Moni 2020	1	1	1	1	1	1	1	1	1	1	1	1	1	1	0		1	1		1	1	1	95,0%
Morbitzer 2019	1	1	1	1	1	1	1	1	1	1	1	1	1	1	0		1	1		1	1	1	95,0%
Moreira de Freitas 2020	1	1	1	1	1	1	1	1	1	1	1	1	0	1	0		1	1	1	1	1	1	90,5%
Moser 2021	1	1	1	1	1	1	1	1	1	1	1	1	1	1	0		1	1		1	1	1	95,0%
Naik 2017	1	1	1	1	1	1	1	1	1	1	1	1	1	1	0		1	1		1	1	1	95,0%
Nakamura 2015	1	1	1	1	1	1	1	1	1	0	1	1	1	1	0		1	1	0	1	1	1	85,7%
Nicolau 2021	1	1	1	1	1	1	1	1	1	1	1	1	1	1	0		1	1		1	1	1	95,0%
Niibe 2020	1	1	1	1	1	1	1	1	1	1	1	1	0	0	0		1	1	1	1	1	0	81,0%
Niibe 2022	1	1	1	1	1	1	1	1	1	1	1	1	0	1	0		1	1	1	1	1	1	90,5%
O¨brink-Hansen 2015	1	1	1	1	1	1	1	1	1	1	1	1	0	1	0		1	1		1	1	1	90,0%
Obara 2016	1	1	1	1	1	1	1	1	1	1	1	1	1	1	0		1	1		1	1	1	95,0%
Obara 2016	1	1	1	1	1	1	1	1	1	1	1	1	1	1	0		1	1		1	1	1	95,0%
Olbrisch 2019	1	1	1	1	1	1	1	1	1	1	1	1	1	1	0		1	1		1	1	1	95,0%
Oliveira 2020	1	1	1	1	1	1	1	1	1	1	1	1	1	1	0	1	1	1	1	1	1	1	95,5%
Onichimowski 2020	1	1	1	1	1	1	1	1	1	1	1	1	0	1	0		1	1	1	1	1	1	90,5%
Padulles Zamora 2019	1	1	0	1	1	1	1	1	1	1	1	1	0	1	0		1	1	1	1	1	1	85,7%
Petejova 2012	1	1	1	1	1	1	1	1	1	1	1	1	0	1	0		1	1	1	1	1	1	90,5%
Petersson 2021	1	1	1	1	1	1	1	1	1	1	1	1	1	1	0		1	1	1	1	1	1	95,2%
Poli 2019	1	1	1	1	1	1	1	1	1	1	1	1	1	1	0		1	1	1	1	1	1	95,2%
Pressiat 2022	1	1	1	1	1	1	1	1	1	1	1	1	1	1	0		1	1		1	1	1	95,0%
Rahbar 2016	1	1	1	1	1	1	1	1	0	0	0	1	0	1	1		1	1		1	1	1	80,0%
Ram 2021	1	1	1	1	1	1	1	1	1	1	1	1	1	1	0		1	1		1	1	1	95,0%
Rebuck 2002	1	1	1	1	1	1	1	1	1	1	1	1	1	1	0		1	1		1	1	1	95,0%
Ren 2021	1	1	1	1	1	1	1	1	1	0	1	1	0	1	0		1	1	0	1	1	1	81,0%
Roberts 2010	1	1	1	1	1	0	1	1	1	1	1	1	1	1	0		1	1	0	1	1	1	85,7%
Roberts 2012	0	1	1	1	1	1	1	1	1	0	1	1	0	1	0		1	1	1	1	1	1	81,0%
Roberts 2015	0	1	1	1	1	1	1	1	1	1	1	1	1	1	0	1	1	1	1	1	1	1	90,9%
Roberts 2015	1	1	1	1	1	1	1	1	1	1	1	1	1	1	0		1	1		1	1	1	95,0%
Roberts 2021	0	1	1	1	1	1	1	1	1	0	1	1	0	1	0		1	1	0	1	1	1	76,2%
Roger 2016	1	1	1	1	1	1	1	1	1	1	1	1	1	1	0		1	1	1	1	1	1	95,2%
Roger 2016	1	1	1	1	1	1	1	1	1	1	1	1	0	1	0	0	1	1	1	1	1	1	86,4%
Roger 2017	1	1	1	1	1	1	1	1	1	1	1	1	1	1	0		1	1	1	1	1	1	95,2%
Roos 2007	1	1	1	1	1	0	1	1	1	1	1	1	0	0	0		1	1		1	1	0	75,0%
Ruiz-Ramos 2018	1	1	1	1	1	1	1	1	1	1	1	1	1	1	0		1	1	1	1	1	1	95,2%
Ruiz-ramos 2018	1	1	1	1	1	1	1	1	1	1	1	1	1	1	0		1	1		1	1	1	95,0%
Ruiz-Ramos 2020	1	1	1	1	1	1	1	1	1	1	1	1	0	1	0		1	1	0	1	1	1	85,7%
Sanchez-Navarro 2005	1	1	1	1	1	1	1	1	1	1	1	1	1	1	0		1	1		1	1	1	95,0%

Scharf 2022	1	1	1	1	1	1	1	1	1	1	1	1	0	1	0		1	1	1	1	1	1	90,5%
Schmidt 2019	1	1	1	1	1	1	1	1	1	1	1	1	1	1	0		1	1	1	1	1	1	95,2%
Seo 2023	1	1	1	1	1	1	1	1	1	1	1	1	1	1	0		1	1		1	1	1	95,0%
Seyler 2011	0	1	1	1	1	1	1	1	1	1	1	1	0	1	0		1	1	1	1	1	1	85,7%
Shahrami 2020	1	1	1	1	1	1	1	1	1	1	1	1	1	1	0		1	1		1	1	1	95,0%
Shekar 2023	1	1	1	1	0	1	0	1	1	1	1	0	1	1	0		1	1	0	1	1	1	76,2%
Shi 2022	1	1	1	1	1	1	1	1	1	1	1	1	1	1	0		1	1	1	1	1	1	95,2%
Shikuma 1990	1	1	1	1	1	1	1	1	1	1	1	1	1	1	0		1	1		1	1	1	95,0%
Sima 2021	1	1	1	1	1	1	1	1	1	1	1	1	1	1	0		1	1	0	1	1	1	90,5%
Šíma 2022	1	1	1	1	1	1	1	1	1	0	1	1	0	1	0		1	1		1	1	1	85,0%
Sime 2015	1	1	1	1	1	1	1	1	1	0	1	1	0	1	0		1	1	1	1	1	1	85,7%
Sime 2017	1	1	1	1	1	1	1	1	1	1	1	1	1	1	0		1	1		1	1	1	95,0%
Sime 2019	1	1	1	1	1	1	1	1	1	1	1	1	0	1	0		1	1	1	1	1	1	90,5%
Sime 2019	1	1	1	1	1	1	1	1	1	1	1	1	1	1	0		1	1		1	1	1	95,0%
Simon 2021	1	1	1	1	1	1	1	1	1	1	1	1	1	1	0		1	1	0	1	1	1	90,5%
Singhan 2019	1	1	1	1	1	1	1	1	1	1	1	1	1	1	0		1	1	1	1	1	1	95,2%
Sinnollareddy 2018	1	1	1	1	1	1	1	1	1	1	1	1	1	1	0		1	1	1	1	1	1	95,2%
Sjovall 2018	1	1	1	1	1	1	1	1	1	1	1	1	1	1	0		1	1		1	1	1	95,0%
Smeets 2023	1	1	1	1	1	1	1	1	1	1	1	1	1	1	0		1	1	1	1	1	1	95,2%
Smit 2020	1	1	1	1	1	1	1	1	1	1	1	1	1	1	0		1	1		1	1	1	95,0%
Soraluce 2018	1	1	1	1	1	1	1	1	1	1	1	1	1	1	0		1	1	0	1	1	1	90,5%
Soraluce 2020	1	1	1	1	1	1	1	1	1	1	1	1	1	1	0		1	1	0	1	1	1	90,5%
Stain 2019	1	1	1	1	1	1	1	1	1	1	1	0	1	1	0		1	1		0	1	1	85,0%
Sturm 2013	1	1	1	1	1	1	1	1	1	0	0	0	1	0	0		1	1		1	1	1	75,0%
Sukarnjanaset 2019	1	1	1	1	1	1	1	1	1	1	1	1	1	0	0		1	1		1	1	1	90,0%
Taccone 2010	0	1	1	1	1	1	1	1	1	1	1	1	1	1	0		1	1		1	1	1	90,0%
Tang 1999	1	1	1	1	1	1	1	1	1	1	1	1	1	1	0		1	1		1	1	1	95,0%
Tang 2023	1	1	1	1	1	1	1	1	1	1	1	1	1	1	0		1	1		1	1	1	95,0%
Taubert 2016	1	1	1	1	1	1	1	1	1	1	1	1	1	1	0		1	1	0	1	1	1	90,5%
Tikiso 2023	1	1	1	1	1	1	1	1	1	1	1	1	1	1	0		1	1		1	1	1	95,0%
Traunmüller 2002	1	1	1	1	1	1	1	1	1	1	1	1	1	1	0		1	1	1	1	1	1	95,2%
Triginer 1990	1	1	1	1	1	1	1	1	1	1	1	1	1	1	0		1	1		1	1	1	95,0%
Tsai 2016	1	1	1	1	1	1	1	1	1	1	1	1	1	1	0		1	1		1	1	1	95,0%
Tsai 2018	0	1	1	1	1	1	1	1	1	1	1	1	1	1	0		1	1		1	1	1	90,0%
Turner 2018	1	1	1	1	1	1	1	1	1	1	1	1	1	1	0		1	1		1	1	1	95,0%
Uchino 2002	1	1	1	1	1	1	1	1	1	1	1	1	1	1	0		1	1	1	1	1	1	95,2%
Ulldemolins 2015	1	1	1	1	1	1	1	1	1	1	1	1	1	1	0		1	1	1	1	1	1	95,2%
Ulldemolins 2016	1	1	1	1	1	1	1	1	1	1	1	1	1	1	0		1	1	1	1	1	1	95,2%
Ulldemolins 2021	1	1	1	1	1	1	1	1	1	1	1	1	1	1	0		1	1	1	1	1	1	95,2%
Van der Starre 2010	1	1	1	1	1	1	1	1	1	0	1	1	1	1	0		1	1		1	1	1	90,0%
Van Zanten 2008	1	1	1	1	1	1	1	1	1	1	1	1	1	1	0		1	1		1	1	1	95,0%
Veillette 2021	1	1	1	1	1	1	1	1	1	1	1	1	1	1	0		1	1		1	1	1	95,0%
Villa 2015	1	1	1	1	1	1	1	1	1	1	1	1	0	1	0		1	1	1	1	1	1	90,5%
Wang 2018	1	1	1	1	1	1	1	1	1	1	1	1	1	1	0		1	1		1	1	1	95,0%
Wang 2021	1	1	1	1	1	1	1	1	1	1	1	1	0	1	0		1	1	0	1	1	1	85,7%
Wang 2021	1	1	1	1	1	1	1	1	1	1	1	1	1	1	0		1	1	1	1	1	1	95,2%
Wang 2023	1	1	1	1	1	1	1	1	1	1	1	1	0	1	0		1	1	0	1	1	1	85,7%
Wen 2016	1	1	1	1	1	1	1	1	1	1	1	1	0	1	0		1	1	1	1	1	1	90,5%
Whitehouse 2005	1	1	1	1	1	1	1	1	1	1	1	1	1	1	0	0	1	1	0	1	1	1	86,4%
Wicha 2017	1	1	1	1	1	1	1	1	1	1	1	1	0	1	0		1	1	0	1	1	1	85,7%

Wong 2018	1	1	1	1	1	1	1	1	1	0	1	1	0	1	0	1	1		1	1	1	85,0%
Wu 2019	0	1	1	1	1	1	1	1	1	1	1	1	1	1	0	1	1		1	1	1	90,0%
Wu 2022	1	1	1	1	1	1	1	1	1	1	1	1	1	1	0	1	1		1	1	1	95,0%
Wulkersdorfer 2024	1	1	1	1	1	1	1	1	1	1	1	1	1	1	0	1	1		1	1	1	95,0%
Xie 2017	1	1	1	1	1	1	1	1	1	1	1	1	1	1	0	1	1		1	1	1	95,0%
You 2024	1	1	1	1	1	1	1	1	1	0	1	1	0	1	0	1	1	0	1	1	1	81,0%
Zander 2016	1	1	1	1	1	1	1	1	1	1	1	1	0	1	0	1	1	0	1	1	1	85,7%
Zeitlinger 2007	0	1	1	1	1	1	1	1	1	1	1	1	0	1	0	1	1		1	1	1	85,0%
Zeng 2024	1	1	1	1	1	1	1	1	1	1	1	1	1	1	0	1	1		1	1	1	95,0%
Zhao 2022	1	1	1	1	1	1	1	1	1	1	1	1	1	1	0	1	1		1	1	1	95,0%
Zhao 2022	1	1	1	1	1	1	1	1	1	1	1	1	0	1	0	1	1		1	1	1	90,0%
Zheng 2020	1	1	1	1	1	1	1	1	1	1	1	1	0	1	0	1	1	1	1	1	1	90,5%
Zoller 2014	1	1	1	1	1	1	1	1	1	1	1	1	0	1	0	1	1	0	1	1	1	85,7%
Zoller 2022	1	1	1	1	1	1	1	1	1	0	1	1	1	1	0	1	1		1	1	1	90,0%

File S4

Risk factors that were studied without finding any impact were (indicating the number of studies whenever more than one:

Platelets (8), ethnicity (7), red blood cell count (6), prothrombin time (4), heart rate (4), respiratory rate (2), interleukin-6 (3), cerebrospinal fluid (CSF) drainage (3), Protein in CSF (2), Interleukin-6 in CSF (2), Cells in CSF (2), lactate, pyruvate, glycerol or glutamate in CSF, other drainage, Injury Severity Score, PEEP, PAFI, ASA classification, allogeneic blood products, carbamide, Glucose, Creatine phosphokinase (CPK), potassium, vancomycin manufacturer, uric acid, anesthesia duration, intra-anesthetic or post-anesthetic peritoneal membrane transporter status, cholinesterase, factor V, CD64, Pitt bacteremia score, post-surgical drainage, hypothermia, emergent surgery, Glasgow Coma Scale, SaO2, Simplified Septic Shock Score (SSS), Complete SSS, SIRS, ARCTIC score, ICDSC and drug characteristics like protein plasmatic union percentage.

File S5

Main characteristics of studies included:

Author and year	*Study type*	*Drugs*	*Nº Centers*	*Country*	*Nº Patients*	*Primary endpoint*
Whitehouse 2005	RCT	Linezolid, Teicoplanin	Unicentric	UK	54	PK/PKPOP
Burkhardt 2006	Prospective observational	Ertapenem	Unicentric	Germany	17	PK/PKPOP
Roos 2007	Prospective observational	Cefpirome	Unicentric	Australia	12	PK/PKPOP
Roberts 2010	Prospective observational	Genatmicin	Multicentric	Australia	14	PK/PKPOP
Asín-Prieto 2014	Prospective observational	PTZ	Unicentric	Spain	16	PK/PKPOP

Escobar 2014	Prospective observational	Vancomycin	Unicentric	Chile	9	PK/PKPOP
Couffignal 2014	Prospective observational	Imipenem	Multicentric	France	63	PK/PKPOP
Carlier 2014	Prospective observational	Cefuroxima	Unicentric	Belgium	160	PK/PKPOP
Zoller 2014	Prospective observational	Linezolid	Unicentric	Germany	30	Exposure
Luque 2014	Prospective observational	PTZ	Unicentric	Spain	11	PK/PKPOP
Ulldemolins 2015	Prospective observational	Meropenem	Multicentric	Spain	30	Exposure
Kees 2015	Prospective observational	Meropenem	Unicentric	Germany	32	PK/PKPOP
Abdul-Aziz 2015	Prospective observational	Doripenem	Unicentric	Malaysia	12	PK/PKPOP
Roger 2016	Prospective observational	Linezolid	Unicentric	France	13	PK/PKPOP
Roberts 2015	Prospective observational	Linezolid	Unicentric	Australia	35	PK/PKPOP
Ulldemolins 2016	Prospective observational	Piperacillin	Multicentric	Spain	19	PK/PKPOP
Roger 2016	RCT	Ciprofloxacin	Unicentric	France	11	PK/PKPOP
Alobaid 2016	Prospective observational	Meropenem	Unicentric	Australia	19	PK/PKPOP
Taubert 2016	Prospective observational	Linezolid	Unicentric	Germany	52	PK/PKPOP
Tsai 2016	Prospective observational	Meropenem	Unicentric	Australia	11	PK/PKPOP
Blassmann 2016	Prospective observational	Meropenem	Unicentric	Germany	21	PK/PKPOP
Rahbar 2016	Prospective observational	Doripenem	Unicentric	USA	30	PK/PKPOP
Naik 2017	RCT	Cefazolina	Unicentric	USA	20	PK/PKPOP
Xie 2017	Prospective observational	Tigecycline	Unicentric	China	10	PK/PKPOP
Wicha 2017	Prospective observational	Linezolid	Unicentric	Germany	28	PK/PKPOP
Sime 2017	Prospective observational	PTZ	Unicentric	Australia	37	PK/PKPOP
Sjovall 2018	Prospective observational	Meropenem	Unicentric	Denmark	51	PK/PKPOP
Borsuk-De Moor 2018	Prospective observational	Tigecycline	Multicentric	Poland	37	PK/PKPOP
Braune 2018	Prospective observational	Meropenem	Unicentric	Germany	19	PK/PKPOP
Bos 2018	Prospective observational	Ceftriaxone, cefazolin, Meropenem, ampicillin, benzylpenicillin, flucloxacillin and piperacillin	Unicentric	Mozambiqu e	88	PK/PKPOP
Hanberg 2018	Prospective observational	Meropenem	Unicentric	Denmark	10	PK/PKPOP
Soraluce 2018	Prospective observational	Daptomycin	Multicentric	Spain	16	PK/PKPOP
Kanji 2018	Prospective observational	PTZ	Unicentric	Canada	34	PK/PKPOP
Fournier 2018	Prospective observational	Amoxicillin	Unicentric	Switzerland	21	PK/PKPOP
Tsai 2018	Prospective observational	Vancomycin	Unicentric	Australia	15	PK/PKPOP
Turner 2018	Prospective observational	Vancomycin	Unicentric	USA	19	PK/PKPOP
Wong 2018	Prospective observational	Ceftriaxone, cefazolin, Meropenem, ampicillin, benzylpenicillin, flucloxacillin and piperacillin	Unicentric	Australia	330	Exposure

Stain 2019	Prospective observational	Ceftazidime/avibactam	Unicentric	USA	10	PK/PKPOP
Leuppi-Taegtmeyer 2019	Prospective observational	Colistin	Multicentric	Switzerland	10	PK/PKPOP
Sukarnjanaset 2019	Prospective observational	Piperacillin	Unicentric	Thailand	48	PK/PKPOP
Padulles Zamora 2019	Prospective observational	Meropenem	Unicentric	Spain	12	PK/PKPOP
Sime 2019	Prospective observational	Ceftolozane/tazobactam	Unicentric	Australia	12	PK/PKPOP
Kanji 2019	Prospective observational	Vancomycin	Unicentric	Canada	31	PK/PKPOP
Sime 2019	Prospective observational	Ceftolozane/tazobactam	Unicentric	Australia	6	PK/PKPOP
Kovacevic 2019	Prospective observational	Vancomycin	Unicentric	Bosnia and Herzegovina	73	PK/PKPOP
Kalaria 2020	Prospective observational	Tazobactam	Unicentric	USA	18	PK/PKPOP
Masich 2020	Prospective observational	Vancomycin	Unicentric	USA	16	PK/PKPOP
Bue 2020	Prospective observational	Piperacillin	Unicentric	Denmark	10	PK/PKPOP
Niibe 2020	Prospective observational	Meropenem	Unicentric	Japan	21	PK/PKPOP
Zheng 2020	Prospective observational	Linezolid	Unicentric	China	20	PK/PKPOP
Onichimowski 2020	Prospective observational	Meropenem	Unicentric	Poland	19	PK/PKPOP
Li 2020	Prospective observational	Imipenem	Unicentric	China	30	PK/PKPOP
Smit 2020	Prospective observational	Genatmicin	Multicentric	Dutch	542	PK/PKPOP
Blackman 2021	Prospective observational	Linezolid	Unicentric	USA	11	PK/PKPOP
Ulldemolins 2021	Prospective observational	Ceftriaxone	Multicentric	Spain	8	PK/PKPOP
Wang 2021	Prospective observational	Linezolid	Multicentric	China	117	PK/PKPOP
De Winter 2021	Prospective observational	Amikacin	Unicentric	Belgium	97	PK/PKPOP
Cheng 2021	Prospective observational	Cefepime	Multicentric	New Zealand	6	PK/PKPOP
Lan 2022	Prospective observational	Meropenem	Unicentric	China	48	PK/PKPOP
Dreesen 2022	Prospective observational	Ceftriaxone	Unicentric	Belgium	33	PK/PKPOP
Meenks 2022	Prospective observational	Ceftriaxone	Unicentric	Netherlands	91	PK/PKPOP
Alshaer 2022	Prospective observational	Meropenem	Unicentric	Austria	12	PK/PKPOP
Kumta 2022	Prospective observational	Meropenem	Multicentric	China y Australia	8	PK/PKPOP
Fukumoto 2023	Prospective observational	Meropenem	Unicentric	Japan	32	PK/PKPOP
Meenks 2023	Prospective observational	Flucloxacillin	Multicentric	Netherlands	31	PK/PKPOP
Tang 2023	Prospective observational	Polimixina B	Unicentric	China	105	PK/PKPOP
Wang 2023	Prospective observational	Teicoplanin	Unicentric	China	151	PK/PKPOP
Barreto 2023	Prospective observational	Cefepime	Unicentric	USA	120	PK/PKPOP
Facca 1998	Prospective observational	ceftizoxime	Unicentric	USA	72	PK/PKPOP
Frame 1999	Prospective observational	Ceftazidime	Unicentric	USA	39	PK/PKPOP
Dailly 2013	Prospective observational	Ertapenem	Unicentric	France	8	PK/PKPOP
Beumier 2013	Prospective observational	Vancomycin	Unicentric	Belgium	32	PK/PKPOP

O¨brink-Hansen 2015	Prospective observational	Piperacillin	Unicentric	Denmark	15	PK/PKPOP
Lin 2016	Prospective observational	Vancomycin	Unicentric	China	100	PK/PKPOP
Ide 2018	Prospective observational	Linezolid	Unicentric	Japan	27	PK/PKPOP
Kang 2020	Prospective observational	Cefpirome	Unicentric	South Korea	15	PK/PKPOP
Grensemann 2020	Prospective observational	Meropenem	Unicentric	Germany	19	PK/PKPOP
Liu 2020	Prospective observational	Vancomycin	Unicentric	China	25	PK/PKPOP
Ruiz-Ramos 2020	Prospective observational	Tigecycline	Unicentric	Spain	25	PK/PKPOP
Lin 2021	Prospective observational	Vancomycin	Unicentric	China	374	PK/PKPOP
Sima 2021	Prospective observational	Ciprofloxacin	Unicentric	Czech Republic	29	PK/PKPOP
Zhao 2022	Prospective observational	Meropenem	Unicentric	China	64	PK/PKPOP
Alsultan 2022	Prospective observational	Meropenem	Unicentric	Saudi Arabia	43	Exposure
Wang 2021	Prospective observational	Vancomycin	Unicentric	China	11	PK/PKPOP
Busse 2022	RCT	Meropenem	Unicentric	Germany	30	PK/PKPOP
Farkas 2022	Prospective observational	Genatmicin	Unicentric	USA	24	PK/PKPOP
Hahn 2021	Prospective observational	PTZ	Unicentric	South Korea	26	PK/PKPOP
Gijsen 2022	Prospective observational	Merpenem	Unicentric	Belgium	58	PK/PKPOP
Pressiat 2022	Prospective observational	Amikacin	Unicentric	France	39	PK/PKPOP
Alihodzic 2022	Prospective observational	Ciprofloxacin	Unicentric	Germany	17	PK/PKPOP
Šíma 2022	Prospective observational	Ciprofloxacin	Unicentric	Czech Republic	29	Exposure
Kang 2022	Prospective observational	Meropenem	Unicentric	Korea	13	PK/PKPOP
An 2023	Prospective observational	Meropenem	Unicentric	USA	130	PK/PKPOP
Bai 2023	Prospective observational	Imipenem	Unicentric	China	51	PK/PKPOP
Martínez-Casanova 2023	Prospective observational	PTZ	Unicentric	Spain	106	PK/PKPOP
An 2023	Prospective observational	Cefepime	Unicentric	USA	130	PK/PKPOP
Bilal 2023	Prospective observational	cefepime	Unicentric	Germany	14	PK/PKPOP
Ehmann 2019	Prospective observational	Meropenem	Unicentric	Germany	48	PK/PKPOP
Soraluce 2020	Prospective observational	Linezolid	Multicentric	Spain	40	PK/PKPOP
Lee 2021	Prospective observational	Meropenem	Unicentric	South Korea	30	PK/PKPOP
Dedkaew 2015	Prospective observational	Vancomycin	Unicentric	Thailand	138	PK/PKPOP
Seo 2023	Prospective observational	Cefepime	Unicentric	Korea	21	PK/PKPOP
Mattioli 2016	Prospective observational	Meropenem	Unicentric	Italy	27	PK/PKPOP
Abdulla 2020	Prospective observational	Ciprofloxacin	Multicentric	Netherlands	42	PK/PKPOP
Cheng 2022	Prospective observational	Ciprofloxacin	Multicentric	Australia, New Zealand, South Korea, and Switzerland	8	PK/PKPOP
Isla 2008	Prospective observational	Meropenem	Multicentric	Spain	20	PK/PKPOP

Alobaid 2017	Prospective observational	Piperacillin	Unicentric	Australia	37	Exposure
Kim 2022	Prospective observational	PTZ	Unicentric	South Korea	38	PK/PKPOP
Ernest 1992	Prospective observational	Genatmicin	Unicentric	Australia	5	PK/PKPOP
Shikuma 1990	Prospective observational	Piperacillin	Unicentric	USA	11	PK/PKPOP
Cornwell 1997	Prospective observational	Aztreonam	Unicentric	USA	28	PK/PKPOP
Giles 2000	Prospective observational	Meropenem	Unicentric	UK	10	Exposure
Barletta 2000	Prospective observational	Genatamicin, tobramicin	Unicentric	USA	19	Exposure
Malone 2001	Prospective observational	Cefepime	Unicentric	USA	12	PK/PKPOP
Traunmüller 2002	Prospective observational	Ceftazidime	Unicentric	Austria	12	PK/PKPOP
Uchino 2002	Prospective observational	Vancomycin	Unicentric	Australia	7	PK/PKPOP
Fiaccadori 2004	Prospective observational	Linezolid	Unicentric	Italy	15	Exposure
Meyer 2005	Prospective observational	Linezolid	Unicentric	Austria	20	PK/PKPOP
Fish 2005	Prospective observational	Imipenem	Unicentric	USA	12	Exposure
Kielstein 2006	Prospective observational	Vancomycin, Meropenem	Unicentric	Germany	10	PK/PKPOP
Mimoz 2006	Prospective observational	Vancomycin	Unicentric	Thailand	20	PK/PKPOP
Bracco 2008	Prospective observational	Tobramicina	Unicentric	Canada	287	PK/PKPOP
Burkhardt 2009	Prospective observational	Ertapenem	Unicentric	Germany	6	Exposure
Golestaneh 2009	Prospective observational	Vancomycin	Unicentric	USA	10	Exposure
Deshpande 2010	Prospective observational	Meropenem	Unicentric	USA	10	Exposure
Bilgrami 2010	Prospective observational	Meropenem	Unicentric	Australia	10	PK/PKPOP
Taccone 2010	Prospective observational	Piperacillin-tazobactam (n = 27), ceftazidime (n = 18), cefepime (n = 19) or Meropenem (n = 16).	Multicentric	Belgium	80	Exposure
Seyler 2011	Prospective observational	Meropenem,PTZ, cefepime, ceftazidime	Unicentric	Belgium	53	Exposure
Baptista 2012	Prospective observational	Vancomycin	Unicentric	Portugal	93	Exposure
Roberts 2012	Prospective observational	Ciprofloxacin, Meropenem, piperacillin/tazobactam , or vancomycin	Multicentric	Australia	24	Exposure
Petejova 2012	Prospective observational	Vancomycin	Unicentric	Czech Republic	17	PK/PKPOP
D'Arcy 2012	Prospective observational	Amikacin	Unicentric	Ireland	5	PK/PKPOP
Binder 2013	Prospective observational	Meropenem	Unicentric	Germany	25	PK/PKPOP
Adnan 2013	Prospective observational	Meropenem, Piperacillin	Unicentric	Australia	10	Exposure
Carlier 2013	Prospective observational	Meropenem, Piperacillin	Unicentric	Belgium	61	Exposure
Sturm 2013	Prospective observational	PTZ	Unicentric	USA	9	Exposure
Carlier 2014	Prospective observational	PTZ	Unicentric	Belgium	11	Exposure
Huttner 2015	Prospective observational	imipenem, Meropenem, PTZ, cefepime	Unicentric	Switzerland	100	Exposure
Sime 2015	RCT	Piperacillin	Unicentric	Australia	32	Exposure

Awissi 2015	Prospective observational	PTZ	Unicentric	Canada	22	PK/PKPOP
Villa 2015	Prospective observational	Linezolid	Unicentric	Italy	3	PK/PKPOP
Wen 2016	Prospective observational	Imipenem	Unicentric	China	10	Exposure
Boucher 2016	Prospective observational	imipenem	Unicentric	USA	10	Exposure
Jung 2017	Prospective observational	PTZ	Unicentric	France	23	PK/PKPOP
Roger 2017	Prospective observational	PTZ	Unicentric	France	12	PK/PKPOP
Carrie 2017	Prospective observational	cefazolin, cefotaxime, PTZ, cefepime, ceftazidime y Meropenem	Unicentric	France	79	Exposure
Ruiz-Ramos 2018	Prospective observational	Amikacin	Unicentric	Spain	30	Exposure
Fournier 2018	RCT	Amoxicillin, Meropenem, Flucloxacillin, piperacillin-tazobactam, Ceftriaxone, Imipenem/cilastatin, Ceftazidime, Ertapenem, Cefazolin.	Unicentric	Switzerland	45	Exposure
Sinnollareddy 2018	Prospective observational	Piperacillin	Unicentric	Australia	6	PK/PKPOP
Wang 2018	Prospective observational	Teicoplanin	Unicentric	China	18	Exposure
Kassel 2018	Prospective observational	Vancomycin, cefepime	Unicentric	USA	20	Exposure
Olbrisch 2019	Prospective observational	Piperacillin	Unicentric	Germany	36	Exposure
Barrassa 2019	Prospective observational	Linezolid	Multicentric	Spain	21	Exposure
Schmidt 2019	Prospective observational	Colistina	Unicentric	Germany	4	Exposure
Singhan 2019	Prospective observational	Meropenem	Unicentric	Thailand	15	PK/PKPOP
Bougle 2019	Prospective observational	Amoxicillin Piperacillin Cefotaxime Ceftazidine Cefepime Meropenem Imipenem Vancomycin Ciprofloxacin Genatmicin Tobramicina Amikacin	Unicentric	France	44	Exposure
Dhaese 2019	Prospective observational	PTZ, Meropenem	Unicentric	Belgium	253	Exposure
Leon 2020	Prospective observational	PTZ, Meropenem, imipenem, Ceftriaxone, cefotaxima	Unicentric	France	48	Exposure
Roberts 2021	Prospective observational	Meropenem, piperacillin/tazobactam , vancomycin	Multicentric	14 paises	381	Exposure
Gieling 2020	Prospective observational	Ciprofloxacin	Multicentric	Netherlands	39	Exposure
Moni 2020	Prospective observational	Colistina	Unicentric	India	20	Exposure
Lin 2021	Prospective observational	Vancomycin	Unicentric	Taiwan	17	Exposure
Abdulla 2020	Prospective observational	Amoxicillin, cefotaxime, ceftazidime, ceftriaxone, cefuroxime, meropenem.	Multicentric	Netherlands	147	Exposure
Moreira de Freitas 2020	Prospective observational	Vancomycin	Unicentric	Brazil	55	Exposure

Kühn 2020	Prospective observational	Piperacillin, ceftazidime, meropenem, linezolid	Unicentric	Germany	86	Exposure
Corcione 2021	Prospective observational	Amikacin, Genatmicin	Unicentric	Italy	8	Exposure
Petersson 2021	Prospective observational	Meropenem, Piperacillin	Unicentric	Sweden	135	Exposure
Nicolau 2021	Prospective observational	Ceftolozane/tazobacta m	Multicentric	Europe and USA	11	Exposure
Fillâtre 2021	Prospective observational	Piperacillin	Unicentric	France	42	Exposure
Moser 2021	Prospective observational	Flucloxacillin	Unicentric	Switzerland	50	Exposure
Esteve-Pitarch 2021	Prospective observational	PTZ, Meropenem	Unicentric	Spain	118	Exposure
Černá Pařízková 2021	Prospective observational	Meropenem	Unicentric	Czech Republic	25	Exposure
Liebchen 2022	Prospective observational	Piperacillin, Meropenem	Unicentric	Germany	17	Exposure
Messiano 2022	Prospective observational	Meropenem	Unicentric	Brazil	15	Exposure
Zoller 2022	Prospective observational	Linezolid	Unicentric	Germany	68	Exposure
Shekar 2023	Prospective observational	Caspofungin,cefepime, ceftriaxone, ciprofloxacin, linezolid, meropenem, piperacillin, vancomycin	Multicentric	New Zealand and Switzerland	85	Exposure
Smeets 2023	Prospective observational	Vancomycin	Unicentric	Netherlands	20	Exposure
Correia 2023	Prospective observational	Ceftazidime	Multicentric	France	98	Exposure
Martin 1991	Prospective observational	Ofloxacin	Unicentric	France	12	PK/PKPOP
Akers 2014	Prospective observational	PTZ	Unicentric	USA	13	PK/PKPOP
Gomez 2015	Prospective observational	Imipenem	Unicentric	Brazil	51	PK/PKPOP
Ko 2016	Prospective observational	Vancomycin	Unicentric	USA	263	Exposure
Mokline 2018	Prospective observational	Linezolid	Unicentric	Tunisia	13	Exposure
Lim 2019	Prospective observational	Teicoplanin	Unicentric	South Korea	8	PK/PKPOP
Kovacevic 2019	Prospective observational	Vancomycin	Unicentric	Bosnia and Herzegovina	61	Exposure
Wu 2019	Prospective observational	Piperacillin/tazobactam , cefepime, and Meropenem	Unicentric	Taiwan	100	Exposure
Morbitzer 2019	Prospective observational	Vancomycin	Unicentric	USA	17	Exposure
Breilh 2019	RCT	PTZ, Meropenem, ertapenem, imipenem, doripenem, Ceftriaxone, ofloxacin, linezolid, daptomycin, metronidazol	Multicentric	Belgium and the Netherlands	45	PK/PKPOP
Oliveira 2020	Prospective observational	Meropenem, Vancomycin	Unicentric	Brazil	30	Exposure
Mahmoud 2020	Prospective observational	Vancomycin	Unicentric	USA	8	Exposure
Veillette 2021	Prospective observational	PTZ	Unicentric	USA	29	Exposure
Gijsen 2021	Prospective observational	Ceftriaxone	Unicentric	Belgium	31	Exposure
Shi 2022	Prospective observational	Teicoplanin	Unicentric	China	106	Exposure
Zhao 2022	Prospective observational	Vancomycin	Multicentric	China	414	Exposure

Calov 2023	Prospective observational	Daptomycin	Unicentric	Germany	13	Exposure
Tikiso 2023	Prospective observational	Linezolid	Unicentric	Germany	18	PK/PKPOP
Roberts 2015	RCT	Ciprofloxacin, meropenem, PTZ, vancomycin	Multicentric	Australia	24	PK/PKPOP
Scharf 2022	Prospective observational	Vancomycin	Unicentric	Germany	7	Exposure
Wulkersdorfer 2024	Prospective observational	PTZ	Unicentric	Austria	7	PK/PKPOP
Shahrami 2020	Prospective observational	Amikacin	Unicentric	Iran	37	PK/PKPOP
Simon 2021	RCT	Linezolid	Unicentric	Germany	30	Exposure
Del Bono 2017	Prospective observational	Meropenem	Unicentric	Italy	19	Exposure
Wu 2022	Prospective observational	Linezolid	Unicentric	China	23	Exposure
Corti 2013	Prospective observational	Daptomycin	Unicentric	Switzerland	9	PK/PKPOP
Corcione 2020	Prospective observational	Meropenem	Unicentric	Italy	17	Exposure
Lyu 2018	RCT	PTZ	Unicentric	China	120	Exposure
Van der Starre 2010	Prospective observational	Vancomycin PPX	Unicentric	USA	24	Exposure
Triginer 1990	Prospective observational	Genatmicin	Unicentric	Spain	40	PK/PKPOP
Tang 1999	Prospective observational	Genatmicin	Unicentric	Taiwan	52	PK/PKPOP
Rebuck 2002	Prospective observational	Levofloxacin	Unicentric	USA	30	PK/PKPOP
Belzberg 2004	Prospective observational	Imipenem	Unicentric	USA	54	PK/PKPOP
Sanchez-Navarro 2005	Prospective observational	Levofloxacin	Unicentric	Spain	9	PK/PKPOP
Van Zanten 2008	Prospective observational	Ciprofloxacin	Unicentric	Netherlands	32	Exposure
Brink 2009	Prospective observational	Ertapenem	Unicentric	South Africa	8	Exposure
Chapuis 2010	Prospective observational	Cefepime	Unicentric	Switzerland	21	PK/PKPOP
Aubron 2011	Prospective observational	Vancomycin	Unicentric	Australia	48	PK/PKPOP
Chung 2011	Prospective observational	Vancomycin	Unicentric	Korea	141	Exposure
Karnik 2013	Prospective observational	Colistin	Unicentric	India	15	PK/PKPOP
Koegelenberg 2013	Prospective observational	Rifampicin, isoniazide, etambutol	Unicentric	South Africa	10	Exposure
de Montmollin 2014	Prospective observational	Amikacin	Unicentric	France	146	Exposure
Nakamura 2015	Prospective observational	Teicoplanin	Unicentric	Japan	106	Exposure
Abdul-Aziz 2016	Prospective observational	Meropenem, PTZ	Multicentric	Spain	211	Exposure
Zander 2016	Prospective observational	Piperacillin	Unicentric	Germany	60	Exposure
Allou 2016	Prospective observational	Genatmicin	Unicentric	France	57	Exposure
Obara 2016	Prospective observational	Vancomycin	Unicentric	Brazil	83	Exposure
Bakke 2017	Prospective observational	Vancomycin	Multicentric	Norway	83	Exposure
Ruiz-ramos 2018	Prospective observational	Amikacin	Unicentric	Spain	85	Exposure
Burger 2018	Prospective observational	Meropenem	Unicentric	Switzerland	101	Exposure
Huang 2020	Prospective observational	Imipenem	Unicentric	China	25	PK/PKPOP

Helset 2020	Prospective observational	Vancomycin	Unicentric	Norway	83	Exposure
Ram 2021	Prospective observational	Colistina	Unicentric	India	30	Exposure
Ren 2021	Prospective observational	Vancomycin	Multicentric	USA	7220	Exposure
Niibe 2022	Prospective observational	Meropenem	Unicentric	Japan	12	PK/PKPOP
Mitton 2022	Prospective observational	Imipenem	Unicentric	South Africa	68	Exposure
Helset 2024	Prospective observational	Meropenem	Unicentric	Norway	87	Exposure
Areskog Lejbman 2024	Prospective observational	Cefotaxime, PTZ, Meropenem	Unicentric	Sweden	102	Exposure
Beckhouse 1988	Prospective observational	Gentamicin, tobramycin, amikacin	Unicentric	USA	49	PK/PKPOP
Zeitlinger 2007	Prospective observational	Levofloxacin, fosfomycin, cefpirome	Unicentric	Austria	34	Exposure
Cristallini 2016	Prospective observational	Vancomycin	Unicentric	Belgium	107	Exposure
Ehmann 2017	Prospective observational	Meropenem	Unicentric	Germany	48	Exposure
Costea 2020	Prospective observational	Amikacin, Genatmicin	Unicentric	France	138	Exposure
Logre 2020	Prospective observational	Amikacin	Multicentric	France	93	Exposure
De Corte 2023	Prospective observational	Meropenem, PTZ	Unicentric	Belgium	781	Exposure
Guilhaumou 2023	Prospective observational	Cefepime, cefotaxime, ceftazidime, meropenem	Multicentric	France	170	Exposure
El-Haffaf 2024	Prospective observational	PTZ	Unicentric	Canada	43	Exposure
Campassi 2014	Prospective observational	Vancomycin	Unicentric	Argentina	363	Exposure
Zeng 2024	Prospective observational	Polimixina B	Unicentric	China	23	Exposure
Galar 2019	Prospective observational	Daptomycin	Unicentric	Spain	63	Exposure
Obara 2016	Prospective observational	Vancomycin	Unicentric	Brazil	83	Exposure
Frazee 2017	Prospective observational	Vancomycin	Unicentric	USA	399	Exposure
You 2024	Prospective observational	Imipenem, meropenem	Unicentric	China	186	Exposure
Huang 2023	Prospective observational	Tigecycline	Unicentric	China	17	Exposure
Poli 2019	Prospective observational	Clindamycin	Unicentric	Switzerland	1	Exposure
Liebchen 2021	Prospective observational	Vancomycin, Teicoplanin	Unicentric	Germany	3	Exposure
Köhler 2022	Prospective observational	Linezolid	Unicentric	Germany	1	Exposure
Dimski 2020	Prospective observational	Meropenem	Unicentric	Germany	25	Exposure

BMI: Body mass index; CrCl: Cretatinine clearance; EGFR: Estimated glomerular filtration rate; PK/PKPOP: Population pharmacokinetic model (PKPOP); PTZ: Piperacillin-Tazobactam; RRT: Renal replacment therpay; TBW: Total body weight
